# Human prostaglandin reductases dearomatize and inactivate benzothiazinone antitubercular drugs

**DOI:** 10.1038/s44321-026-00526-1

**Published:** 2026-10-07

**Authors:** Myriam Briki, Caroline A Wagner, Vincent Desfontaine, Anthony Vocat, Onya Opota, Sarah Abou-Zite, Océane Corpataux, Benedetta Cena, Sylvie Guinchard, François Versace, Amarande Murisier, Thomas Mercier, Emilyne Blattes, Carine Bardinet, Paul Thoueille, Vladimir Wsol, Lenka Lastovickova, Haithem Chtioui, Laura E Rothuizen, Aimable Nahimana, Axel Bellotti, Laurent R Chiarelli, Dany Spaggiari, Neeraj Dhar, Richa Mishra, Raphaël Sommer, Andréanne Lupien, Michel A Duchosal, Benoît Guery, François R Girardin, Giuseppina Milano, Olga Riabova, Vadim Makarov, Thierry Buclin, Stewart T Cole, Eva Choong, Laurent A Decosterd

**Affiliations:** 1 Laboratory & Service of Medical Pharmacology, Department of Laboratory Medicine and Pathology, and Department of Medicine, University Hospital of Lausanne and University of Lausanne, Lausanne, Switzerland; 2 Institute of Microbiology, Department of Laboratory Medicine and Pathology, University Hospital of Lausanne and University of Lausanne, Lausanne, Switzerland; 3 Innovative Medicines for Tuberculosis (iM4TB), Lausanne, Switzerland; 4 Faculty of Pharmacy in Hradec Králové, Charles University, Prague, Czech Republic; 5 Service and Central Laboratory of Hematology, University Hospital of Lausanne and University of Lausanne, Lausanne, Switzerland; 6 Department of Biology and Biotechnology, University of Pavia, Pavia, Italy; 7 Global Health Institute, School of Life Sciences, EPFL, Lausanne, Switzerland; 8 Service of Infectious Diseases, University Hospital of Lausanne and University of Lausanne, Lausanne, Switzerland; 9 Service of Cardiac Surgery, University Hospital of Lausanne and University of Lausanne, Lausanne, Switzerland; 10 Federal Research Center “Fundamentals of Biotechnology RAS”, Moscow, Russia; 11 Present Address: DSM-Firmenich, Geneva, Switzerland; 12 Present Address: Centre for Research and Innovation in Clinical Pharmaceutical Sciences (CRISP), Lausanne University Hospital (CHUV), Lausanne, Switzerland; 13 Present Address: Institute of Family Medicine, Faculty of Science and Medicine, University of Fribourg, Fribourg, Switzerland; 14 Present Address: Office de la Consommation, Etat de Vaud, Switzerland; 15 Present Address: Merck Group, Corsier-sur-Vevey, Switzerland; 16 Present Address: Debiopharm International SA, Lausanne, Switzerland; 17 Present Address: Vaccine and Infectious Disease Organization (VIDO), University of Saskatchewan, Saskatoon, SK Canada; 18 Present Address: Department of Biochemistry, Microbiology and Immunology, College of Medicine, University of Saskatchewan, Saskatoon, SK Canada; 19 Present Address: Vaccinology & Immunotherapeutics Program, School of Public Health, University of Saskatchewan, Saskatoon, SK Canada; 20 Present Address: Department of Infectious Diseases, Medical Microbiology and Hygiene, Medical Faculty Heidelberg, Heidelberg University, Heidelberg, Germany; 21 Present Address: Department of Medicine and Department of Microbiology and Immunology, Research Institute of the McGill University Health Centre, McGill International TB Centre, McGill University, Montreal, QC Canada; 22 Present Address: University of Lausanne, Lausanne, Switzerland; 23 Present Address: Institut Pasteur, Paris, France

**Keywords:** Microbiology, Virology & Host Pathogen Interaction, Pharmacology & Drug Discovery

## Abstract

Macozinone (MCZ, PBTZ169) is a potent clinical-stage benzothiazinone antitubercular agent that covalently inhibits the essential mycobacterial flavoenzyme DprE1. In some mammals, MCZ undergoes reductive dearomatization to H_2_MCZ, a Hydride Meisenheimer Complex, identified as the major circulating metabolite in humans. We demonstrate for the first time that the NADPH-dependent human prostaglandin reductases PTGR1 and PTGR2 catalyze MCZ dearomatization into H_2_MCZ, resulting in loss of antimycobacterial activity. This reaction represents a heretofore undescribed host-mediated metabolic inactivation pathway for a therapeutic agent. Although H_2_MCZ may constitute a transient reactive intermediate, ex vivo and cellular data indicate that it does not contribute to antimycobacterial activity in vivo. Pharmacological inhibition of PTGR1 and PTGR2 using diclofenac, indomethacin, dicumarol, or the selective inhibitor PTGR2-IN-1 suppresses H_2_MCZ formation and partially restores MCZ antimycobacterial activity in vitro. Together, these findings uncover a previously unrecognized noncanonical enzymatic mechanism of drug metabolism involving dearomatization in humans. Targeting prostaglandin reductases may represent a strategy to enhance benzothiazinone exposure and efficacy.

## Synopsis

A previously unrecognized host metabolic pathway was identified in which human prostaglandin reductases PTGR1 and PTGR2 inactivate the antitubercular benzothiazinone drug macozinone (MCZ) by reductive dearomatization, revealing a mechanism that limits efficacy and may be therapeutically targeted.

- PTGR1 and PTGR2 were identified as the human NADPH-dependent enzymes responsible for reductive dearomatization of MCZ to H_2_MCZ.
- A previously unrecognized host-mediated drug metabolism pathway was recognized for a clinical-stage benzothiazinone, expanding the known functions of human prostaglandin reductases.
- In vitro and ex vivo studies show that H_2_MCZ does not contribute to antimycobacterial activity in vivo, indicating that the PTGR1/2 pathway inactivates MCZ.
- H_2_MCZ formation was suppressed by diclofenac, indomethacin, dicumarol, and PTGR2-IN-1, and MCZ activity was partially restored in vitro.
- PTGR1 and PTGR2 were identified as potential host-directed pharmacological targets for improving benzothiazinone exposure and therapeutic efficacy against tuberculosis.

## Abstract (web summary)

A previously unrecognized host metabolic pathway was identified in which human prostaglandin reductases PTGR1 and PTGR2 inactivate the antitubercular benzothiazinone drug macozinone (MCZ) by reductive dearomatization, revealing a mechanism that limits efficacy and may be therapeutically targeted.

## Graphical abstract

**Figure:**
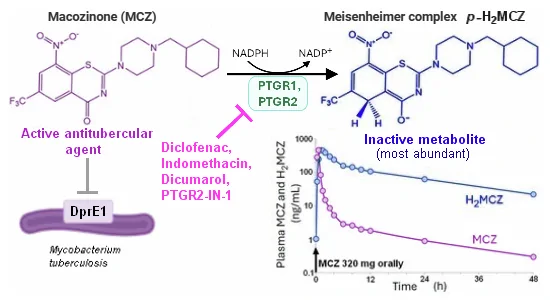

The paper explained **Problem**

Macozinone (MCZ; PBTZ169) is a promising antituberculosis benzothiazinone drug currently in clinical development. It is extensively metabolized in humans to a reduced metabolite, H_2_MCZ. The enzymatic pathways responsible for this metabolism were unknown.

**Results**

We identified the NADPH-dependent human prostaglandin reductases PTGR1 and PTGR2 as the enzymes that convert MCZ into H_2_MCZ via reductive dearomatization (Meisenheimer Complex reaction). In vitro and ex vivo studies show that H_2_MCZ does not contribute to antimycobacterial activity in vivo, indicating that the PTGR1/2 pathway inactivates MCZ. Pharmacological inhibition of PTGR1/2 reduces H_2_MCZ formation and enhances MCZ antimycobacterial activity in vitro.

**Impact**

This mechanism of drug metabolism, newly described in mammals, results in the direct inactivation of the antituberculosis agent MCZ. The PTGR1/2 enzymatic pathway could represent a host-related target for improving benzothiazinone exposure and therapeutic efficacy.

## Introduction

Tuberculosis (TB) remains one of the world’s deadliest diseases, causing 1.25 million deaths in 2023, making it the leading cause of death from a single infectious agent (surpassing COVID-19) WHO (2024). Even more alarming is the growing number of multidrug-resistant TB (MDR-TB) cases, defined as resistant to rifampicin and isoniazid. Among the emerging treatments against both susceptible- and MDR-TB, benzothiazinones constitute an attractive class, showing potent antimycobacterial activity by covalently binding to the critical flavoenzyme decaprenylphosphoryl-β-D-ribose 2-epimerase (DprE1). The resultant blockade of arabinan polymer synthesis disrupts the mycobacterial cell wall (Makarov et al, 2009; Neres et al, 2012; Trefzer et al, 2012) and induces cell lysis.

**Figure 1:**
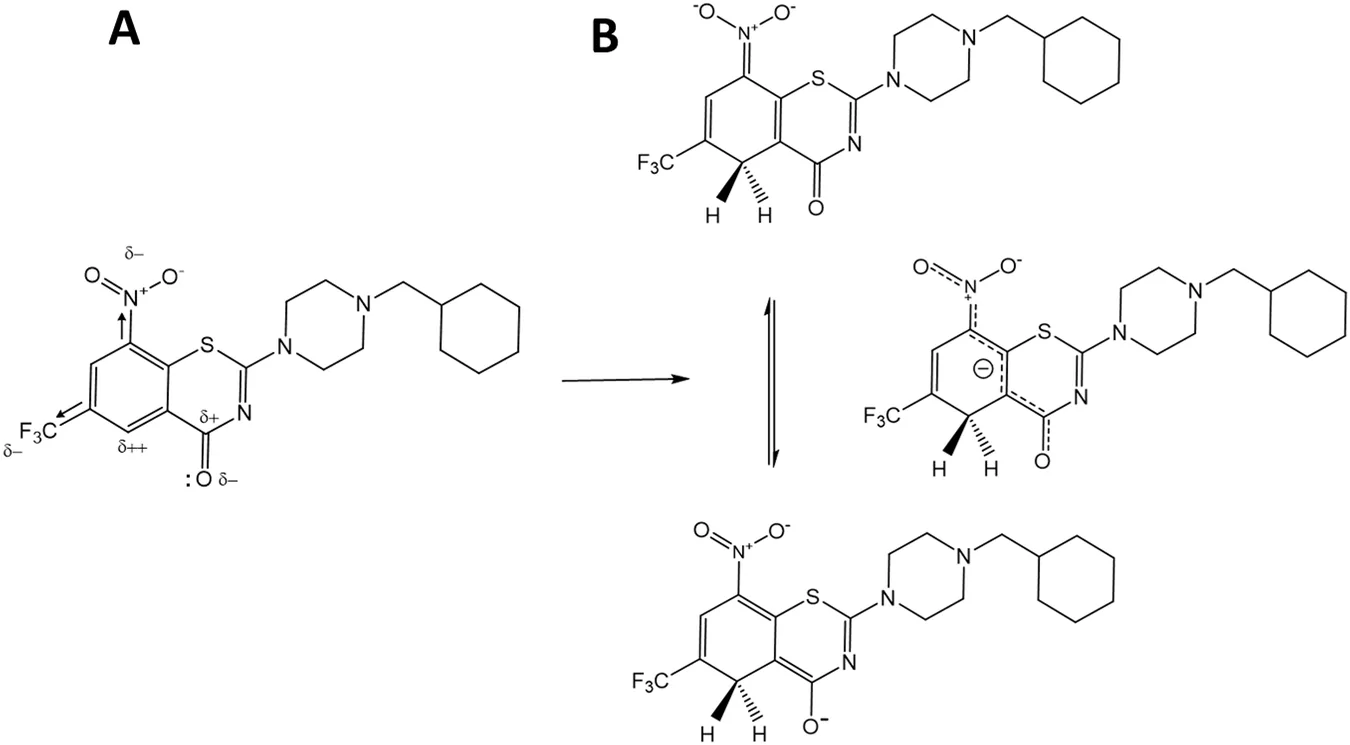
Chemical structures of macozinone and its reduced metabolite H_2_MCZ. (**A**) Macozinone (MCZ; PBTZ169) showing the relevant electrochemical features of the molecule. (**B**) The reduced, dearomatized, metabolite p-H_2_MCZ depicted as the two mesomeric forms in equilibrium.

Macozinone (MCZ), also known as PBTZ169 (Fig. 1A), is a promising, highly potent benzothiazinone derivative developed after chemical optimization from the lead compound BTZ-043 (Makarov et al, 2014). It has proven active against susceptible and drug-resistant *Mycobacterium tuberculosis* strains with minimum inhibitory concentration (MIC) values below 0.0005 μg/mL (Lupien et al, 2018). Its effectiveness has been confirmed in vivo, notably in guinea pig and mouse models (Makarov et al, 2014).

Both MCZ and BTZ-043 are reductively activated to form a nitroso moiety that reacts with a key cysteine (Cys387) located in the active site of DprE1. This generates a semi-mercaptal that results in covalent inhibition (Neres et al, 2012).

MCZ has successfully passed preclinical safety, toxicology, pharmacokinetic as well as antimycobacterial activity assessments in Phase I and II clinical trials that took place both in Russia and in Switzerland (https://clinicaltrials.gov/study/NCT03423030; https://clinicaltrials.gov/study/NCT03776500). In parallel, BTZ-043, following tissue distribution studies in murine models, (Rompp et al, 2025) has reached Phase II trials, which demonstrated an excellent safety profile and a bactericidal activity in the range of rifampicin at a dose of 10 mg/kg (Heinrich et al, 2025).

In a seminal publication (Kloss et al, 2017), Kloss et al reported that benzothiazinones (both MCZ and BTZ-043) follow an unprecedented metabolic pathway yielding a Hydride Meisenheimer Complex (HMC), which represents the first example of HMC formation in humans, and more generally in mammals. Specifically, MCZ undergoes bioreductive dearomatization in vivo, producing the peculiar metabolite *para*-H_2_MCZ (*p-*H_2_MCZ) (Fig. 1B, depicted as the two mesomeric forms), which may revert to the parent compound in the presence of oxygen (Kloss et al, 2017).

The regioselective reduction to *p*-H_2_MCZ indicates that the reaction is carried out enzymatically (Kloss et al, 2017): whereas chemical reduction by NaBH_4_ yields a mixture of *ortho*- and *para-*H_2_MCZ (*o-* and *p-*H_2_MCZ, respectively), the latter being the sole species observed in vivo. Explanations for this unprecedented enzymatic reaction, and its consequences for antimycobacterial activity, are lacking despite H_2_MCZ, which accumulates after multiple doses (Desfontaine et al, 2023), being the most abundant metabolite circulating in rats and humans (Spaggiari et al, 2019).

The FADH_2_/FAD redox chemistry in the DprE1 active site mediates reduction of the nitro group of benzothiazinone drugs into the reactive nitroso moiety. A potential role for the intermediate HMC metabolite has been suggested in this reaction (Liu et al, 2021), considering that H_2_MCZ needs only to lose a water molecule to generate the corresponding, putatively active nitroso species (Liu et al, 2021). Although disappointing to date, the search continues for benzothiazinones with a reduced HMC formation propensity that retain potent antimycobacterial activity (Joch et al, 2024; Keiff et al, 2024).

The aims of the present study were (i) to identify the enzyme(s) responsible for the in vivo dearomatization of MCZ into the HMC H_2_MCZ; (ii) to study the consequence of this metabolism on antimycobacterial activity; and (iii) to assess the impact of modulation of MCZ metabolism on its antimycobacterial activity.

We successively investigated various enzymatic systems likely to catalyze MCZ dearomatization, including carbonyl-reducing enzymes (NAD(P)H:quinone oxidoreductases, and reductases from the short-chain dehydrogenase/reductase and aldo-ketoreductase superfamilies) and thiol-based antioxidant enzymes (glutathione reductase, thioredoxin reductase, and glutathione transferase). We further extended our exploration to general enzymatic pathways mediating carbon–carbon double bond reduction (Huang et al, 2014) in microorganisms and mammals.

## Results

### Identification of the putative macozinone reductase(s)

Our investigations were guided by results from ex vivo incubation experiments in mammalian tissues with prototypical inhibitors of various known reductive enzymatic pathways (Appendix Table S1). Monitoring the rate of MCZ reduction to H_2_MCZ by mass spectrometry allowed us to determine the decrease in H_2_MCZ formation in the presence of inhibitors, as compared to controls. Any inhibition of the reductase systems under study opened the way to subsequent MCZ incubation experiments, ultimately with the respective pure recombinant human enzymes.

### Enzyme candidate screening

**Figure 2:**
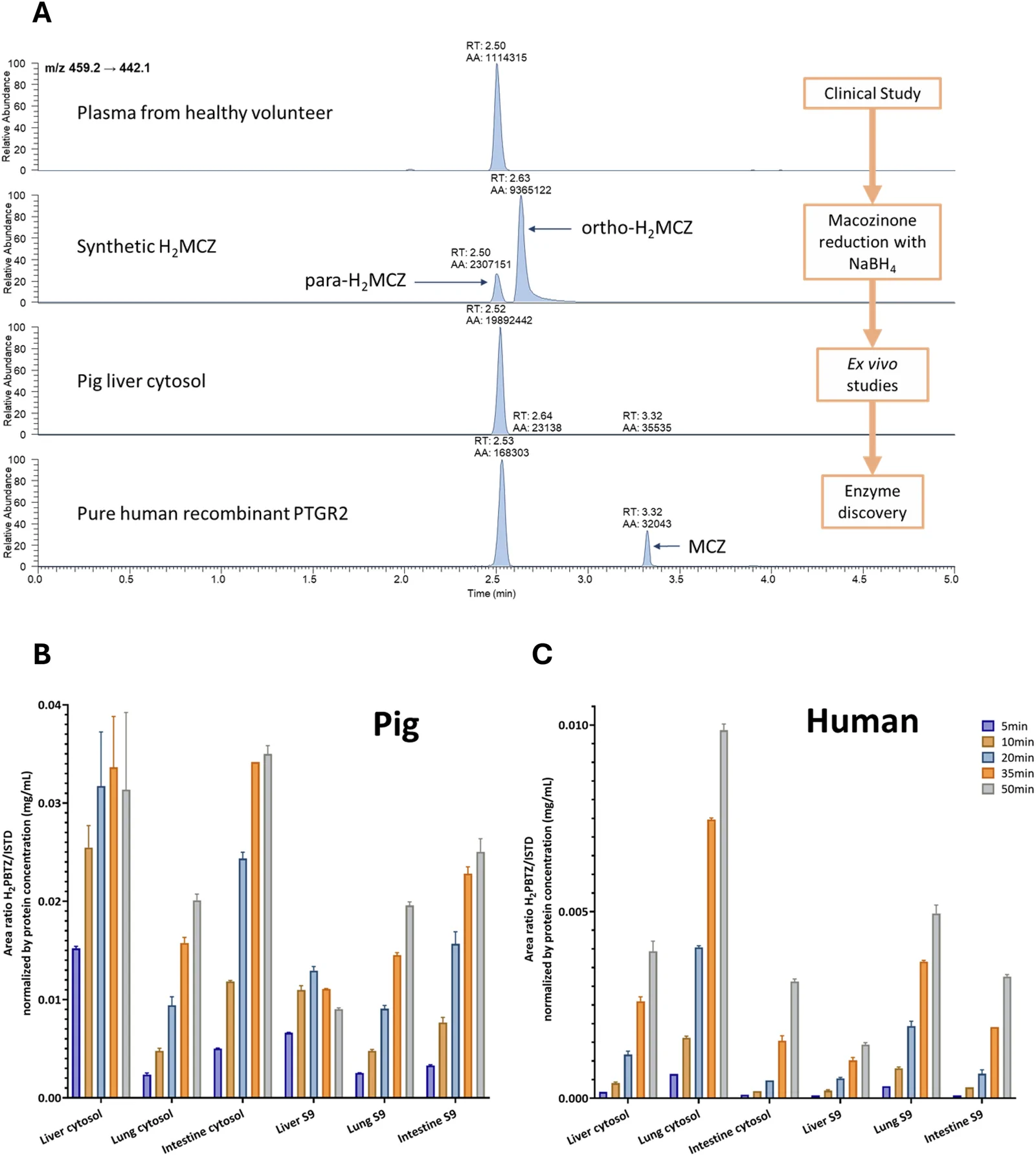
Biological systems that can reduce MCZ. (**A**) Chromatographic profiles of H_2_MCZ in various in vitro, ex vivo, and in vivo samples. Ex vivo incubation: H_2_MCZ production in (**B**) pig and (**C**) human cytosolic and S9 fractions; mean (*n* = 2) ± SD. No H_2_MCZ was formed in microsomes nor in mitochondria from tissues. Pig spleen cytosol and S9 fractions also yielded H_2_MCZ (not shown). Source data are available online for this figure.

To establish the screening assay, we first tested various biological systems for their ability to reduce MCZ into H_2_MCZ in vitro by simultaneously monitoring their presence by high-performance liquid chromatography coupled to tandem mass spectrometry (LC-MS/MS), using assays developed (Spaggiari et al, 2019) and further optimized (Desfontaine et al, 2023) in our laboratory. Typical chromatographic profiles of H_2_MCZ in various in vivo, in vitro, and ex vivo samples are shown in Fig. 2A.

Incubation of MCZ in human whole blood, peripheral blood mononuclear cells, or plasma did not produce appreciable levels of H_2_MCZ, if any. Alternatively, pig tissue extracts efficiently reduced MCZ into H_2_MCZ, as found in human volunteers (Fig. 2A). The identity of the metabolite produced was confirmed by LC-MS/MS as having the same MS/MS transitions and retention times as the *p-*H_2_MCZ made by NaBH_4_ treatment (Kloss et al, 2017) (Fig. 2A).

This reaction was found to preferentially use NADPH as a cofactor, and occurred in the S9 and cytosolic fractions, but not in microsomal fractions. The cytosolic reductase appears to be ubiquitous, as H_2_MCZ was detected after incubating MCZ with various pig organ extracts (liver, lungs, intestines, and spleen; Fig. 2B). In pig liver cytosol (PLC) at a MCZ concentration of 4.38 µM, the enzymatic reaction followed Michaelis–Menten kinetics with an apparent Km of 2.77 µM and a V_max_ of 7.46 ng/mL/min. After extensive characterization, this assay was applied throughout our investigation of MCZ reductase(s).

### Incubation of macozinone in human tissues

Ex vivo incubation experiments were also performed with MCZ incubated in S9 and cytosol fractions of post-mortem human liver, lung, and ileum tissue specimens. The results of H_2_MCZ production over 50 min are shown in Fig. 2C, and compared to the incubation results with pig tissues (Fig. 2B, left). Interestingly, the H_2_MCZ yield was highest in human lung S9 and cytosolic fractions, while in pig tissues, H_2_MCZ was mostly produced in the liver and intestine. As in pig, no H_2_MCZ was produced upon incubation with human microsomal and mitochondrial fractions, confirming that the reducing enzyme is cytosolic.

### Enzyme candidate screening

Building on current knowledge of the chemistry of MCZ and its HMC metabolite, the main categories of enzymes that we examined successively were: (i) carbonyl-reducing enzymes (CRE), (ii) enzymes participating in redox cellular homeostasis, (iii) enzymes implicated in nucleophilic aromatic substitution, and (iv) α,β-unsaturated ketone reductases. The enzymes investigated and their prototypical inhibitors are listed in Appendix Table S1.

#### Carbonyl reductases

As the MCZ carbonyl group is replaced in H_2_MCZ by an enolic hydroxyl group, we hypothesized that an initial reduction would take place, with subsequent tautomeric and mesomeric rearrangements. Several cytosolic NADPH-dependent enzymes could theoretically reduce MCZ, including the abundant cytosolic carbonyl reductase 1 (CBR1) from the short-chain dehydrogenases/reductases (SDR) superfamily, as well as several enzymes of the aldo-ketoreductase (AKR) superfamily (Malatkova et al, 2014; Malatkova and Wsol, 2014). However, incubation with the respective active recombinant enzymes CBR1, as well as AKR 1A1, 1B1, 1B10, and 1C3, yielded no H_2_MCZ.

Using the above optimized conditions, we then incubated MCZ in PLC in the presence of several representative inhibitors of the major reduction pathways reported in Appendix Table S1.

Dicumarol appeared to be the most potent, followed by curcumin, with IC_50_ of 0.107 and 12.8 µM, respectively. Lineweaver-Burk plots (not shown) were indicative of non-competitive inhibition. Dicumarol is the archetypal inhibitor of the drug-detoxifying enzyme NAD(P)H:quinone oxidoreductase 1 (NQO1, also known as DT-diaphorase) (Asher et al, 2006), a priori an attractive candidate because of its cytosolic location, use of NADPH as cofactor, and MCZ’s quinone-like structure. The NQO1 hypothesis was further supported by the marked decrease in H_2_MCZ production on incubation with curcumin (Tsvetkov et al, 2005), quercetin (Karakurt, 2015), as well as epigallocatechin-gallate (EGCG) (937.5 µM) (Pandey et al, 2020) and Cibacron Blue (CB) (16.1 µM) (Prochaska, 1988), all known NQO1 inhibitors (Appendix Table S1). However, incubation of MCZ with purified recombinant human NQO1, whose reducing activity was ascertained with its prototypical substrate duroquinone, failed to produce detectable levels of H_2_MCZ.

#### Enzymes participating in redox cellular homeostasis

The phenolic compounds curcumin (Fang et al, 2005), quercetin (Lu et al, 2006), and EGCG (Wang et al, 2008) have also been reported to inhibit the selenium-containing thioredoxin reductase I, TrxR1. The TrxR1 hypothesis was investigated by incubating MCZ with purified rat liver TrxR1 (with reducing activity confirmed using the model substrate 5,5-dithio-bis-(2-nitrobenzoic acid) (DTNB)), but this revealed no H_2_MCZ production. Moreover, specific gold-containing TrxR1 inhibitors aurothiomalate (ATM) (Omata et al, 2006) or auranofin (AF) (Gandin et al, 2010) did not affect H_2_MCZ production in PLC at 20 µM or 7.38 µM, respectively, thus excluding the involvement of TrxR1 in MCZ dearomatization.

The glutathione reductase (GR) pathway, inhibited by curcumin and quercetin (Guller et al, 2021), is also a key driver of redox cellular homeostasis, suggesting a possible implication in MCZ reduction. Also supporting this hypothesis, the formation of H_2_MCZ in PLC was inhibited by menadione (Duthie and Grant, 1989) and 2-AAPA (Seefeldt et al, 2009), two known inhibitors of glutathione reductase, with IC_50_ 15.5 µM and 30.6 µM, respectively. For redox cellular homeostasis, GR frequently functions in concert with other enzymes involved in glutathione metabolism (see below).

#### Enzymes implicated in nucleophilic aromatic substitution

Meisenheimer complexes typically constitute reactive intermediates of nucleophilic aromatic (S_N_Ar) substitutions (Meisenheimer, 1902). This occurs during the conjugation of reduced glutathione (GSH) by glutathione-S-transferase (GST) (Hayes et al, 2005) with the archetypal electrophilic substrate 1-chloro-2,4-dinitrobenzene (CDNB), yielding the final non-HMC 1-GS-2,4-dinitrobenzene (Tierbach et al, 2020). Conversely, in vitro experiments using mammalian GST gave HMCs as the final dead-end glutathionyl conjugate of 1,3,5-trinitrobenzene (TNB), as this compound lacks a leaving group at the *ipso* carbon (Bico et al, 1994). Such HMC metabolites of TNB have also been reported in vivo, e.g., in moth larvae (Clark and Sinclair, 1988) and octopus (Liou et al, 2000). In fungal species, the explosive 1,3,5-trinitrotoluene (TNT) is similarly metabolized into several HMC derivatives (Khilyas et al, 2013). The degradation of TNT into reduced HMC forms has also been reported in a *Mycobacterium* species (Vorbeck et al, 1994). The bacterial enzymatic pathway driving hydride addition to the aromatic ring of TNT is mediated by pentaerythritol tetranitrate reductase (PETNR) in *Enterobacter cloacae* (French et al, 1998) and by the xenobiotic reductase (XenB) in *Pseudomonas fluorescens I-C* (Pak et al, 2000). Remarkably and unusually, this is achieved by direct reduction (by enzyme-bound FMNH_2_) of the substrate aromatic ring to produce the HMC (French et al, 1998). By contrast, in humans, or more generally in mammals, isolable HMC products had never been reported in vivo for any xenobiotic metabolism before benzothiazinones (Kloss et al, 2017).

Given such considerations, we first verified that the gut microbiota was not responsible for a presystemic dearomatization of MCZ into H_2_MCZ, which, after intestinal absorption, would constitute the major circulating metabolite identified in rat and human plasma. However, ex vivo incubation of MCZ at 37 °C with feces from omnivorous and vegetarian human donors yielded no detectable H_2_MCZ. Incubation with mouse cecum content did not yield H_2_MCZ either.

Glutathione-S-transferase (GST) was of particular interest since these phase II enzymes play a key role in the detoxification of various endogenous and exogenous hydrophobic electrophilic compounds (Chasseaud, 1979). Also, GSTs are inhibited by dicumarol (Mays and Benson, 1992) and Cibacron blue (Ismail et al, 2021), both found to markedly decrease H_2_MCZ production in our PLC assay. The three electron-withdrawing groups (NO_2_, CF_3_, and ketonyl) in MCZ (Fig. 1A) create a strong electrophilic spot at the position *p-* to NO_2_, which may be conceivably deactivated into a putative glutathionyl-MCZ (MCZ-SG) intermediate. Screening for the presence of the putative MCZ-SG conjugate by incubating MCZ with PLC extracts was therefore performed by Orbitrap LC-HRMS in positive and negative ionization mode, by monitoring the *m/z* signals at 763.80 corresponding to the molecular weight of the putative glutathionyl-MCZ (C_30_H_40_F_3_N_7_O_9_S_2_)_._ However, in no case was detectable glutathionyl-MCZ found in the PLC (data not shown).

#### α,β-unsaturated ketone reductases

In addition to its ability to metabolize explosive nitro compounds, the bacterial NADPH-dependent reductase PETNR is remarkable for utilizing a series of α,β-unsaturated compounds (e.g., cyclic enone) as oxidizing substrates. These range from simple structures (e.g., cyclohexen-1-one) to complex α,β-unsaturated compounds (e.g., steroids) (Barna et al, 2001). Considering this intriguing interaction between bacterial PETNR and animal steroid hormones or their derivatives, we examined whether progesterone and 4-androstene-3,17-dione, two α,β-saturated steroid PETNR inhibitors (Barna et al, 2001), would interfere with a putative mammalian counterpart that might catalyze MCZ reduction. Both steroids, as well as the α,β-saturated hydrocortisone, were able to inhibit H_2_MCZ production in PLC, with IC_50_ values of ~50 µM (Appendix Table S1). Prednisone, another PETNR substrate, also inhibited to some extent the formation of H_2_MCZ in PLC, presumably by enzymatic competition.

The concerted electron-withdrawing effects of NO_2_- and CF_3_-groups render the position *para*- to NO_2_ (β relative to the keto group) highly electrophilic (Fig. 1A), thus making the double bond adjacent to the carbonyl group a virtual α,β-unsaturated carbonyl substrate (i.e., a ketone containing a carbon–carbon double bond adjacent to the carbonyl group, where the carbon directly next to the carbonyl carbon is the α-carbon and the following one is the β-carbon).

The enzymatic hydrogenation of endogenous or exogenous α,β-unsaturated carbonyl substrates (i.e., α,β-ketoalkene) can occur in mammals via a wide range of reactions (Huang et al, 2014). Enzymatic double-bond reduction of α,β-ketoalkene xenobiotics was reported for LY140091, a 2-benzylidine-3-ketocoumaran derivative (Lindstrom and Whitaker, 1984), for the aryl propionic NSAID agent CS-670 (Itoh et al, 2008), for the anticancer drug hydroxymethylacylfulvene (Yu et al, 2012), and, more recently, for the acetylcholinesterase inhibitor BYZX (Yu et al, 2013). For all these compounds, however, the double bond of the α,β-ketoalkene is not engaged in an aromatic cycle conjugation. The enzymatic double bond reduction of the CS-670 α,β-unsaturated ketone in rat liver cytosol was shown to be mediated by leukotriene B4 12-hydroxydeshydrogenase/15-ketoprostaglandinΔ13-reductase (LTB4 12-HD/PGR), later named prostaglandin reductase 1 (PTGR1) (Itoh et al, 2008). PTGR1, a highly inducible, cytosolic homodimeric enzyme, catalyzes the NADPH-dependent reduction of the 13,14 double bond (i.e., an α,β-unsaturated carbonyl) of prostaglandin 15-keto-PGE2 (Wang et al, 2021). PTGR1 plays a key role in the activation of antitumor acylfulvenes derivatives (Yu et al, 2012) and may also mediate double bond reduction in the α,β-ketoalkene compound BYZX (Yu et al, 2013).

Importantly, some of the widely used NSAIDs, which primarily act as cyclooxygenase inhibitors, also inhibit PTGR1 (Clish et al, 2001; Mesa et al, 2015). We thus tested the ability of three common NSAIDs, diclofenac, indomethacin, and niflumic acid, to inhibit MCZ dearomatization in PLC. These experiments showed that diclofenac and niflumic acid inhibited MCZ reduction with virtually the same IC_50_ (16.2 and 16.3 µM, respectively), while indomethacin was slightly less potent (IC_50_ 23.6 µM). The inhibition of MCZ reduction by NSAIDs strongly suggested that PTGR1 was a likely candidate for this dearomatization reaction. This assumption was also supported by the strong inhibition demonstrated in our PLC incubation experiments by dicumarol, another inhibitor of α,β-ketoalkene reductases (Yu et al, 2013).

### PTGR1 and PTGR2 mediate macozinone dearomatization

Definitive demonstration of PTGR1 mediating MCZ dearomatization was provided by incubating MCZ with pure recombinant human PTGR1, which yielded a single metabolite, *p*-H_2_MCZ, with the same chromatographic retention time and *m/z* transition as the H_2_MCZ produced by PLC incubation.

Human monomeric PTGR2 (also known as zinc-binding alcohol dehydrogenase domain-containing 1, ZADH1) shares 40% homology with PTGR1 (Mesa Solis, 2016). Like PTGR1, PTGR2 catalyzes NADPH-dependent reduction of the α,β-unsaturated carbonyl of 15-keto-PGE2, a key step of terminal inactivation of prostaglandins (Wu et al, 2008), but unlike PTGR1, it cannot oxidize the hydroxyl group of LTB4.

Incubation of MCZ with pure recombinant human PTGR2 also produced *p-*H_2_MCZ that had the same chromatographic retention time and *m/z* transition as the H_2_MCZ produced in PLC incubation (Fig. 2A). Recombinant PTGR2 provided a threefold higher yield of *p-*H_2_MCZ as compared to PTGR1 in the same incubation conditions.

**Figure 3:**
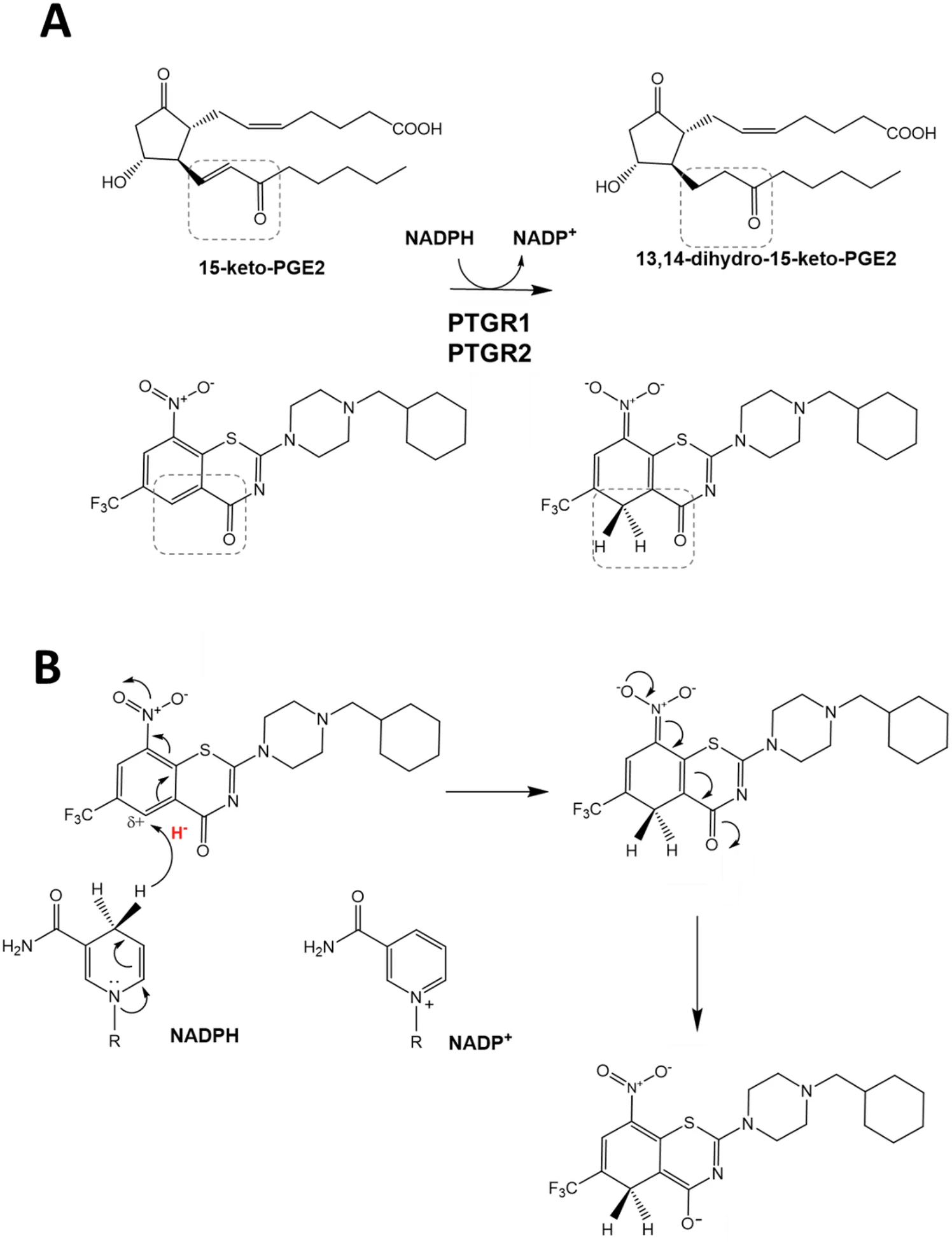
PTGR1 and PTGR2 are α,β-unsaturated ketone reductases that dearomatize MCZ into H_2_MCZ. (**A**) MCZ shares with prostaglandins PGE2 the α,β-unsaturated carbonyl moiety (dashed frame), owing to the presence of a strong electrophilic β carbon (H_2_MCZ shown in the keto form but can also be depicted as the enolate mesomere) (see Fig. 1). (**B**) Proposed mechanism of the NADPH-dependent reduction of MCZ by PTGR1 and PTGR2.

Therefore, though not obviously apparent on considering their chemical structures, MCZ shares with prostaglandins an α,β-unsaturated carbonyl group (Fig. 3A), which likely explains why this xenobiotic is a substrate of enzymes catalyzing the reduction of 15-keto-PGE2 prostaglandins. Such reaction is unique since it implies enzymatic β-reduction of a double bond engaged in an aromatic ring, creating the unprecedented HMC. The proposed biochemical reaction of NADPH-dependent reduction of MCZ by PTGR1 and PTGR2 into H_2_MCZ is shown in Fig. 3B.

### Confirmatory in vitro enzymatic inhibition studies

**Figure 4:**
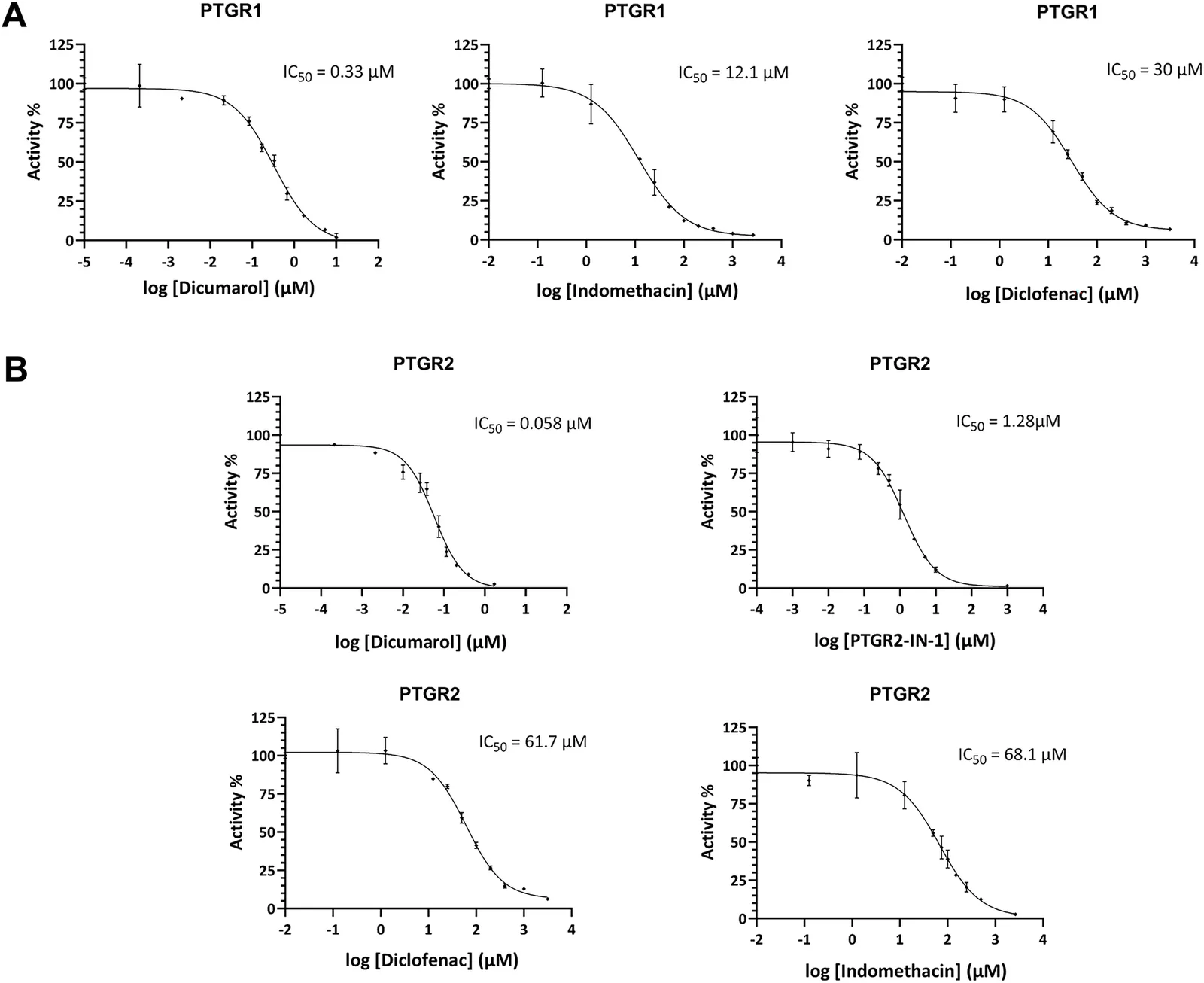
In vitro inhibition studies with human recombinant enzymes. Dearomatization mediated by (**A**) PTGR1 and (**B**) PTGR2, can be inhibited; mean (*n* = 2) ± SD. Source data are available online for this figure.

Using the recombinant PTGR1 and PTGR2, we compared the relative potency of various representative inhibitors on enzymatic reduction activity. Concentration response profiles for PTGR1 and PTGR2 inhibition are shown in Fig. 4A,B, respectively. Dicumarol was the most potent inhibitor against both PTGR1 and PTGR2 with IC_50_ values of 0.33 and 0.058 µM, respectively. Other NSAIDs such as diclofenac (Clish et al, 2001; Mesa et al, 2015; Mesa Solis, 2016) and indomethacin (Clish et al, 2001; Wu et al, 2008) also blocked PTGR1- and PTGR2-mediated MCZ reduction, as did the specific PTGR2 inhibitor PTGR2-IN-1 (Parker et al, 2017).

### Functional consequences of macozinone dearomatization

We then examined the antimycobacterial consequences of MCZ reduction through a combination of in vitro and ex vivo experiments, aiming to determine whether MCZ, its reduced metabolite, or both are responsible for antimycobacterial activity. The controversy about the uncertain role played by H_2_MCZ in overall in vivo antimycobacterial activity was fueled notably by Liu et al (Liu et al, 2021), who argued that MCZ may actually be a prodrug for the active electrophilic moiety H_2_MCZ.

### In vitro MIC determination

In vitro, the synthetic mixture of *o*- and *p*-H_2_MCZ revealed a potency slightly lower than MCZ against two *Mycobacterium* species (Appendix Fig. S1A), with an MIC of 1.2 µg/L against both *M. tuberculosis* H37Rv and *M. smegmatis* mc^2^155, compared with the MIC of 0.3 and respectively 0.6 µg/L known for MCZ in this assay.

Similarly, in vitro data with the BTZ-resistant MN84 strain (bearing a C394S mutation in DprE1) showed cross-resistance to MCZ and H_2_MCZ (Appendix Fig. S1B). Also, in *M. tuberculosis*-infected macrophage experiments, MCZ and H_2_MCZ were of similar potency, indicating that H_2_MCZ enters human cells (or at least macrophages; Appendix Fig. S1C).

**Figure 5:**
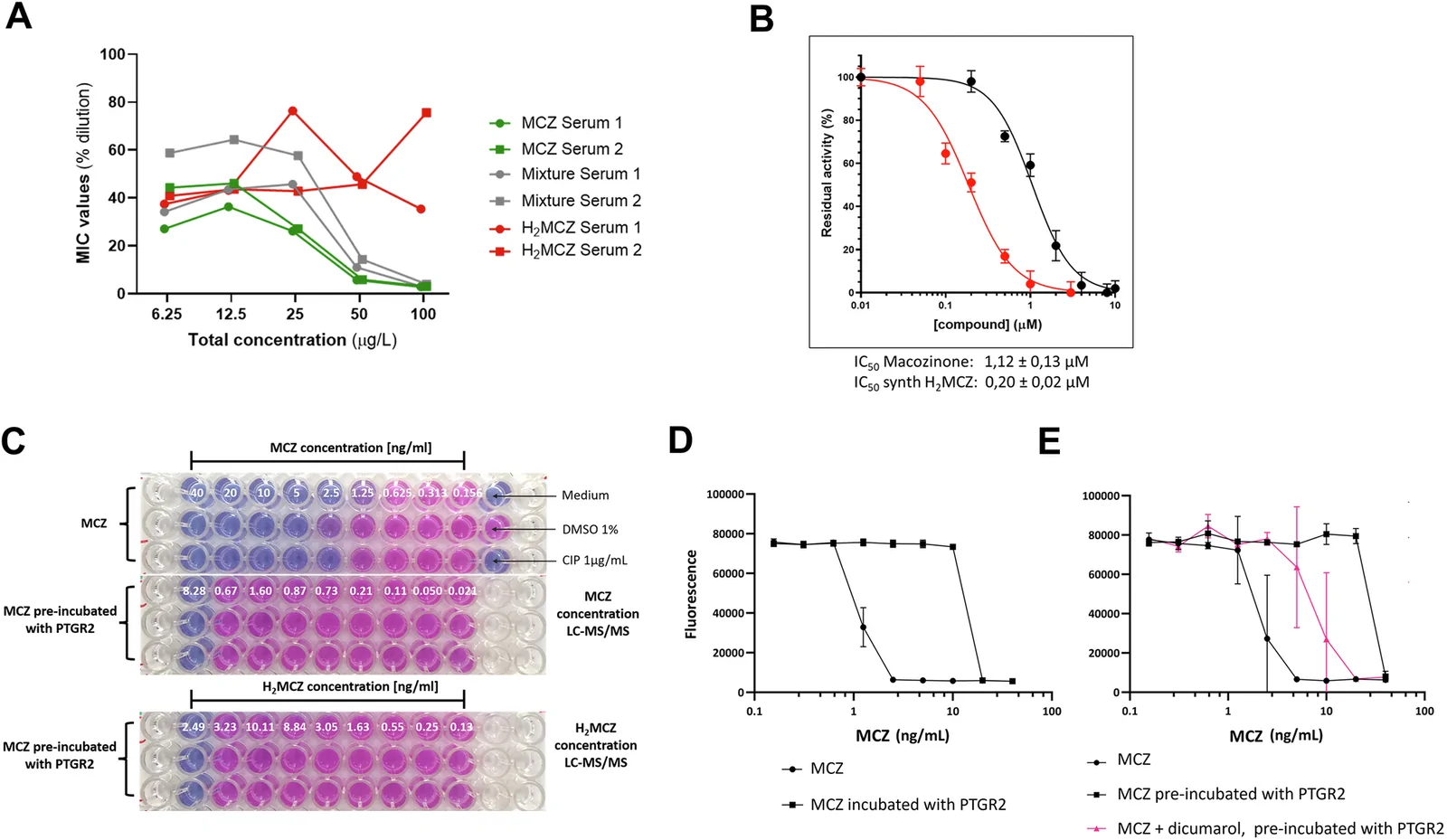
Functional consequences of macozinone dearomatization: in vitro. (**A**) Spike-in experiments. MIC values for human serum controls 1 and 2 spiked with MCZ (green), synthetic H_2_MCZ (red), or a mixture of the two (gray) at increasing concentrations. (**B**) In vitro DprE1 inhibition experiments. IC_50_ determination with pure enzyme. (**C**) Functional consequence of MCZ dearomatization. OD 0.0001; 72h-incubation, followed by 3 h incubation with resazurin. PTGR2 concentrations: 2.4 µM. Respective MCZ and H_2_MCZ concentrations are reported on the corresponding wells. (**D**) Reduced MCZ antimycobacterial activity follows enzymatic dearomatization. MCZ and H_2_MCZ concentrations were determined by LC-MS/MS in the PTGR2-preincubated MCZ; mean (*n* = 3) ± SD. (**E**) Decrease of macozinone antimycobacterial activity upon enzymatic dearomatization, mitigated by PTGR2 inhibition; mean (*n* = 3) ± SD. Source data are available online for this figure.

However, in human sera samples spiked with known concentrations of MCZ, H_2_MCZ, or a mixture of both, the lowest MICs were found for serum samples spiked with MCZ, followed by the MCZ/H_2_MCZ mixture, while the highest were for H_2_MCZ alone (Fig. 5A). Of note, MIC values for H_2_MCZ may be altered in part by H_2_MCZ spontaneously re-oxidizing into MCZ during the incubation process, in addition to the residual MCZ (evaluated at 7% by LC-MS/MS) present in the synthetic H_2_MCZ.

### Inhibition of DprE1

Then, we compared in a cell-free system the respective inhibition by MCZ and H_2_MCZ of the purified *M. tuberculosis* DprE1. Remarkably, in this in vitro test system, synthetic (*o-* and *p-*) H_2_MCZ was about 5-times more potent than MCZ itself (Fig. 5B).

### In vitro dearomatization of macozinone

The impact of MCZ reduction by PTGR2 on its killing activity against *M. smegmatis* is shown in Fig. 5C (96-well plate, *n* = 3) and Fig. 5D (concentration–activity profile). We used either MCZ (covering the relevant 10^–4^–10^2 ^ng/mL concentration range) (Fig. 5C, upper three lines), or MCZ preincubated with PTGR2 (Fig. 5C, central and lower lines).

MCZ and H_2_MCZ concentrations were determined by LC-MS/MS in the PTGR2-preincubated MCZ wells, and are reported above the respective wells. PTGR2-mediated dearomatization of MCZ induced a clear shift of the blue-pink border (i.e., dead/alive bacteria) to the left, indicative of a decrease in antimycobacterial activity (Fig. 5D), corresponding to an increase in MIC from 1 µg/L with pure MCZ to *ca* 18 µg/L. This decrease in antimycobacterial activity after PTGR2-mediated dearomatization could be partially mitigated by adding dicumarol during the pre-incubation step (Fig. 5E).

### Ex vivo observations

We also examined ex vivo the antimycobacterial activity of plasma samples obtained from a representative volunteer participating in a 14-day multiple ascending dose phase 1 clinical trial with MCZ native crystal powder (https://clinicaltrials.gov/study/NCT03776500). Direct incubation of these plasma samples with *M. smegmatis* for 72 h at 37 °C was unsuccessful, as blank plasma per se already prevented growth. Instead, we carried out prior plasma protein precipitation of the volunteer samples, followed by dilution according to concentration quantified by LC-MS/MS, to bring MIC levels within the 20–0.02 ng/mL working range of our colorimetric assay.

**Figure 6:**
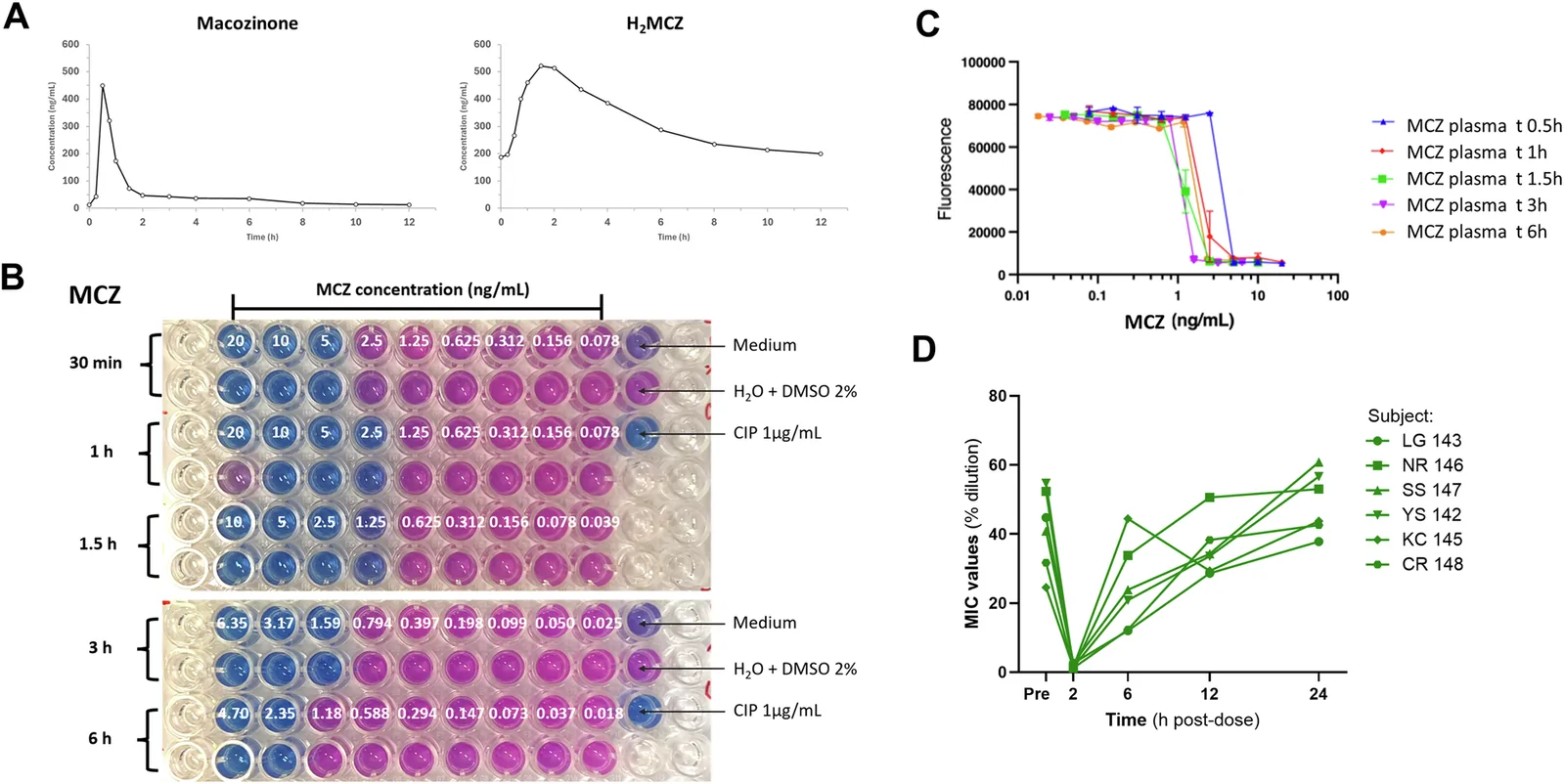
Functional consequences of macozinone dearomatization: ex vivo. (**A**) Pharmacokinetic profiles of macozinone and H_2_MCZ in plasma in one volunteer on the last day of the 14-day multiple-dose regimen. (**B**) Macozinone (MCZ) concentrations in wells. OD 0.0001 inoculum; 72 h-incubation, followed by 3 h incubation with resazurin. Serial dilutions of the volunteer’s samples on day 14 have been carried out according to plasma levels determined in the clinical pharmacokinetic study to bring the MCZ level in the working ~0.02 µg/L–20 µg/L range of the resazurin assay. (**C**) Antimycobacterial activity profile of plasma sample extracts from the same volunteer, plotted according to macozinone concentration; mean (*n* = 2) ± SD. (**D**) MIC values for six subjects in the single ascending PBTZ169 dose study 1a, at various time points after dosing. Source data are available online for this figure.

Figure 6A shows the PK profiles of MCZ and H_2_MCZ in the volunteer on the last day of the 14-day multiple ascending dose regimen. Figure 6B shows the corresponding MIC determination in the diluted extracts of plasma collected 0.5, 1, 1.5, 3, and 6 h after oral MCZ intake, and Fig. 6C shows the respective antimycobacterial activity profiles, plotted against MCZ levels.

While the MIC follows the MCZ concentration closely, with the blue-pink border observed around 1 µg/L (Fig. 6B,C), the H_2_MCZ concentration in the wells does not correlate with the antimycobacterial activity (Appendix Fig. S2).

We also examined ex vivo the antimycobacterial activity of serum samples obtained from six volunteers included in a randomized, double-blind, placebo-controlled, single ascending dose trial of MCZ (https://clinicaltrials.gov/study/NCT03423030). Figure 6D shows the MIC values of human serum collected at 0, 2, 6, 12, and 24 h after receiving 320 mg of MCZ (Spray-Dried Dispersion formulation). All 6 subjects showed low (1–2 ng/mL) MIC values against *M. smegmatis* at 2 h after dosing. For the pre-dose and all later serum samples, MIC values remained consistently high (35–70 ng/mL).

## Discussion

Since the identification in 2017 of the metabolism of benzothiazinones into Hydride–Meisenheimer complexes (HMC) in vivo, the enzyme responsible for this reaction had remained mysterious. While HMC formation had never been reported in mammals, including humans, it has been widely described in microorganisms. This prompted us to direct our early investigations towards the gut microbiome as the source of H_2_MCZ production, but this hypothesis was incorrect.

Here, we report that the in vivo bioreductive dearomatization of MCZ into H_2_MCZ is catalyzed principally by the NADPH-dependent prostaglandin reductase 2 (PTGR2), and, to a lesser extent, by prostaglandin reductase 1 (PTGR1). The slightly higher activity of PTGR2 than PTGR1 should be weighed against their differential tissue distribution and expression levels throughout the body (see e.g., The Human Protein Atlas (https://www.proteinatlas.org/). PTGR1 and PTGR2 are key mammalian enzymes involved in the inactivation of the endogenous substrate prostaglandin (PG) 15-keto-PGE2. The multifunctional PTGR1 is responsible for the irreversible degradation of various eicosanoids, such as prostaglandins, leucotrienes, and lipoxins, endogenous lipid mediators crucial in a wide range of physiological and pathological processes, such as inflammation and defense responses, notably upon *M. tuberculosis* infection (Ning et al, 2023).

This is the first report of the implication of PTGR2 in the metabolism of a xenobiotic compound. The reaction is blocked by dicumarol, by the specific inhibitor PTGR2-IN-1, and by the widely used NSAIDs diclofenac and indomethacin. The related homodimeric PTGR1 also contributes to MCZ reduction and is inhibited as well by diclofenac, indomethacin, and niflumic acid. In fact, while all double bonds of the aromatic ring of MCZ are conjugated, the aromatic double bond adjacent to the carbonyl group is peculiar in that it can be considered as a virtual α,β-unsaturated carbonyl moiety, owing to the presence of a strong electrophilic β carbon (Fig. 1A). Therefore, though not obviously apparent on considering their chemical structures, MCZ shares with prostaglandins E the same α,β-unsaturated ketone chemical motif, though this is virtual for MCZ, as the double bond is part of the aromatic ring (Fig. 2A), which likely explains why this xenobiotic is a substrate of enzymes catalyzing the reduction of 15-keto-PGE2 prostaglandins. Such a reaction is unique in the sense that it implies enzymatic β-reduction of a double bond that is engaged in an aromatic ring, to create the HMC.

Conceptually, our findings expand the chemical repertoire of reactions catalyzed by mammalian reductases and uncover a noncanonical enzymatic mechanism by which host metabolism can neutralize antimicrobial agents. Notably, in contrast to the numerous cytochrome P450s and reductases generally involved in drug metabolism, it is most unusual for enzymes involved in the regulation of endogenous mediators, such as prostaglandins, to be also implicated in the metabolism of xenobiotics.

The discovery of PTGR1/PTGR2-mediated MCZ metabolism casts light on a critical metabolic reaction undergone by antimycobacterial benzothiazinones. Liu et al hypothesized that H_2_MCZ may be the active derivative of MCZ. In our hands, H_2_MCZ showed higher activity against purified mycobacterial DprE1 than the parent compound MCZ itself. The slightly higher potency of H_2_MCZ against DprE1 is not unexpected. Examining the crystal structure of the DprE1-MCZ complex (Makarov et al, 2014) it is evident that the oxygen atom of the benzothiazinone core is positioned in front of Lys134, at a distance of ~3 Å. The presence of a negative charge on this oxygen atom in H_2_MCZ (see Fig. 1B) could lead to an ionic interaction with the lysine amine group, which would stabilize the compound in the active site of DprE1 and consequently increase the enzyme’s affinity for the inhibitor (Appendix Fig. S3). This could explain the lower IC₅₀ displayed by H₂MCZ. Also, it is very unlikely that H_2_MCZ is rapidly re-oxidized into MCZ by DprE1, as we would then expect to find a similar IC_50_ value for the two compounds. Moreover, DprE1 is known to have a slow kinetic rate, with a k_cat_ for the substrate ranging from 3 to 12 min^–1^ (depending on the electron acceptor). Also, the kinetics of BTZ activation is rather low, for example, the k_inact_ for BTZ043 is 0.6 min^–1^ (Neres et al, 2012). In this context, it is improbable that DprE1 can quickly oxidize H_2_MCZ.

Alternatively, in our in vitro MIC determinations, synthetic H_2_MCZ (*o*- and *p*-isomers mixture) was almost as potent as MCZ against *Mycobacterium* strains. Again, we cannot exclude that the latter results may be compromised to some extent by spontaneous H_2_MCZ re-oxidizing to MCZ during the incubation process, in addition to the residual MCZ in the synthetic H_2_MCZ.

Indeed, LC-MS/MS analyses of the *Mycobacterium*-free incubation reaction medium reveal that after 72 h at 37 °C, H_2_MCZ decreases by 93%, with a concomitant, yet not proportional, increase of MCZ (data not shown). Also, HMC species are commonly unstable intermediates; the electrophilic H_2_MCZ may react with some nucleophilic species present in the assay, which would explain the unbalanced stoichiometry of the H_2_MCZ oxidation reaction.

Overall, our data collectively indicate that the in vivo antimycobacterial activity is predominantly due to MCZ per se, rather than to its metabolite H_2_MCZ. This is supported by several preclinical experiments in mice that demonstrated strong anti-TB activity of MCZ. In mice, very low plasma levels of H_2_MCZ have been found (data not shown), in contrast to rats, dogs, and humans. Therefore, H_2_MCZ is unlikely to mediate any antimycobacterial activity in mice (Makarov et al, 2014).

Moreover, in our in vitro studies with MCZ pre-incubated with PTGRs before antimycobacterial tests, PTGR2-mediated reduction resulted in an overall decrease in activity (i.e., higher MIC). Likewise, the MIC values determined with serum samples spiked with MCZ, H_2_MCZ, or a mixture of both, or with ex vivo human plasma samples, follow the pharmacokinetic profile of MCZ, but not that of H_2_MCZ. Thus, we conclude that MCZ is not a prodrug and that H_2_MCZ, albeit circulating in abundance in humans, is not the active form. However, H_2_MCZ represents a potential drug reservoir thanks to its ability to reoxidize to MCZ, thus prolonging its pharmacokinetic half-life. This hypothesis remains unproven at this stage and requires testing by further drug tissue biodisposition studies.

Exploiting their original whole-cell assay, Kloss’s group has initiated optimization efforts to diminish the propensity of benzothiazinones to form HMC species, via the modulation of C-6 substituted nitro-benzothiazinone compounds, or by the replacement of the nitro group, but this substantially decreased antimycobacterial potency. Instead, our work on the key role of PTGR1/PTGR2 for the dearomatization of benzothiazinones suggests that suitable enzymatic inhibitors of the reduction reaction (e.g., NSAIDs) could impair MCZ reduction and improve its exposure in vivo. Indeed, the addition of the inhibitor dicumarol to the macozinone-PTGR2 incubation mixture restored, in part, the antimycobacterial activity. Such an approach to boost exposure in patients is achieved by combining CYP3A4 inhibitors (ritonavir or cobicistat) with antiretrovirals or using the dehydropeptidase inhibitor cilastatin with the antibiotic imipenem. However, a word of caution is due about inhibiting PTGR activity, since endogenous eicosanoids are important modulators of the inflammation and immune response to *M. tuberculosis* infection. Notably, PTGR1 inactivates the inflammatory mediator leukotriene LTB4, which restricts inflammation and confers resistance to tuberculous infection (Tobin et al, 2013).

Despite the unequivocal demonstration of the PTGR1/2-mediated dearomatization of MCZ, several limitations in our work have to be acknowledged. First, only the *p-*H_2_MCZ isomer is observed upon incubation of MCZ with PTGR1/2 enzymes, as also seen in human plasma samples (Fig. 2A). Yet, in our in vitro tests, synthetic H_2_MCZ (i.e., *o*- and *p*-H_2_MCZ) was used, of which the naturally occurring *p*-H_2_MCZ constitutes only 41% (determined by ^1^H-NMR) of the isomeric mixture (Appendix Fig. S4). Functional conclusions derived from the in vitro assays may thus not fully reflect the behavior of the physiologically relevant *p*-H_2_MCZ isomer. However, in Fig. 5C–E (incubation of MCZ with the pure enzyme PTGR2) and Fig. 6B–D (ex vivo incubation assays using samples from volunteers), only the naturally occurring *p*-H_2_MCZ is present. This observation suggests that these biologically relevant conditions, where only *p*-H_2_MCZ is formed, most likely reflect the in vivo situation. Of note, our efforts to separate the *o*- and *p*-H_2_MCZ by semi-preparative HPLC (using an adaptation of the method shown in Fig. 2A) were unsuccessful, as a substantial percentage was found to reoxidize to MCZ during the separation. In contrast, *p*-H_2_MCZ was found to be rather stable in volunteers’ plasma samples using appropriate precautions (Desfontaine et al, 2023).

Detailed pharmacokinetic aspects of the in vivo formation and elimination of H_2_MCZ remain to be described, ideally through a mass balance clinical trial. A feasibility study for boosting MCZ and BTZ-043 exposure by PTGR blockade is certainly warranted as it may allow the use of lower doses of the drugs but caution should be taken given the known immunomodulatory roles of prostaglandin metabolism.

Overall, the unprecedented PTGR-mediated dearomatization described here for macozinone also extends to BTZ-043, indicating that this biotransformation may be a general feature affecting the whole family of nitroaromatic benzothiazinones.

## Methods

**Table:** Reagents and tools table

Reagent or resource	Manufacturer (#ref)	Use/comment
Acetonitrile (ACN, C₂H₃N)	Merck (1000302500)	Mobile phase and needle wash solutions for LC-MS/MS, precipitation solution
Ammonium hydroxide (NH_4_OH)	Fluka (44273)	Mobile phase component for LC-MS/MS
Acetic acid (CH_3_COOH)	Sigma-Aldrich (5.33001)	Mobile phase component for LC-MS/MS
Formic acid (HCOOH)	Sigma-Aldrich (1.00264)	Mobile phase component for LC-MS/MS
H_2_O millipore	Merck (Mili-Q IQ7000)	Multiple uses
Methanol (MeOH, CH_3_OH)	Merck (1.06009.2500)	Needle wash and seal wash solutions for LC-MS/MS; solvent
Ethanol (EtOH, CH_3_CH_2_OH)	Sigma-Aldrich (1.00983)	Needle wash and seal wash solutions for LC-MS/MS; solvent
BCA assay kit	ThermoFisher Scientific (23227)	Determination of protein concentration
Sucrose (C_12_H_22_O_11_)	Sigma-Aldrich (84100)	Homogeneisation buffer for S9 fraction
Glycerin (C_3_H_8_O_3_)	Hänseler (07-3900-02)	Microsome storage buffer
Monopotassium phosphate (KH_2_PO_4_)	Sigma-Aldrich (P5655)	Potassium phosphate buffer
NADP+	Merck (10128031001)	NADPH-generating mix
Magnesium chloride hexahydrate (MgCl_2_ · 6H_2_O)	Hänseler (06-5232-01)	NADPH-generating mix
Glucose-6-phosphate (G6P)	Merck (10127647001)	NADPH-generating mix
Glucose-6-phosphate dehydrogenase (G6P-DH)	Merck (G6PDHII-RO / 10127671001)	NADPH regenerating mix (Malatkova buffer)
NADH	Merck (N8129)	Cofactor
Tris-HCl 1 M pH 8.5	Jena Bioscience (BU-124S-85)	Buffer preparation – adjusted to the required concentration and pH
Hydrochloric acid (HCl)	Merck (1003171000)	pH adjustment
Sodium hydroxide (NaOH)	Sigma-Aldrich (S5881)	pH adjustment
1xPBS buffer	CHUV. Laboratorium Dr. G. Bichsel AG (100 0 324)	Mitochondria wash (cold PBS)
Dimethyl-sulfoxide (DMSO, C_2_H_6_OS)	Sigma-Aldrich (D4540)	Solvent
Thioredoxin Reductase Colorimetric Assay Kit	Creative Biomart (0834)	Putative enzyme test
Thioredoxin Reductase Activity Assay Kit	Creative Biomart (0833)	Putative enzyme test
Human recombinant NQO1	Sigma-Aldrich (D1315)	Putative enzyme test
Human recombinant GR	Sigma-Aldrich (G9297)	Putative enzyme test
Human recombinant GSTP1	Sigma-Aldrich (GS-70)	Putative enzyme test
Human recombinant GLRX1	Creative Biomart (GLRX-139H)	Putative enzyme test
Auranofin (AF, C_20_H_34_AuO_9_PS)	Sigma-Aldrich (A6733)	Inhibition test
2-AAPA hydrate (C_18_H_22_N_4_O_6_S_4_)	Sigma-Aldrich (A4111)	Inhibition test
Cibacron Blue 3G-A (C_29_H_20_ClN_7_O_11_S_3_)	Sigma-Aldrich (B1064)	Inhibition test
Menadione (C_11_H_8_O_2_)	Sigma-Aldrich (M5625)	Inhibition test
Dicoumarol (C_19_H_12_O_6_)	Sigma-Aldrich (287897)	Inhibition test
Curcumin (C_21_H_20_O_6_)	–	Inhibition test
L-glutathione, oxidized (GSSG, C_20_H_32_N_6_O_12_S_2_)	Sigma-Aldrich (G4376)	Referenced substrate for GR
L-glutathione, reduced (GSH, C_10_H_17_N_3_O_6_S)	Sigma-Aldrich (G4251)	Putative mechanism test
Duroquinone (C_10_H_12_O_2_)	Sigma-Aldrich (D223204)	Referenced substrate for NQO1
Duroquinol (C_10_H_14_O_2_)	Combi-Blocks/Sigma-Aldrich (QF5349)	Product of duroquinone reduction by NQO1
Dehydroascorbate (DHA)	Sigma-Aldrich (261556)	Referenced substrate for GLRX1)
Ascorbic acid (AA)	Sigma-Aldrich (A4544)	Product of DHA reduction by GLRX1
1-chloro-2,4-dinitrobenzene (CDNB)	Sigma-Aldrich (237329)	Referenced substrate for GST(P1)
Glutathionyl-2,4-dinitrobenzene (GS-DNB)	Toronto Chemicals (D480150)	Product of GSH conjugation to CDNB by GST(P1)
N-ethylmaleimide (NEM)	Sigma-Aldrich (04259)	GSH depletion
Progesterone	Provided by the Laboratory of Clinical Chemistry (LCC), CHUV	Inhibition test
4-androstene-3,17-dione	Sigma-Aldrich (46033)	Inhibition test
Hydrocortisone	Golaz pharmacy, Lausanne	Inhibition test
Prednisone	Golaz pharmacy, Lausanne	Inhibition test
Diclofenac sodium	Golaz pharmacy, Lausanne	Inhibition test
Indomethacin	Golaz pharmacy, Lausanne	Inhibition test
Niflumic Acid	Sigma-Aldrich (4394-00-7)	Inhibition test
Albumin-dextrose-catalase (ADC)	Millipore (M0553)	MIC assays
Glycerol	Sigma-Aldrich (49769)	Multiple uses
L-ascorbic acid	Sigma-Aldrich (A4544)	Positive control GLRX1
Middlebrook 7H9 broth	Millipore (M0178)	Cultures
PTGR2-IN-1	MedChemExpress (HY-122716)	Inhibition test
Recombinant human PTGR1	Assay Genie (RPPB2222)	Putative enzyme test
Recombinant human PTGR2	Assay Genie (RPPB2223)	Putative enzyme test
Resazurin sodium salt	Sigma-Aldrich (R7017)	MIC assays
TWEEN® 80	Sigma-Aldrich (P1754)	Cultures
β-Lactoglobulin from bovine milk	Sigma-Aldrich (L3908)	Inhibition studies, enzyme incubations

### General

All incubation experiments with mammalian tissues have been performed at the Laboratory of Clinical Pharmacology of the University Hospital of Lausanne and the University of Lausanne, Switzerland. 1.5mL-Eppendorf Safe-Lock® Tubes were used for incubation experiments performed at 30 °C and 37 °C, using an Eppendorf ThermoMixer® equipped with the complementary heated lid Eppendorf ThermoTop®, except for the incubation experiments with cellular fractions derived from the pig tissues, which were performed using a temperature-controlled water bath. Incubation at room temperature corresponds to 25 °C.

Synthetic H_2_MCZ was prepared as follows: to a solution of 1.2 g MCZ (2.63 mmol) in 25 mL of acetonitrile with 0.5 mL of water, small batches of finely ground 0.2 g sodium borohydride (5.3 mmol) were added in small portions under argon with vigorous stirring at room temperature. A yellow-red precipitate forms after a few minutes. This solid was filtered and washed with a small amount of acetonitrile and diethyl ether and immediately vacuum dried. The yield is 0.8 g (66%), and the obtained product does not need recrystallization. The compound was stored in brown glass vials under argon at +4 °C. The synthetic mixture was determined to contain about 7–10% of residual MCZ by LC-MS/MS. The respective proportion of *o-* and *p-*H_2_MCZ in the synthetic mixture was determined by ^1^H-NMR (Appendix Fig. S4) using the ratio of the aromatic proton (d 7.16 and 7.31 ppm, on C5 and C7, respectively) and was found by ^1^H-NMR (Appendix Fig. S4) to contain 41% of naturally occurring *p*-H_2_MCZ and 59% of the *o*-isomer.

### Mass spectrometry methods

The LC-MS/MS analyses were performed using ultra-high-performance LC (UHPLC) instruments from ThermoFisher Scientific (San Jose, CA, USA) equipped with a binary pump, an autosampler with a flow-through needle, and a thermo-adjustable column compartment. TSQ Quantis and TSQ Altis MS instruments were coupled via diverter valve to Vanquish UHPLC systems. The ESI source was an Ion Max NG^TM^ electrospray ionization source equipped with a heated electrospray ionization (H-ESI) probe. HPLC vials containing sample extracts were stored at +4 °C in the temperature-controlled autosampler over the entire analytical sequence. Extracts samples were kept in snap ring/crimp neck HPLC glass vial and compatible snap ring closures from Macherey-Nagel.

For enzymatic kinetic experiments, the concentration of H_2_MCZ produced was calculated using matrix-matched calibration curves containing H_2_MCZ at concentrations of 2, 5, 10, 20, 50, 100, 300, 800, and 2000 ng/mL. Matrix-matched calibrators samples contained a solution of NADPH generating mix (20 mM NADP⁺, 15 mM glucose-6-phosphate, 24 mM MgCl₂, in Tris-HCl 100 mM pH 7.4) and potassium phosphate buffer (incl. dithioerythritol, EDTA) (ratio 3:5, v/v), 5% DMSO:MeOH (1:1, v/v). Test samples and calibration were subjected to the same precipitation step with ACN (sample:ACN 3:10) prior to centrifugation (10 min, 15,000 × *g*, +4 °C), and insertion of supernatant (ca. 45 µL) in HPLC vials. Of note, H_2_MCZ solutions are prone to degradation. It is therefore advised to prepare on the day of analysis, stock and working solutions of H_2_MCZ to add *ex tempore* into blank plasma matrix for the preparation of calibration samples.

Details of other LC-MS/MS methods and Operating Procedures used throughout this work are available in the Appendix Information.

### Tissue homogenization and subcellular fractions preparation

Pig organs, namely lung, liver, spleen, and intestine, have been obtained from a local butcher and immediately stored at –20 °C after sacrifice, followed by long-term storage at −80 °C. The organs were homogenized in an ice water bath in Tris-HCl 50 mM pH 7.5 buffer containing 250 mM sucrose, protease inhibitor mix (Thermo-Scientific), and EDTA (3 mL of homogenizing solution per 1 g tissue), using an Omni TH tissue homogenizer (Omni^®^).

A first centrifugation (10 min, 600 × *g*, +4 °C) of homogenized tissues allowed elimination of tissue debris pellets. The second centrifugation step (10 min, 15,000 × *g*, +4 °C) allowed the separation of mitochondria in the pellets, and supernatants constituting the S9 fractions. Ultracentrifugation (60 min, 100,000 × *g*, +4 °C) of the S9 fraction provides the cytosol (supernatant) and microsomes (pellets).

Mitochondria and microsome fractions were carefully washed using cold phosphate-buffered saline (PBS) to remove any remnant S9 or cytosol residues before storage at –80 °C in homogenizing buffer and microsome storage buffer, respectively. Three to four careful washing steps were necessary to obtain colorless supernatants.

Protein concentration in fractions was determined using the bicinchoninic acid (BCA) assay (ThermoFisher Scientific, San Jose, CA, USA) using bovine serum albumin (BSA) as a standard, with a working range of 25–2000 µg/mL according to the manufacturer’s instructions. The high protein contents of some crude samples have necessitated preliminary tests with sample dilutions prior to the BCA assay.

### H_2_MCZ generation in tissues and cellular fractions

Tissue fractions, NADPH buffer, and potassium phosphate buffer (containing 0.1 mM EDTA and 5 nM dithioerythritol (DTT)) were pre-incubated for 10 min at room temperature (RT) to allow incubation mix equilibration. At T_0_, MCZ (final concentration 5.01 µM) was added to the incubation mix and immediately transferred into an incubator at 37 °C. At 5-, 10-, 20-, 35-, and 50-min time-points, a 15 µL incubation aliquot is collected and precipitated with 50 µL cold acetonitrile (ACN) containing the stable isotopically labeled internal standards (ISTDs) MCZ_d11_ 80 ng/mL and H_2_MCZ-ISTD 40 ng/mL.

Precipitated samples are then centrifuged (10 min, 15,000 × *g*, +4 °C), and the supernatant (*ca*. 45 µL) is collected and analyzed by LC-MS/MS. A negative control sample containing no tissue is also tested in parallel. The final DMSO content in the incubation assay is 0.23%.

### H_2_MCZ production in pig cytosol or hemolyzed human blood in the presence of NAD(P)H

The cytosols from the four different pig tissues, namely liver, lung, spleen, and intestine, are diluted to reach a 2 mg/mL protein content in homogenization buffer. The pig cytosols, as well as non-diluted whole hemolyzed human blood (15 mL), are pre-incubated with either i) 6 mL NADPH 20 mM buffer, or ii) NADH 20 mM buffer, or iii) only Tris-HCl 100 mM, in the presence of 10 mL potassium phosphate buffer (incl. EDTA, DTT) for 30 min at room temperature. The subsequent incubation procedure was the same as described above (final MCZ concentration 3.17 µM). A negative control sample (no cytosol) was also tested to ensure that H_2_MCZ was not formed spontaneously in the absence of cytosols. The final DMSO concentration in the assay was 0.12%. Incubation mixtures were extracted after 15 and 30 min and subjected to LC-MS/MS analyses.

### H_2_MCZ production in human cytosol and S9 fractions

Post-mortem human tissues were kindly provided by the NIH NeuroBioBank (Request #1628). Liver, lung, and ileum post-mortem specimens were obtained from a single 21-year-old donor (ID 695). The specimens were stored at –80 °C. Sample preparation (tissue homogenization and S9 and cytosol isolation) was performed in our lab using the same procedure applied to pig tissues. Incubation experiments with MCZ followed the same procedure applied to pig samples as previously described.

### Pig liver cytosol enzymatic kinetics

The kinetics of the enzymatic reduction of MCZ with 20 mM NADPH were studied in PLC (adjusted to a protein concentration of 2 mg/mL), using the above-described incubation procedure and by varying MCZ concentrations from 0 µM to 30 µM. The maximum velocity V_max_ of the enzyme, as well as the apparent Michaelis constant K_M_, are determined from the Lineweaver-Burk plot of the reaction, calculated using Microsoft Office Excel 2016 and plotted with GraphPad Prism 9 (GraphPad Software, San Diego, CA, USA). Similar experiments were also performed by varying cytosolic pig liver protein concentration from 0 mg/mL to 20 mg/mL at a fixed 25 µM MCZ concentration in the medium (i.e., saturating).

### Enzymatic pathway screening assays

Macozinone dearomatization inhibition experiments were performed by incubation of MCZ in PLC in the presence of prototypical inhibitors of various putative reductive enzymatic pathways.

The experimental procedures for the screening of the various enzymatic pathways were as follows. Briefly, PLC extracts (2 mg/mL protein) were pre-incubated for 30 min at room temperature with NADPH buffer (6 µL) and 10 µL of potassium phosphate buffer containing EDTA and DTT, for initial incubation medium equilibration. The tested inhibitor (or blank solvent as positive control) was then added, and the mix was equilibrated for another 10 min at room temperature. Addition of MCZ (4 µl of 27.7 µM solution, 1.24% DMSO) into the incubation medium (final volume: 40 µL) and immediate transfer of the samples to an incubator at 37 °C corresponds to T0. The evolution of MCZ reduction in the incubation medium was followed at 5, 10, 20, 35, and 50 min.

Stock solutions of curcumin, dicumarol, 2-AAPA, auranofin, Cibacron Blue, progesterone, 4-androstene-3,17-dione, hydrocortisone, prednisone, diclofenac, and indomethacin were prepared in 100% DMSO; the stock solution of menadione was prepared in 100% ethanol (EtOH). All stock solutions were diluted tenfold in NADPH buffer and potassium phosphate buffer (incl. DTT, EDTA) (ratio 3:5, v/v), to yield working solutions containing 10% DMSO or EtOH (depending on the stock solution). The final organic solvent content in the assay was 1.37%.

The same procedure was applied for the establishment of Lineweaver-Burk plots of dicumarol and curcumin, which were determined at four MCZ final concentrations (0.27 µM, 0.53 µM, 1.07 µM, and 2.13 µM) in the presence of the inhibitors at concentrations covering their linear range of inhibition (Curcumin: 11.16 µM and 22.33 µM; Dicumarol: 0.083 µM and 0.166 µM). MCZ incubation experiments without an inhibitor were used as a positive control (100% yield).

MCZ incubation experiments in PLC were performed in the presence of various levels of the respective tested inhibitor, allowing the determination of the concentration that decreased H_2_MCZ production by 50% (IC50), calculated and plotted using the script provided in GraphPad Prism 9.

### Macozinone incubation experiment with recombinant enzymes

Metabolic pathway inhibition studies (Appendix Table S1) have oriented our investigation to several putative enzymes that have been successively tested alone or together for their ability to reduce in vitro MCZ to H_2_MCZ. The catalytic activity of the respective recombinant enzymes under study was ascertained with prototypical substrates.

#### Carbonyl-reducing enzymes from SDR and AKR superfamilies

Human recombinant CREs (CBR1, AKR1A1, 1B1, 1B10, and 1C3) were produced by an Escherichia coli expression system as per the methods described previously. To screen the CREs effect on MCZ, recombinant enzymes CBR1 (2 µg), AKR1A1 (2 µg), AKR1B1 (5 µg), AKR1B10 (5 µg), and AKR1C3 (3 µg) were incubated with 0.1 M sodium phosphate buffer (pH 7.4), regeneration system (final concentration in 100 µl of reaction mixture: 2.478 mM NADP, 17.21 mM glucose-6-phosphate, 9.756 mM MgCl_2_, 0.875 units of glucose-6-phosphate-dehydrogenase), and MCZ (4 µg/ml). The incubations were 30 or 60 min and they were stopped by adding 30 µl of reaction mixture into 90 µl of ice-cold ACN. After a quick vortex, the samples were centrifuged (15,000 × *g*, 10 min, 4 °C) and supernatants (100 µl) were collected in a new labeled Eppendorf tube and immediately frozen to –80 °C. H_2_MCZ metabolite detection was performed by using the LC-MS/MS method described previously.

#### Human recombinant DT-diaphorase (NQO1) and glutathione reductase (GR)

Lyophilized human recombinant DT-diaphorase (NQO1) was reconstituted in Tris-HCl 25 mM, pH 7.4, to obtain a protein concentration of 1 mg/mL. Glutathione reductase (GR) was supplied in a buffered solution containing Tris-HCl 25 mM at pH 7.4, 1 mM EDTA, and 50% (v/v) glycerol. The NADPH regeneration system was prepared as per Malatkova et al (Malatkova et al, 2014).

Reduced L-glutathione (GSH) and oxidized L-glutathione (GSSG) were also added to the reaction mix when indicated, at a molar ratio of 20:1 (to reflect physiological concentration), yielding final concentrations of 813 µM and 40.1 µM for GSH and GSSG, respectively.

Functional activities of NQO1 and GR were verified by incubating their model substrates, duroquinone and GSSG, respectively, and by monitoring the corresponding reduced products, duroquinol (LC-MS) and GSH (LC-MS/MS), respectively. Incubations were carried out at 30 °C to avoid recombinant enzymes denaturation. The temperature of 30 °C was a suitable compromise between RT, the temperature at which enzyme-specific activity is declared by manufacturers, and 37 °C, the usual temperature in our MCZ PLC ex vivo incubations. In total, 15 µl-aliquots of the reaction mix were collected after 15 and 35 min, subjected to protein precipitation with ice-cold ACN (containing MCZ-d11 and H_2_MCZ-IS), and centrifuged. Supernatants were injected directly (NQO1 test) or after tenfold dilution with purified H_2_O (GR test) into LC-MS/MS instruments.

#### Thioredoxine reductase (TrxR) enzymatic tests

Commercial kits from Creative Biomart Inc., USA (ref. 0833 and 0834) were used according to the manufacturer’s protocol and adapted for the intended purpose. Per kit protocol, the NADPH-dependent reduction of the typical substrate 5,5-dithio-bis-(2-nitrobenzoic) acid (DTNB) to TNB by TrxR1 purified from rat liver is used as a positive control reaction. In our experiments, MCZ was used in parallel instead of DTNB, and the production of H_2_MCZ was monitored by LC-MS/MS. Besides, the specific gold-containing TrxR1 inhibitors aurothiomalate (ATM) (Omata et al, 2006) or auranofin (AF) (active within the nM range) (Gandin et al, 2010), were used for our own inhibition studies of MCZ PLC incubation.

In addition to the standard spectrophotometric analyses of DTNB and TNB recommended in the TrxR kit, samples were also analyzed by LC-MS/MS following our usual precipitation and supernatant collection steps. A control well with PLC instead of the enzymatic mix was used as a positive control for H_2_MCZ production in the kit’s reagents.

#### Putative glutathionylation and de-glutathionylation reactions of macozinone

For our investigations on the glutathionylation/de-glutathionylation hypothesis conceivably leading to H_2_MCZ, we examined several PLC incubation extracts for the presence of the postulated glutathionyl-macozinone (MCZ-SG) adduct, first by LC-MS/MS over the range of *m/z* = 300–800 using the Single Ion Monitoring (SIM) mode, and then by Orbitrap High Resolution Mass Spectrometry in positive and negative ionization mode, for the putative theoretical molecular weight *m/z* 763.80 for C_30_H_40_F_3_N_7_O_9_S_2_.

In an initial series of experiments with PLC, the compound 2-AAPA, an inhibitor of glutaredoxin 1 (GLRX1) that possesses de-glutathionylation activity, was also added at an IC100 concentration to block the putative secondary step of MCZ-SG de-glutathionylation. Alternatively, dicumarol, besides being the archetypal NQO1 inhibitor, can also block glutathione-S-transferase. We have therefore examined whether the strong inhibition of H_2_MCZ production by dicumarol observed in our PLC screening test system may be explained by a modulation of the hypothetical first step intermediate MCZ-SG conjugate. Incubation extracts were analyzed by LC-MS for the postulated glutathionyl-macozinone (MCZ-SG) adduct either directly or after a tenfold concentration step via evaporation under nitrogen and reconstitution of dried extracts in NADPH buffer containing 10% DMSO.

#### Recombinant glutathione-S-transferase P1 (GSTP1), glutaredoxin 1 (GLRX1) and glutathione reductase (GR)

Human recombinant glutathione-S-transferase P1 (GSTP1), supplied in buffered solutions, glutaredoxin 1 (GLRX1), provided with unstated enzymatic activity information, and glutathione reductase (GR) have been tested individually and conjointly, in the presence of GSH, for their potential ability to reduce MCZ to H_2_MCZ. An additional incubation test using NQO1 together with GSTP1 was also performed.

### Incubation studies with recombinant enzymes PTGR1 and PTGR2

MCZ at a final concentration of 11.41 µM was incubated at 30 °C for 15 min with 0.1 mg/mL recombinant human PTGR1 or PTGR2, in the presence of NADPH regenerating mix. Importantly, the incubation mixture also contained 6.25% EtOH to maintain MCZ solubility. Incubation mixtures were subjected to standard extraction with ACN solution containing ISTDs, and the supernatants were analyzed by LC-MS/MS through monitoring the respective H_2_MCZ and MCZ *m/z* transitions. Both analyses were performed in duplicate.

### In vitro enzymatic inhibition studies with recombinant PTGR1 and PTGR2

Recombinant human PTGR1 (3 µL, final concentration 1000 nM, 36 µg/mL) or PTGR2 (2 µL, final concentration 250 nM, 10 µg/mL) were incubated with NADPH regenerating mix (8.5 µL for PTGR1 and 9.5 µL for PTGR2), β-lactoglobulin (4 µL, final concentration 0.5 mg/mL) and inhibitors (dicumarol, indomethacin, diclofenac, or the PTGR2-specific inhibitor PTGR2-IN-1; 2 µL at different concentrations) for 10 min at room temperature (RT). As a control, the inhibitor was replaced by an equivalent volume of DMSO (inhibitor solvent). MCZ (2.5 µL, final concentration 12.5 µM) was then added, and the incubation mixtures were incubated for 60 min at 37 °C. To ensure MCZ solubility, the final incubation mixture contained 6.25% ethanol (v/v). After incubation, 15 µL of the incubation mixture was precipitated on ice with 50 µL cold ACN containing the internal standards MCZd_11_ (isotopically labeled, 80 ng/mL) and H_2_MCZ-ISTD (40 ng/mL). Samples were vortexed and then centrifuged (10 min, 17,130× *g*, 4 °C), and the supernatant (40 µL) was diluted with Milli-Q water (2:1, v/v) and analyzed by LC-MS/MS, monitoring the respective H_2_MCZ and MCZ *m/z* transitions (the LC-MS/MS method is described in the Appendix Information).

Enzymatic activity was normalized to the condition without inhibitor, which was defined as 100% activity, and relative activities were expressed as a percentage of this control. Half maximal inhibitory concentration (IC_50_) values were determined by non-linear regression (dose-response inhibition model) implemented in GraphPad Prism 9, and error bars on graphs represent the standard deviation from the mean of technical duplicates.

### DprE1 inhibition assay (in Fig. 5B)

*M. tuberculosis* DprE1 was prepared as previously reported. Enzyme activity assays were performed at 30 °C in a final volume of 100 μL, using an Amplex Red/peroxidase coupled assay slightly modified from Neres et al (Neres et al, 2012). The standard reaction mixture contained 50 mM glycylglycine at pH 8.5, 100 mM KCl, 0.2 μM horseradish peroxidase, 50 μM Amplex Red, 0.5 μM DprE1. The reaction was started by the addition of farnesyl-phosphoryl-*β*-_D_-ribofuranose (FPR) 400 μM, and the conversion of Amplex Red to resorufin was followed by measuring the increase in absorbance at 600 nm (ε = 54000 M^–1^ cm^–1^) on an Eppendorf BioSpectrometer.

For IC_50_ determination the enzyme activity assays were performed in the presence of different concentrations of inhibitors (0.05–10 μM), and values were determined by fitting data according to the following equation, using GraphPad Prism 8 software:

$${A}_{[I]}={A}_{[0]}\times \left(1-\frac{[I]}{\left[I\right]+{{IC}}_{50}}\right)$$

where A_[I]_ is the activity of the enzyme at inhibitor concentration [I] and A_[0]_ is the activity of the enzyme in the absence of inhibitor.

### In vitro dearomatization of MCZ (Fig. 5C–E)

Recombinant human PTGR2 (2 µL, final concentration 2433 nM, 100 µg/mL) was incubated with NADPH regenerating mix (9.5 µL) and β-lactoglobulin (4 µL, final concentration 0.5 mg/mL), with or without inhibitors (dicumarol or 25 mM Tris-HCl buffer as negative control, 2 µL) for 10 min at RT. MCZ (2.5 µL, final concentrations ranging from 4.0 to 15.6 ng/mL) was then added, and mixtures were incubated for 60 min at 37 °C. To ensure MCZ solubility, the final incubation mixture contained 6.25% ethanol (v/v). After incubation, 65 µL of cold ACN containing the internal standards MCZd_11_ (isotopically labeled, 80 ng/mL) and H_2_MCZ-ISTD (40 ng/mL), was added to the incubation mixture. Samples were vortexed thoroughly, and 64 µL was transferred to low-bind Eppendorf tubes. Samples were evaporated under nitrogen and reconstituted in 15 µL of dimethyl sulfoxide (DMSO), followed by 3 min sonication.

These pre-incubated samples were subsequently used for antimycobacterial activity testing, as described above, and the concentrations of MCZ and H_2_MCZ were determined by LC-MS/MS (described in the Appendix Supplementary Methods).

### Antimycobacterial assays

The experiments shown in Fig. 5C–E were carried out as follows. *M. smegmatis* mc^2^155 (ATCC 700084) was cultured in Middlebrook 7H9 broth supplemented with 0.4% glycerol, 0.04% TWEEN® 80, and 10% albumin-dextrose-catalase (ADC) at 37 °C with shaking. Fresh cultures were prepared from frozen stocks (–80 °C) and maintained at an optical density (OD_600_) of 0.5 to ensure consistent growth and minimize variability. The resazurin microtiter assay (REMA) was used to compare the antimicrobial activity of the sample against *M. smegmatis*. The inoculum was prepared from fresh culture and diluted in 7H9 broth to obtain an OD_6__00_ of 0.0001. For pre-incubated samples, 200 µL of inoculum was dispensed into sterile flat-bottom 96-well plates, followed by the addition of 2 µL of each pre-incubated sample. After mixing, 100 µL was removed from each well to normalize the final volume. For MCZ, a 4000 ng/mL stock solution was prepared in DMSO, and 2 µL was added to the first well containing 200 µL inoculum, followed by twofold serial dilutions across the plate. Each plate included a sterility control (7H9 broth), a growth control (inoculum with 1% DMSO), and a positive control (ciprofloxacin, 1 µg/mL). Sterile water was added to the perimeter wells to prevent evaporation. Plates were covered with a plastic membrane and incubated at 37 °C for 72 h under aerobic conditions. Resazurin (10 µL of 0.025% w/v stock solution) was added, and after 3 h of incubation at 37 °C, fluorescence of the reduced metabolite resorufin was measured (excitation 560 nm, emission 590 nm) using a FLUOstar Omega microplate reader (BMG LABTECH, Ortenberg, Germany). Data were analyzed using GraphPad Prism 9, and error bars on graphs represent the standard deviation from the mean of technical triplicate.

The experiments shown in Appendix Fig. S1A were carried out as follows. The minimum inhibitory concentrations (MICs) of MCZ, H_2_MCZ, and rifampicin (RIF) were determined against *M. tuberculosis* H37Rv and *M. smegmatis* MC²155 using the resazurin microtiter assay (REMA) adapted from Palomino et al (Palomino et al, 2002).

DMSO, glycerol, resazurin sodium salt, and rifampicin were obtained from Sigma-Aldrich (USA). Middlebrook 7H9 broth was purchased from Difco (USA), and albumin dextrose catalase (ADC) supplement from Chemie Brunschwig AG (Switzerland). The reference strain *M. smegmatis* MC²155 has been purchased from ATCC (ATCC 700084). The *M. tuberculosis* H37Rv strain has been provided by Prof. John McKinney at EPFL (and was originally from the Institut Pasteur in Paris).

Stock solutions of each test compound (10 mg/mL in DMSO) were prepared, and twofold serial dilutions were performed in sterile 96-well flat-bottom plates. Frozen aliquots of replicating bacilli were thawed, diluted to an OD_600_ of 0.0001 ( ≈ 3 × 10⁴ CFU/mL), and added to the wells to obtain a final volume of 100 μL. Plates were incubated at 37 °C for 6 days. Resazurin was then added (0.025% w/v, 1/10 of the well volume), and fluorescence of the reduced metabolite resorufin was measured after overnight incubation (excitation: 560 nm; emission: 590 nm) using a TECAN Infinite M200 microplate reader. The MIC was defined as the lowest drug concentration that prevented a visible color shift of resazurin from blue to pink and was confirmed quantitatively by fluorescence measurements. MIC values were calculated using GraphPad Prism 7. All compounds were tested in triplicate within a single independent experiment.

For the assessment of cross-resistance with MCZ and H_2_MCZ on BTZ-R *M. smegmatis* (Appendix Fig. S1B), the strains *M. smegmatis* mc^2^155 WT and MN84 (BTZ-R, bearing a C394S mutation in DprE1) were cultured in 7H9 supplemented with 10% OADC, 0.2% glycerol, and 0.05% Tween-80. Bacteria were diluted to an OD_600_ of 0.0001, dispensed in 96-well plates, and the drug was added and serially diluted. The bacteria were exposed to the drug for 24 h at 37 °C before 10% resazurin (0.0025%) was added and further incubated at 37 °C before measuring the fluorescence with a plate reader.

### Macrophage infection experiments (Appendix Fig. S1C)

RAW264.7 macrophages were grown in RPMI medium supplemented with 10% FBS at 37 °C under 5% CO_2_. Cells were allowed to adhere for 3 h, then infected with washed logarithmic phase *M. tuberculosis* H37Rv cells at an MOI of 10, then, after 5 h, extracellular bacteria were removed by washing cells twice before adding fresh RPMI medium containing the compounds to be tested. Plates were sealed and incubated at 37 °C in 5% CO_2_. After 72 h, the plates were left at room temperature (RT) for 1 h, then 5 µl of Prestoblue cell viability reagent (Life Technologies) were added, and after 1 h fluorescence was measured in a Tecan infinite M200 plate reader (excitation 570 nm, emission 590 nm).

### Spike-in serum samples MIC determination study (Fig. 5A)

Series of blank control human serum from two different donors were spiked (i) with MCZ to target concentrations of 100, 50, 25, 12.5 and 6.25 μg/L, (ii) with synthetic H_2_MCZ (*o*- and *p*-H_2_MCZ) to target concentrations of 100, 50, 25, 12.5 and 6.25 μg/L (e.g., used as calibrator during LC-MS/MS quantification of clinical study samples), (iii) with a 50%-50% mixture of MCZ and *o*-/*p*-H_2_MCZ at the same total concentrations as above. The actual concentrations of spiked samples were verified with the LC-MS method used for PK measurements (Desfontaine et al, 2023).

### Ex vivo MIC determination studies (Fig. 6A–D)

The experiments shown in Fig. 6A–C were carried out as follows. Plasma samples (collected on EDTA) were obtained from one volunteer included in a Phase Ib randomized, placebo-controlled, multiple ascending doses clinical trial (study name: IM-006–13, ClinicalTrials.gov identifier: NCT03776500) according to a protocol approved by the Institutional Ethics Committee. The study IM-006–13 has included 40 male volunteers (30 receiving the active treatment and 10 receiving a placebo), aged between 20 and 45, including 23 Caucasian, 14 African-descendant, 2 Hispanic/South American, and 1 multiracial participants. Briefly, 30 healthy volunteers received various doses of MCZ.HCl native crystal powder (NCP) ranging from 300 to 600 mg/day for 14 days, to assess the safety and tolerability of MCZ. Plasma (and urine) samples were collected at selected time points after MCZ intake on days 1 and 14 to study the pharmacokinetic/antimycobacterial profile of NCP-formulated MCZ in healthy volunteers.

Plasma samples from the volunteer on day 14 were analyzed to assess their antimicrobial activity against *M. smegmatis*. In total, 100 µL-aliquot of plasma was precipitated with 300 µL of cold ACN. Samples were vortexed thoroughly and centrifuged (10 min, 17,130 × *g*, +4 °C). A 300 μL supernatant aliquot was transferred to low-bind Eppendorf tubes. Samples were evaporated to dryness under nitrogen and reconstituted in Milli-Q water containing 8% (v/v) DMSO to obtain the desired concentration of MCZ and H_2_MCZ, followed by sonication for 3 min. These samples were used for the REMA assay.

The REMA assay was performed as previously described, with the following modifications. For each plasma sample, 150 µL of inoculum (OD_600_ = 0.00013) was dispensed into the first wells of sterile flat-bottom 96-well plates, and 100 µL of inoculum (OD_600_ of 0.00010) was added to the following wells. In total, 50 µL of each plasma sample was added to the first wells, mixed, and twofold serial dilutions were performed across the plate. Each plate included a sterility control (7H9 broth only), a growth control (inoculum with Milli-Q water containing 8% DMSO), and a positive control (ciprofloxacin at 1 µg/mL). This experiment was performed in duplicate.

The ex vivo experiments shown in Fig. 6D were carried out as follows. Briefly, serum samples were obtained from six volunteers included in a randomized, double-blind, placebo-controlled, single ascending dose trial to evaluate the safety, tolerability, pharmacokinetics, and ex vivo antitubercular activity of PBTZ169 Spray-Dried Formulation (study name: IM-006-11, ClinicalTrials.gov identifier NCT03423030) according to a protocol approved by the Institutional Ethics Committee. The randomized study IM-006-11 has included 32 male volunteers, aged between 19 and 43 (median 25.5 years; mean 27 ± 5.6 years), including 22 Caucasian, 8 African-descendant, 1 Hispanic, and 1 multiracial participant.

DMSO and sterile filtered human serum (male plasma AB) were obtained from Sigma-Aldrich (USA). Alamar blue solution was obtained from Thermo Scientific Oxoid (USA). Native crystalline powder of MCZ (PBTZ169.HCl) was obtained from Aptuit (UK) for use as a positive control, and the stock was prepared in DMSO. Middlebrook 7H9 culture medium and albumin dextrose catalase (ADC) culture medium supplement were purchased from Becton Dickinson (USA). For the study, sterile culture medium was prepared by mixing 1.88 g of Middlebrook 7H9 powder, 356 mL of ddH_2_0, 4 mL of 5% Tween-80 solution (final concentration 0.05%), 1.6 mL 50% glycerol solution (final concentration 0.2%) and 40 mL ADC (final concentration 10%) on a magnetic stirrer till homogeneous, followed by filtration using a 0.22-μm Filtermax vacuum filter from TPP (Switzerland, 99500). The media was stored at 4 °C and kept no more than one month at refrigerated conditions.

On the day of the experiment, a multiple of 10 frozen samples (corresponding to all serum samples of 1 clinical dose in 1 subject) were thawed at room temperature. A MCZ master stock was prepared in DMSO at a concentration of 3 mg/mL followed by dilution in DMSO to a working stock of 10 μg/mL. This was used to spike commercial human serum at a final concentration of 40 ng/mL for use as controls during the microplate assay. Two microplates were used to test a complete set of 1 dose samples from one subject in duplicate. The microplate preparation setup is described in Appendix Fig. S5. The MIC was defined visually as the last concentration preventing resazurin turnover from blue to pink and confirmed by the level of fluorescence measured by the microplate reader. Briefly, the noise signal obtained in the blank control wells is subtracted from the fluorescence values of the test samples. The percentage of the mycobacterial viability in each well is proportionally compared to 100% growth in the negative control wells. MIC was determined by using GraphPad Prism 7 software using the “Gompertz equation for MIC determination”. The dilution value where MIC_99_ occurs and tables for *M. smegmatis* for each dose per subject were determined. Real MIC_99_ values can then be calculated based on the MCZ concentration in serum samples determined by LC-MS/MS.

Informed consent was obtained from all human subjects, and experiments conformed to the principles set out in the WMA Declaration of Helsinki and in the Department of Health and Human Services Belmont Report.

### Data analysis and software

The acquisition and processing of the LC-MS/MS data have been performed by XCalibur™ software from ThermoFisher Scientific. The data have then been analyzed and plotted using Microsoft Office Excel 2016 and GraphPad Prism versions 7, 8 or 9 as specified throughout the method section (GraphPad Software, San Diego, CA, USA). Error bars on the graphs represent the standard deviations from the means of replicates (*n* = 2, unless specified otherwise). The chemical structures of compounds are represented using Chem DrawPrime 15.1 (ChemAxon Ltd.). ChatGPT was used as a support tool to improve clarity and grammar. All content was reviewed and validated by the authors.

## Supplementary information

Appendix

Peer Review File

Source data Fig. 2

Source data Fig. 4

Source data Fig. 5

Source data Fig. 6

## Data Availability

This study includes no data deposited in external repositories. The source data of this paper are collected in the following database record: biostudies:S-SCDT-10_1038-S44321-026-00526-1.
