# Supplementary material for: Human prostaglandin reductases dearomatize and inactivate benzothiazinone antitubercular drugs
Source: EMBO Mol Med. 2026 Oct 7;18(11):4413–32. doi: 10.1038/s44321-026-00526-1 (PMC13748546; doi:10.1038/s44321-026-00526-1)
Supplement: Supplementary file 1 [file 44321_2026_526_MOESM1_ESM.pdf]

APPENDIX

Human prostaglandin reductases dearomatize and inactivate benzothiazinone antitubercular drugs

Table Of Contents

Appendix Table S1 Stepwise identification of macozinone reductases. Background's intensity means that the inhibitor is known to strongly, moderately or slightly inhibit the considered enzyme. (Cited references are provided either in the main text or in the reference list at the end of the appendix) Page 2

Appendix Figure S1 (**A**) *In vitro* antimycobacterial activity of MCZ and H_2MCZ against *M. tuberculosis* H37Rv and *M. smegmatis* mc^{2}155 (n= 3, mean ± SD). (**B**) *In vitro* antimycobacterial activity of MCZ and H_2MCZ against WT and BTZ^R *M. smegmatis* (n= 2, mean ± SD). (**C**) Activity of MCZ and H_2MCZ against *M. tuberculosis in* Macrophage Infection Experiments. Page 3

Appendix Figure S2 H_2MCZ concentrations in wells. Serial dilutions of one volunteer's samples on day-14 (details given in Figs. 6B and 6C) Page 4

Appendix Figure S3 Binding site of MCZ in the DprE1 crystal structure (PDB ID: 4NCR). Page 5

Appendix Figure S4 ^{1}H-NMR spectrum of synthetic mixture of ortho- and para-H_2MCZ Page 6

Appendix Figure S5 Microplate preparation set up for *ex vivo* serum samples MIC determination (in Fig. 6D) Page 7

Appendix Supplementary Methods Liquid-chromatography coupled to mass spectrometry methods (N° 1-6) Page 8

Appendix References References cited in Appendix Table S1 that are not included in the main text Page 16

Appendix Table S1: Stepwise identification of macozinone reductases. Background's intensity means that the inhibitor is known to strongly, moderately or slightly inhibit the considered enzyme. (Cited references are provided either in the main text or in the reference list at the end of the appendix)

INHIBITORS / ENZYMES	Dicumarol	Curcumin	Menadione (Vitamin K3)	2-AAPA [a]	Cibacron blue [b]	Epigallocatechine gallate (EGCG)	Quercetin	Sodium aurothiomalate (ATM) 20 µM	Auranofin 7.38 µM	Dithiobis 2 nitrobenzoic acid (DTNB)	Progesterone [c]	4-androstene-3,15 dione [c]	Hydrocortisone [c]	Prednisone [d]	Diclofenac (COX inhibitor)	Indomethacin (COX inhibitor)	niflumic acid (COX-2 selective)	PTGR2-IN-1 inhibitor
**Carbonyl reductases**																		
NAD(P)H:quinone oxidoreductase 1 (DT-diaphorase, NQO1)	(Asher *et al*, 2006)	(Tsvetkov *et al*, 2005)			(Prochaska, 1988)	(Pandey *et al*, 2020)	(Karakurt *et al*, 2015)											
Cytosolic carbonyl reductase (CBR1)																		
Aldo-keto reductases (AK 1A1, 1B1, 1B10, 1C3)																		
**Redox cellular homeostasis**																		
Thioredoxin reductase, cytosolic isoform (TrxR1)		(Fang *et al*, 2005)	(Zhang *et al*, 2020)	(de Souza *et al*, 2017)		(Wang *et al*, 2008)	(Lu *et al*, 2006)	(Omata *et al*, 2006)	(Gandin *et al*, 2010)									
Glutathione reductase (GR)		(Guller *et al*, 2021)	(Duthie & Grant, 1989)	(Seefeldt *et al*, 2009)			(Guller *et al*, 2021)		(Gandin *et al*, 2010)									
**Nucleophilic aromatic substitution**																		
Glutathione transferase (GST)	(Mays & Benson, 1992)	(Duthie & Grant, 1989; Hayeshi *et al*, 2007)	(Ahmad & Srivastava, 2008)	(Seefeldt *et al*, 2009)	(Ahmad & Srivastava, 2008; Ismail *et al*, 2021)													
Glutaredoxin (GLRX1)				(Sadhu *et al*, 2013)														
**α, β-unsaturated ketone reductases**																		
Pentaerythritol TetraNitrate Reductase PETNR (*microbial enzyme*)											(Barna *et al*, 2001)	(Barna *et al*, 2001)						
Prostaglandin reductase 1 (PTGR1, *PGR/LTB4*)	(Yu *et al*, 2013)									(Kitamura *et al*, 1996)					(Clish *et al*, 2001; Huang *et al*, 2014)	(Clish *et al*, 2001; Yu *et al*, 2013)	(Clish *et al*, 2001; Mesa *et al*, 2015)	
Prostaglandin reductase 2 (PTGR2)																(Wu *et al*, 2008)		(Parker *et al*, 2017)
**OBSERVATIONS IN THIS STUDY :**																		
IC_{50} in Pig liver cytosol* [µM]	0.107	12.8	15.5	30.6	1.1 to 16.6		+	-	-		~50 µM	~50 µM	+	+	16.2	23.6	16.3	0.52

[a] R,R-2-Acetylamino-3-[4-(2-acetylamino-2-carboxyethylsulfanylthiocarbonylamino)phenylthiocarbamoylsulfanyl]propionic acid hydrate, S, S-[1,4-Phenylenebis(iminocarbonothioyl)]bis[N-acetyl-L-Cysteine] hydrate; [b] 1-amino-4-[4-[[4-chloro-6-(2-sulfoanilino)-1,3,5-triazin-2-yl]amino]-3-sulfoanilino]-9,10-dioxoanthracene-2-sulfonic acid; [c] α,β-saturated carbonyl steroids; [d] α,β-unsaturated carbonyl steroid. * Inhibitory activity @ macozinone 4.38 µM.

A

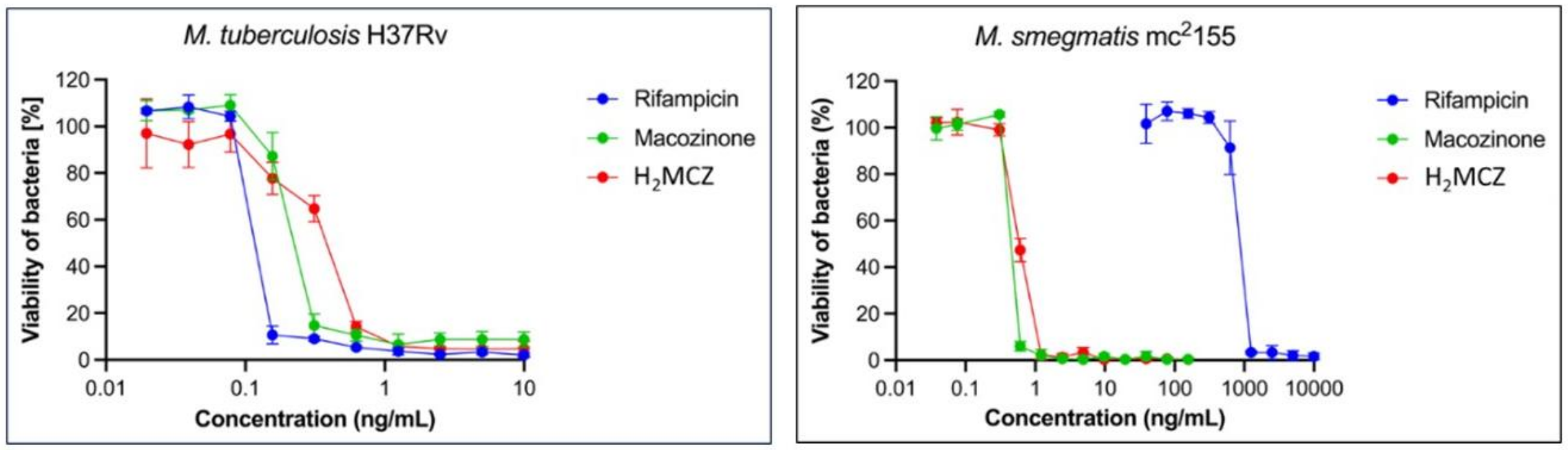

	MIC [µg/L]		
**Strains**	MCZ	H_2MCZ	Rifampicin
M. tuberculosis H37Rv	0.3	1.2	0.1
M. smegmatis mc2 155	0.6	1.2	1250

B

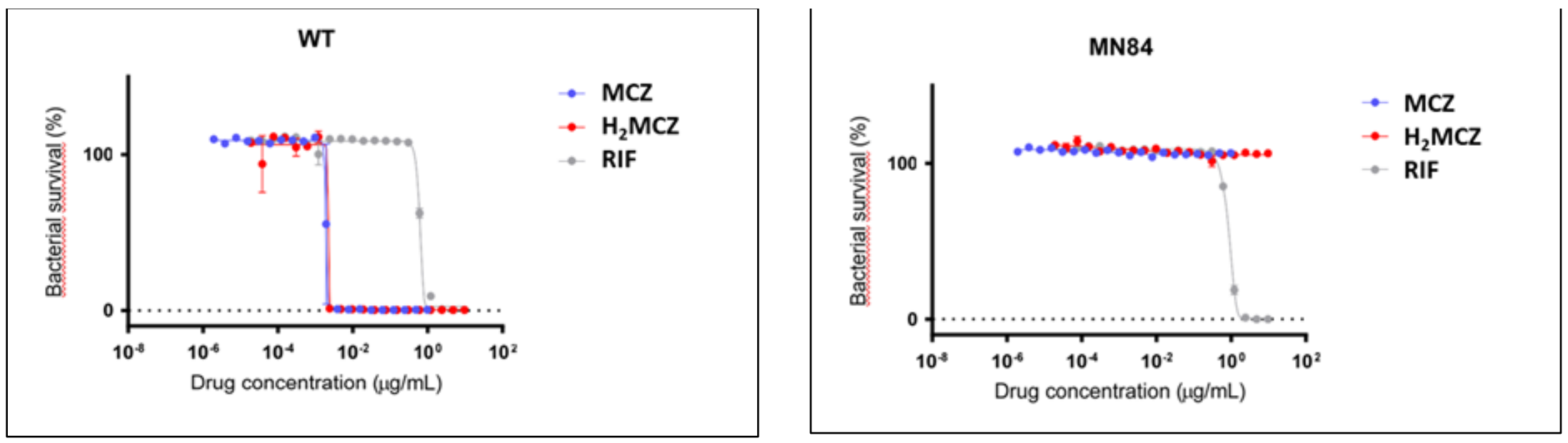

**Strains**	MCZ	H_2MCZ	Rifampicin
M. smegmatis wt	2	2	800
M. smegmatis MN84	> 1000	> 10'000	1400

C

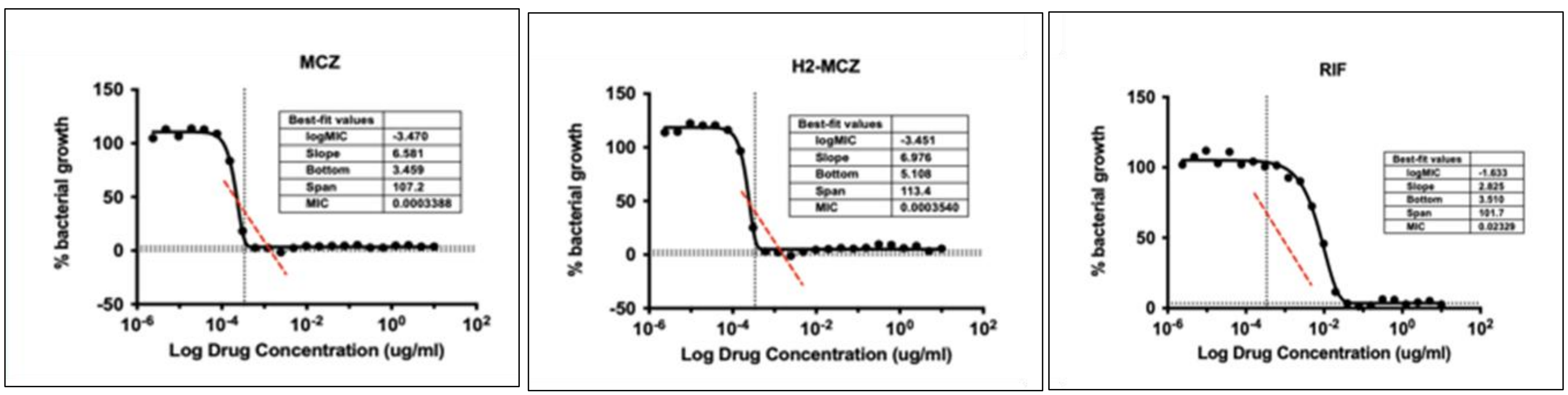

	MIC [µg/L]		
**Strains**	MCZ	H_2MCZ	Rifampicin
M. tuberculosis	0.3	0.3	23

Appendix Figure S1 (**A**) *In vitro* antimycobacterial activity of MCZ and H_2MCZ against *M. tuberculosis H37Rv* and *M. smegmatis* mc^{2}155 (n= 3, mean ± SD). (**B**) *In vitro* antimycobacterial activity of MCZ and H_2MCZ against WT and BTZ-R (MN84) *M. smegmatis* (n= 2, mean ± SD)**.** (**C**) Activity of MCZ and H_2MCZ against *M. tuberculosis in* Macrophage Infection Experiments.

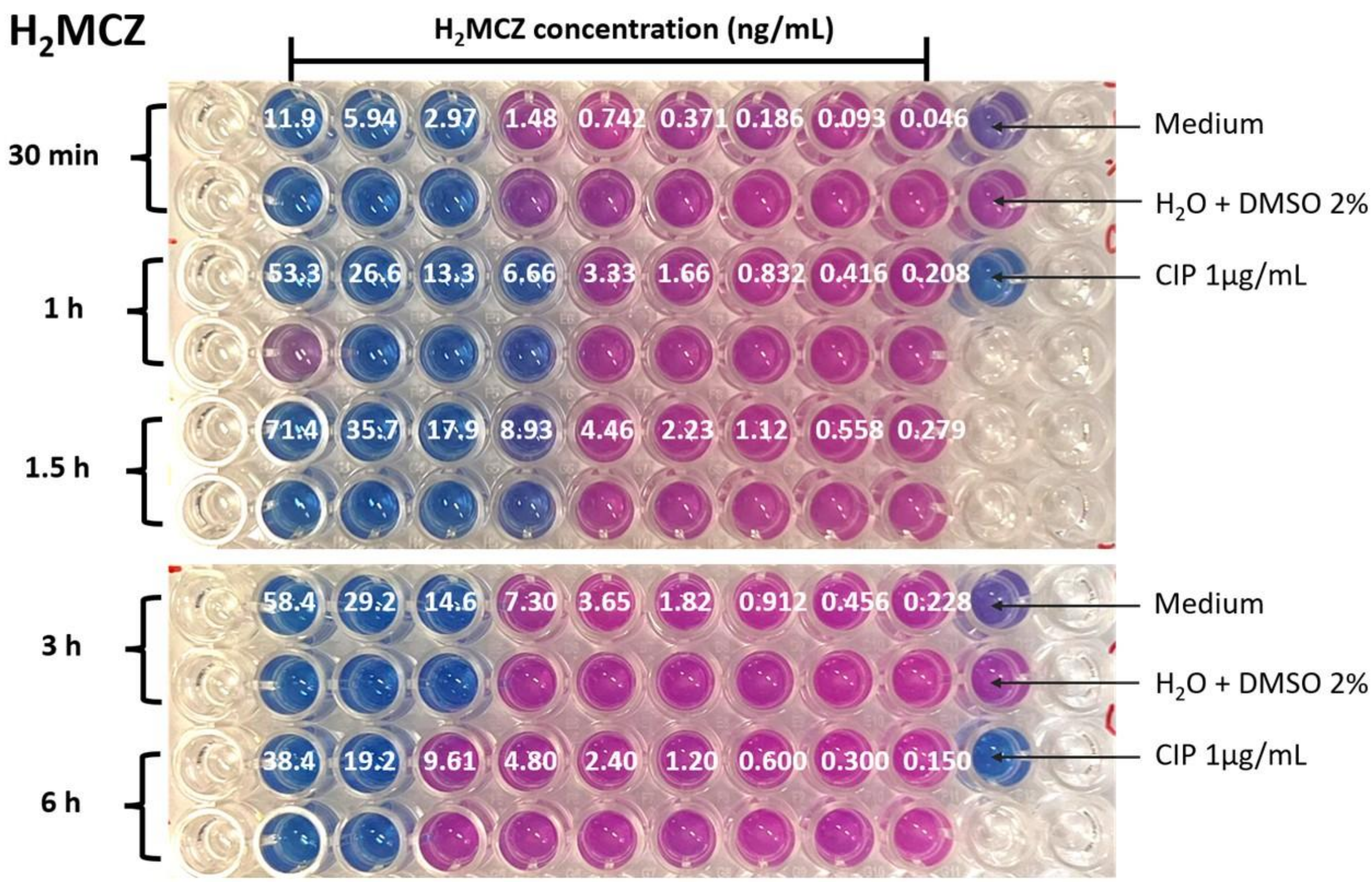

Appendix Figure S2 | H_2MCZ concentrations in wells, determined by LC-MS/MS. Serial dilutions of one volunteer's samples on day-14 (details indicated in **Figs. 6B and 6C**)

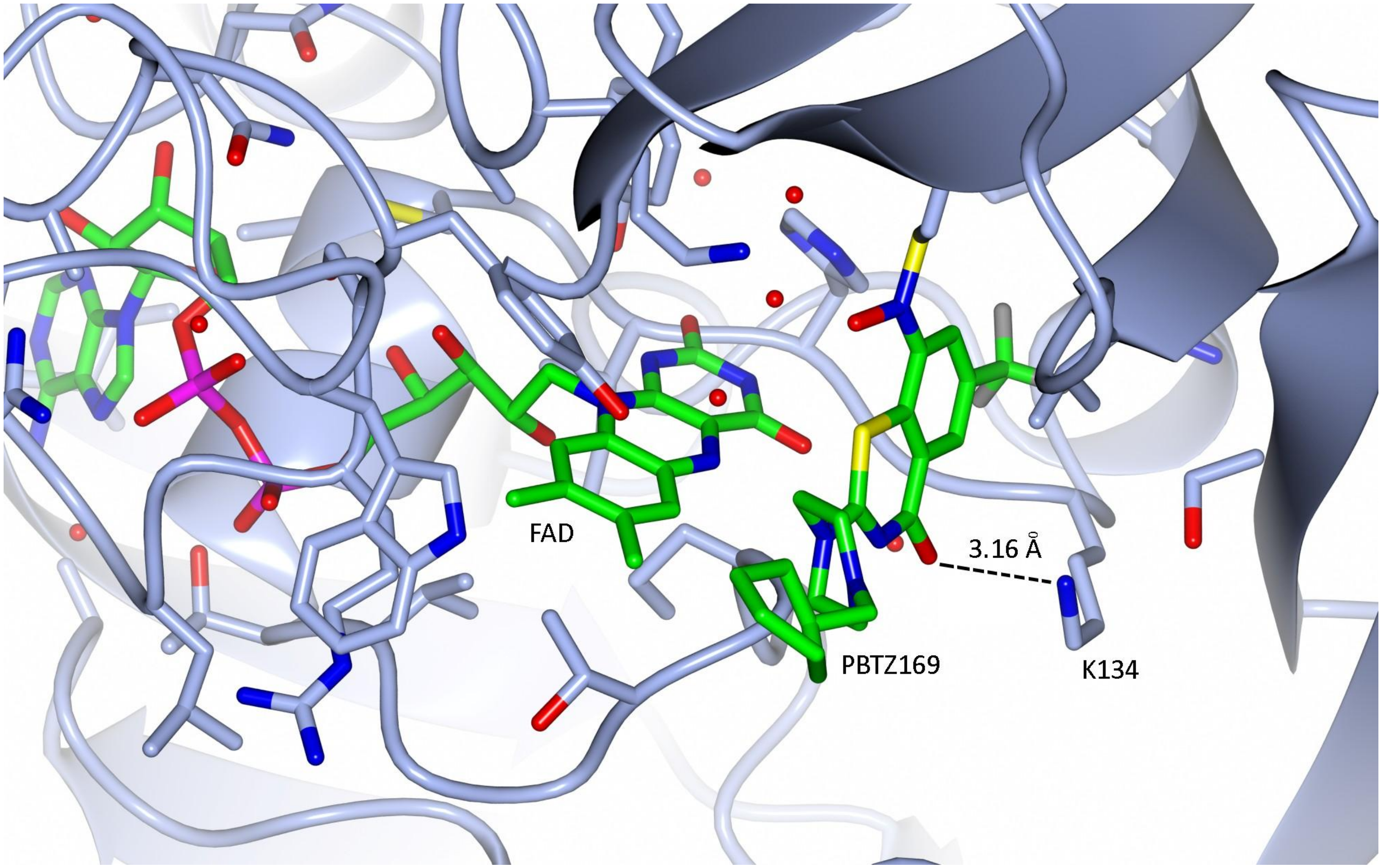

Appendix Figure S3 | Binding site of PBTZ169 in the DprE1 crystal structure (PDB ID: 4NCR). The oxygen atom of the benzothiazinone core is positioned opposite Lys134 at a distance of 3.16 Å. The negative charge localized on this oxygen atom may facilitate an electrostatic interaction with the ε-amino group of Lys134, potentially stabilizing the inhibitor within the DprE1 active site and thereby enhancing the enzyme's affinity for PBTZ169. The image was generated using CCP4MG version 2.10.11.

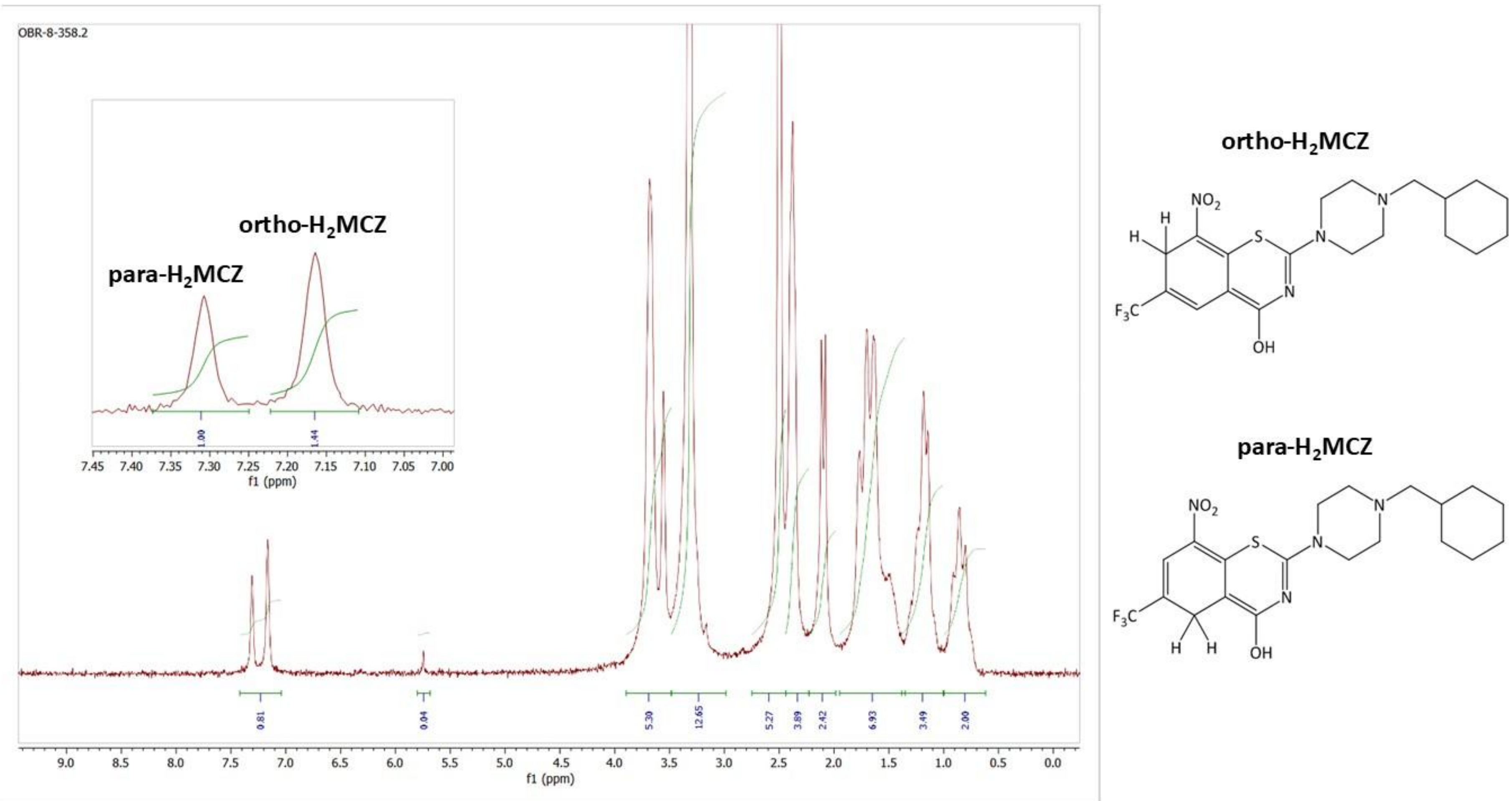

Appendix Figure S4 | ^{1}HNMR spectrum of synthetic mixture of ortho- and para-H_2MCZ

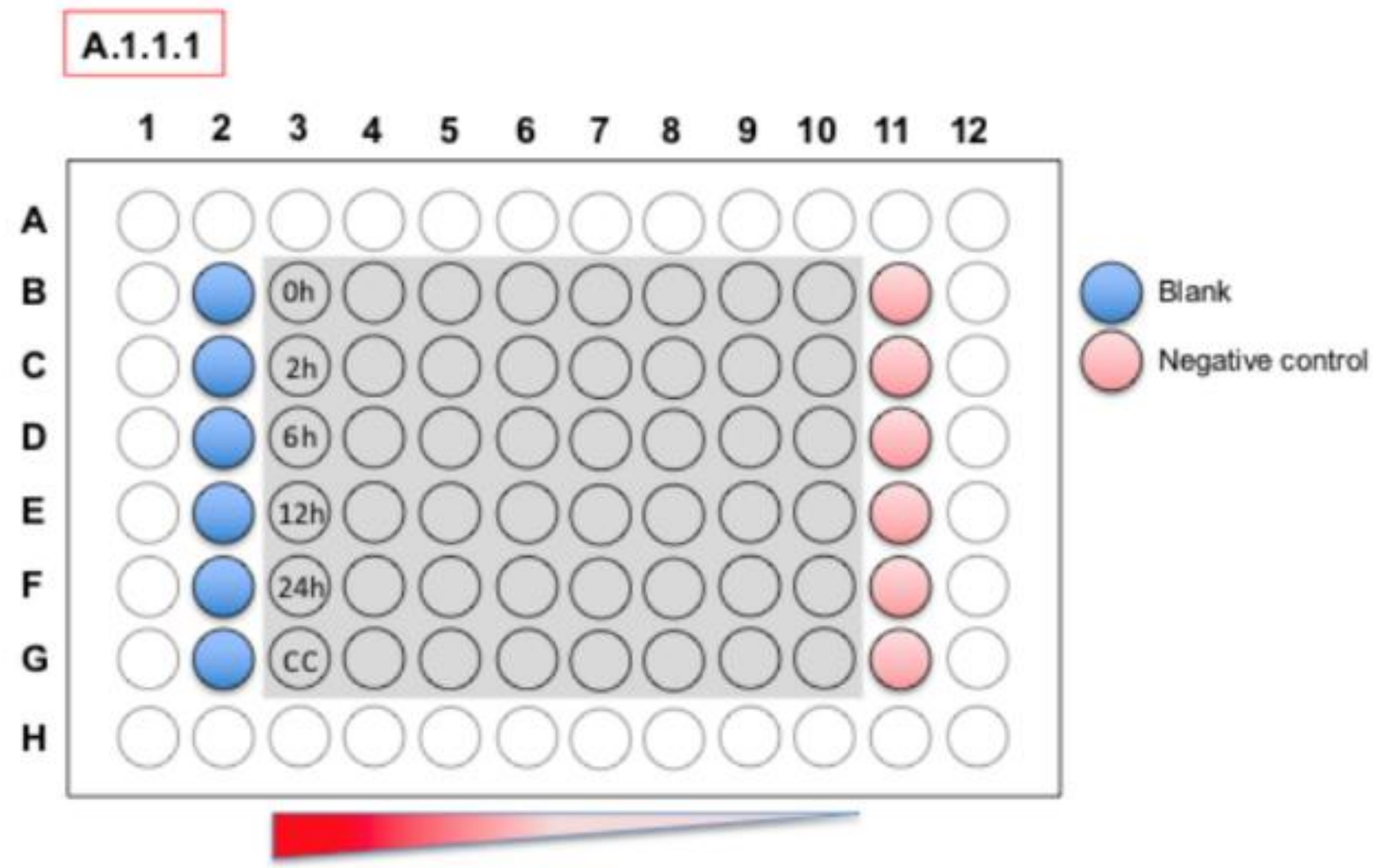

Appendix Figure S5 | Microplate preparation set up for *ex vivo* serum samples MIC determination (in

For preparation of the microplates, 50 µL of fresh culture medium was distributed in all wells of columns 1 and 2 and from columns 4 through 12 using a multi-channel pipette. In wells B through F of column 3, 100 µL of the sample was added, according to the microplate layout. In well G3, 100 µL of the MCZ spiked human serum was added and this served as the control lane (CC). In multiple instances, lane H (columns 3 through 10) was used to analyze the MIC for PBTZ169 diluted in culture medium, as a second control. The starting concentration of MCZ in either the serum or the culture medium control lanes was 20 ng/mL. Two-fold serial dilutions were performed on lines B through G from column 3 towards column 10 containing culture medium with a total volume of 50 µL using a multi-channel pipette. Thus, column 3 had the highest percentage of serum and column 10 the lowest. The final volume in all wells was 50 µL. 200 µL of frozen stock aliquots of M. smegmatis mc2155 were thawed and diluted into 10 mL of fresh sterile culture medium to an optical density at 600 nm (OD600) of 0.0001, representative of a concentration of 3 x 10^4 bacteria/mL. 50 µL of this mycobacterial suspension was added to the microplate wells from line B through G from columns 3 through 11, to obtain a final incubation volume of 100 µL per well. Column 2 in every plate was used as blank control wells (with no bacteria and serum but culture media only), and column 11 as the negative control or 100% growth (with bacteria only and 0% serum). The microplates were sealed using transparent film (Platemax AxySeal) and incubated for 24 hours at 37°C. This was followed by the addition of 10 µL Alamar Blue reagent (1/10 of well volume) and overnight incubation at 37°C. Finally, fluorescence of the resazurin metabolite, resorufin, was determined using a TECAN Infinite M200 microplate reader with the following settings: excitation at 560 nm, emission at 590 nm, optimal gain, and 10 flashes).

Appendix Supplementary Methods

Liquid-chromatography coupled to mass spectrometry methods (N° 1-6)

The LC-MS/MS method used for MCZ and H_2MCZ analyses was adapted from previously validated assays developed and optimized in our laboratory for human clinical studies[41,42]. Analyses of DTNB and TNB were performed by LC-MS/MS (ThermoFisher) with an Acquity BEH C18 column 1.7 µm (Waters) using a 3 min-gradient elution program composed of formic acid (FA) 2% in water and ACN while monitoring the mass transition at *m/z* 395.0 → 307.0, and *m/z* 197.0 → 154.1, for DTNB and TNB, respectively. The analyses of GSSG and GSH were performed by LC-MS/MS on an Xselect- HSS T3 3.5 µm 75 × 2.1 mm (Waters) using an 8 min-gradient elution of water + 0.1% FA and ACN (+ 0.1% FA). GSSG and GSH were monitored using the mass transition at *m/z* 613.0 → 355.0 and 308.0 → 162.0, respectively. Duroquinone and duroquinol were analysed by LC-MS on Xselect- HSS T3 3.5 µm 75 × 2.1 mm (Waters) using a 5 min gradient elution of 0.2% FA in water and ACN, and single ion monitoring (SIM) for detection. For our investigation on glutathion transferase activity, the glutathionylation reaction of the substrate 1-chloro-2,4,dinitrobenzene was followed by LC-MS/MS placed under a ventilated hood, on an Xselect- HSS T3 column 3.5 µm 75 × 2.1 mm (Waters) (gradient 0.1% FA in water/ ACN 0.1% FA), and the obtained product GS-DNG was monitored using the mass transition at *m/z* 474.0→ 242.0, 34.05, and 399.0). For the glutaredoxin study, the substrate ascorbic acid was analysed on an XBridge BEH Amide XP column (2.5 µM, 21x50 mm, Waters), using an X-min gradient elution composed of ammonium acetate 2.5 mM, pH 5.3, and ACN. Tandem mass spectrometry parameters and gradient elution programs for the above LC-MS/MS analyses are given in Tables S.2 to S.7.

The presence of the postulated glutathionyl-MCZ (PBTZ-SG) adduct in PLC incubation extracts has been examined successively first by LC-MS/MS over the range of *m/z* = 300-800, using the Single Ion Monitoring (SIM) mode, and then by Orbitrap High Resolution Mass Spectrometry searching in positive and negative ionization mode for the putative theoretical molecular weight *m/z* 763.80 for $C_{30}H_{40}F_3N_7O_9S_2$.

LC-MS/MS method N° 1 - LC-MS/MS parameters, solutions and components for MCZ/H_2MCZ analysis.

MCZ and H_2MCZ

Aqueous phase	Ammonium acetate 2.5mM, pH 5.3	143µL CH_3COOH<br>185µL NH_4OH<br>1L H_2O milliQ, adjust pH to 5.3
Organic phase	Acetonitrile	100% ACN
Precipitation solution and internal standard	IS-MCZ$_{d11}$ 80ng/mL<br>IS-H_2MCZ (compound 11526102) 40ng/mL	4µL MCZ$_{d11}$ 1mg/mL<br>2µL H_2MCZ ISTD 1mg/mL<br>49.994mL ACN
Column	XSelect® HSS T3 3.5µm, 2.1x75mm + AJ0-7600 SecurityGuard Cartridge, Polar C18, 4 x 2.0mm ID, 10pk	
Column temperature	40°C	
Injection volume	3µL	

Mobile phase gradient

Time (min)	Flow (mL/min)	% Organic phase	Curve
0.0	0.600	15	5
0.4	0.600	35	5
2.7	0.600	52	5
3.5	0.600	95	5
4.7	0.600	95	5
4.8	0.600	15	5
6.0	0.600	15	5

SRM table (ESI+)

Compound	Precursor (m/z)	Product (m/z)	Collision energy (V)
H_2MCZ	459.2	442.1	20
11526102-ISTD	255.2	240	23
MCZ	457.3	344	24
MCZ$_{d11}$-ISTD	468.4	318	24

Ion source properties

Pos. Ion spray voltage (V)	4000
Sheath gas (Arb)	35
Aux gas (Arb)	15
Sweep gas (Arb)	0
Ion transfer tube temp. (°C)	350
Vaporizer temp. (°C)	350

Chromatograms

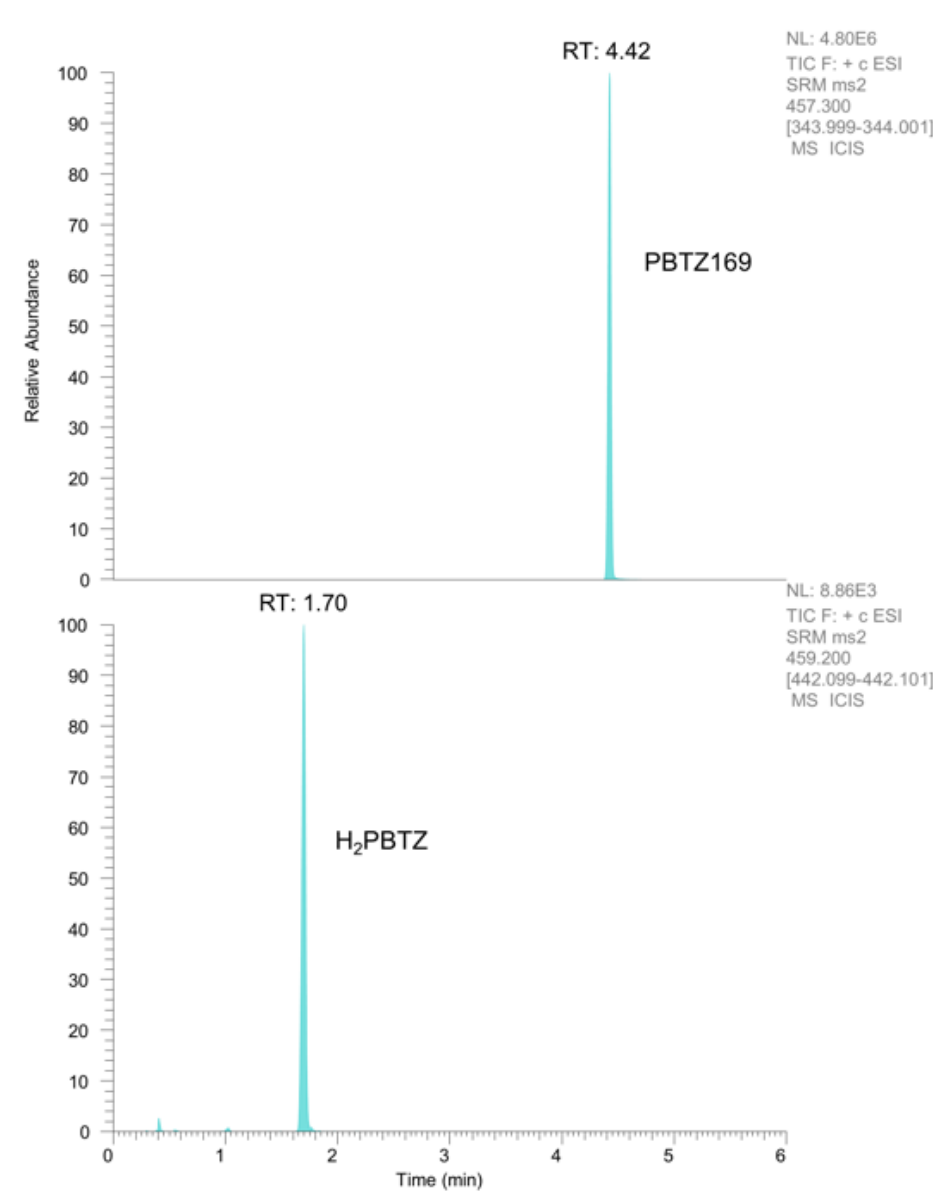

LC-MS/MS method for MCZ and H_2MCZ – modifications to the previous method in the context of MIC assays.

The LC-MS/MS method for MCZ and H_2MCZ was adapted over the course of our investigations, with the following modifications.

A solution of L-ascorbic acid (0.1 g/mL) was prepared in Milli-Q water and used as anti-oxidant to prevent the oxidation of H_2MCZ. Working solutions of MCZ and H_2MCZ at 10 µg/mL were obtained by diluting 10 µL of stock solution (1 mg/mL) with 990 µL EtOH/L-ascorbic acid (10:1 v/v). Calibration curves for both analytes were generated by serial dilution of the working solutions in EtOH/L-ascorbic acid (10:1 v/v), yielding concentrations ranging from 100 ng/mL to 0.2 ng/mL.

Internal standard (ISTD) working solution was prepared by mixing 10 µL of MCZ-d_{11} and 10 µL of H_2MCZ-ISTD (compound 11526102) stock solutions with 2485 µL ACN, followed by 100-fold dilution in MeOH/L-ascorbic acid (10:1 v/v).

When required, samples were first diluted with ethanol to ensure that their concentrations fell within the calibration range. For sample preparation, 90 µL of the ISTD solution was added to 30 µL of the sample or calibration standard, vortexed, and centrifuged (10 min, 17,130 g, 4°C). A 90 µL supernatant aliquot was transferred to polypropylene HPLC vials with conical inserts, mixed with 90 µL L-ascorbic acid solution (0.01 g/mL in Milli-Q water), sealed, and vortexed before LC-MS/MS analysis.

Chromatographic separation and mass spectrometry were performed as described previously, except that the aqueous mobile phase was modified to 0.1% formic acid in water instead of 2.5 mM ammonium acetate buffer (pH 5.3).

Data were acquired and processed using XCalibur version 4.2 (Thermo Fisher Scientific, San Jose, CA, USA), and final concentrations were calculated by applying appropriate dilution factors.

LC-MS/MS method N° 2 - LC-MS/MS parameters, solutions and components for DTNB/TNB analysis.

<table>
<tr><td rowspan="25">DTNB and TNB</td><td>Aqueous phase</td><td colspan="2">H_2O milliQ 2% formic acid (v/v)</td><td colspan="2">20mL Formic acid
980mL H_2O milliQ</td></tr>
<tr><td>Organic phase</td><td colspan="2">Acetonitrile</td><td colspan="2">100% ACN</td></tr>
<tr><td>Precipitation solution</td><td colspan="2">Acetonitrile</td><td colspan="2">100% ACN</td></tr>
<tr><td>Column</td><td colspan="4">Acquity UPLC® BEH C18 1.7µM (tolerates very low pH)</td></tr>
<tr><td>Column temperature</td><td colspan="4">25°C</td></tr>
<tr><td>Injection volume</td><td colspan="4">2µL</td></tr>
<tr><td rowspan="5">Mobile phase gradient</td><td>Time (min)</td><td>Flow (mL/min)</td><td>% Organic phase</td><td>Curve</td></tr>
<tr><td>0.0</td><td>0.500</td><td>15</td><td>5</td></tr>
<tr><td>1.9</td><td>0.500</td><td>95</td><td>5</td></tr>
<tr><td>2.0</td><td>0.500</td><td>15</td><td>5</td></tr>
<tr><td>3.0</td><td>0.500</td><td>15</td><td>5</td></tr>
<tr><td rowspan="3">SRM table (ESI-)</td><td>Compound</td><td>Precursor (m/z)</td><td>Product (m/z)</td><td>Collision energy (V)</td></tr>
<tr><td>TNB</td><td>197.9</td><td>154.1</td><td>10</td></tr>
<tr><td>DTNB</td><td>395</td><td>307</td><td>11</td></tr>
<tr><td rowspan="6">Ion source properties</td><td colspan="2">Neg. Ion spray voltage (V)</td><td colspan="2">2500</td></tr>
<tr><td colspan="2">Sheath gas (Arb)</td><td colspan="2">50</td></tr>
<tr><td colspan="2">Aux gas (Arb)</td><td colspan="2">5</td></tr>
<tr><td colspan="2">Sweep gas (Arb)</td><td colspan="2">0</td></tr>
<tr><td colspan="2">Ion transfer tube temp. (°C)</td><td colspan="2">350</td></tr>
<tr><td colspan="2">Vaporizer temp. (°C)</td><td colspan="2">350</td></tr>
<tr><td>Chromatograms</td><td colspan="4">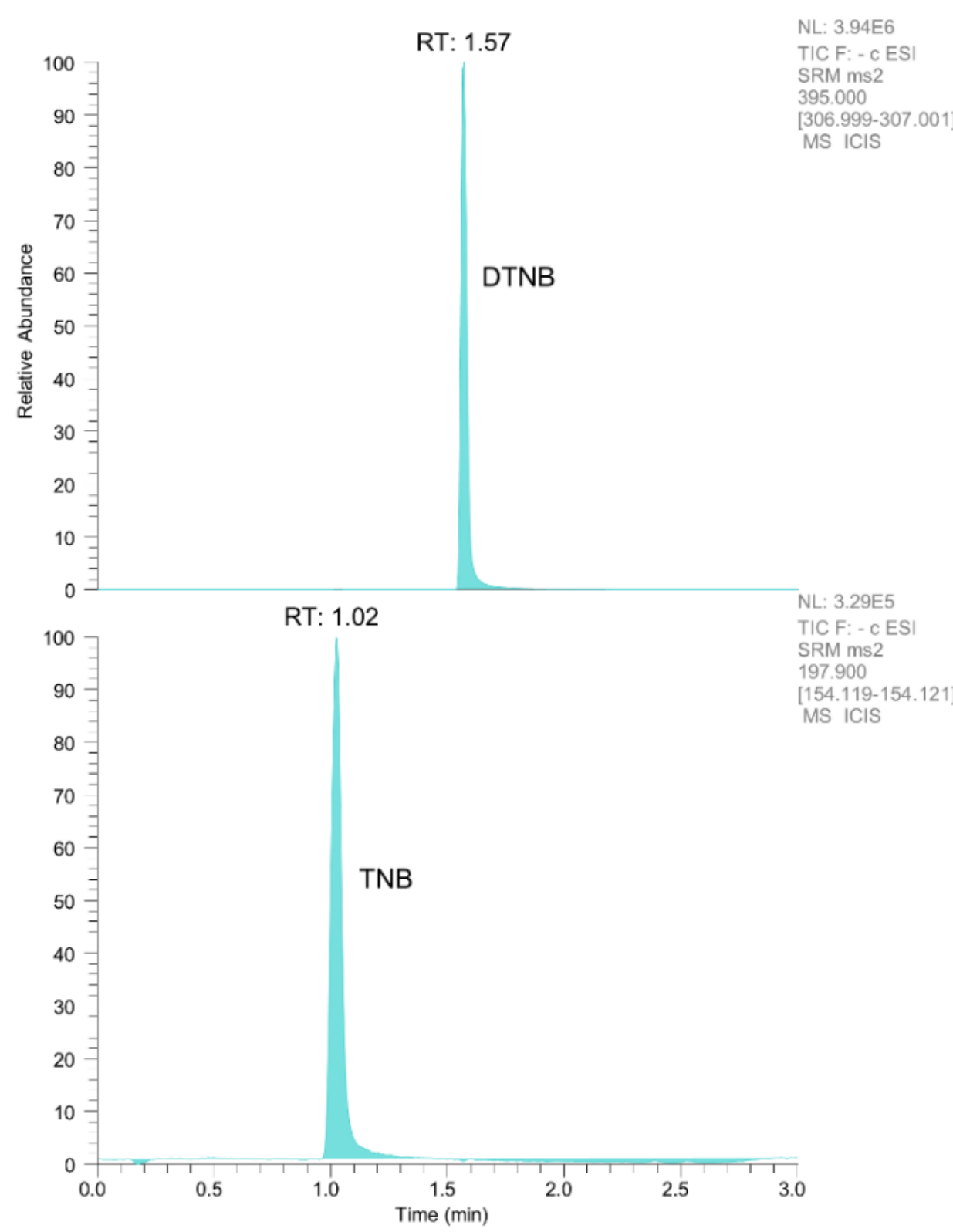</td></tr>
</table>

LC-MS/MS method N° 3 - LC-MS/MS parameters, solutions and components for GSSG/GSH analysis.

GSSG and GSH	Aqueous phase	H_2O milliQ 0.1% formic acid (v/v)		1mL Formic acid<br>999mL H_2O milliQ	
	Organic phase	ACN 0.1% formic acid (v/v)		1mL Formic acid<br>999mL ACN	
	Precipitation solution	Acetonitrile		100% ACN	
	Column	XSelect® HSS T3 3.5µm, 2.1x75mm + AJ0-7600 SecurityGuard Cartridge, Polar C18, 4 x 2.0mm ID, 10pk			
	Column temperature	25°C			
	Injection volume	3µL (GSH analysis: dilute supernatant 10x in H_2O in the vial prior to injection)			
	Mobile phase gradient	**Time (min)**	**Flow (mL/min)**	**% Organic phase**	**Curve**
		0.0	0.400	0	5
		1.3	0.400	0	5
		3.0	0.400	90	5
		3.1	0.600	0	5
		8.0	0.400	0	5
	SRM table (ESI+)	**Compound**	**Precursor (m/z)**	**Product (m/z)**	**Collision energy (V)**
		GSH	308	162.1	16
		GSSG	613.2	355.1	22
	Ion source properties	Pos. Ion spray voltage (V)		3500	
		Sheath gas (Arb)		40	
		Aux gas (Arb)		15	
		Sweep gas (Arb)		0	
		Ion transfer tube temp. (°C)		350	
		Vaporizer temp. (°C)		150	
	Chromatograms	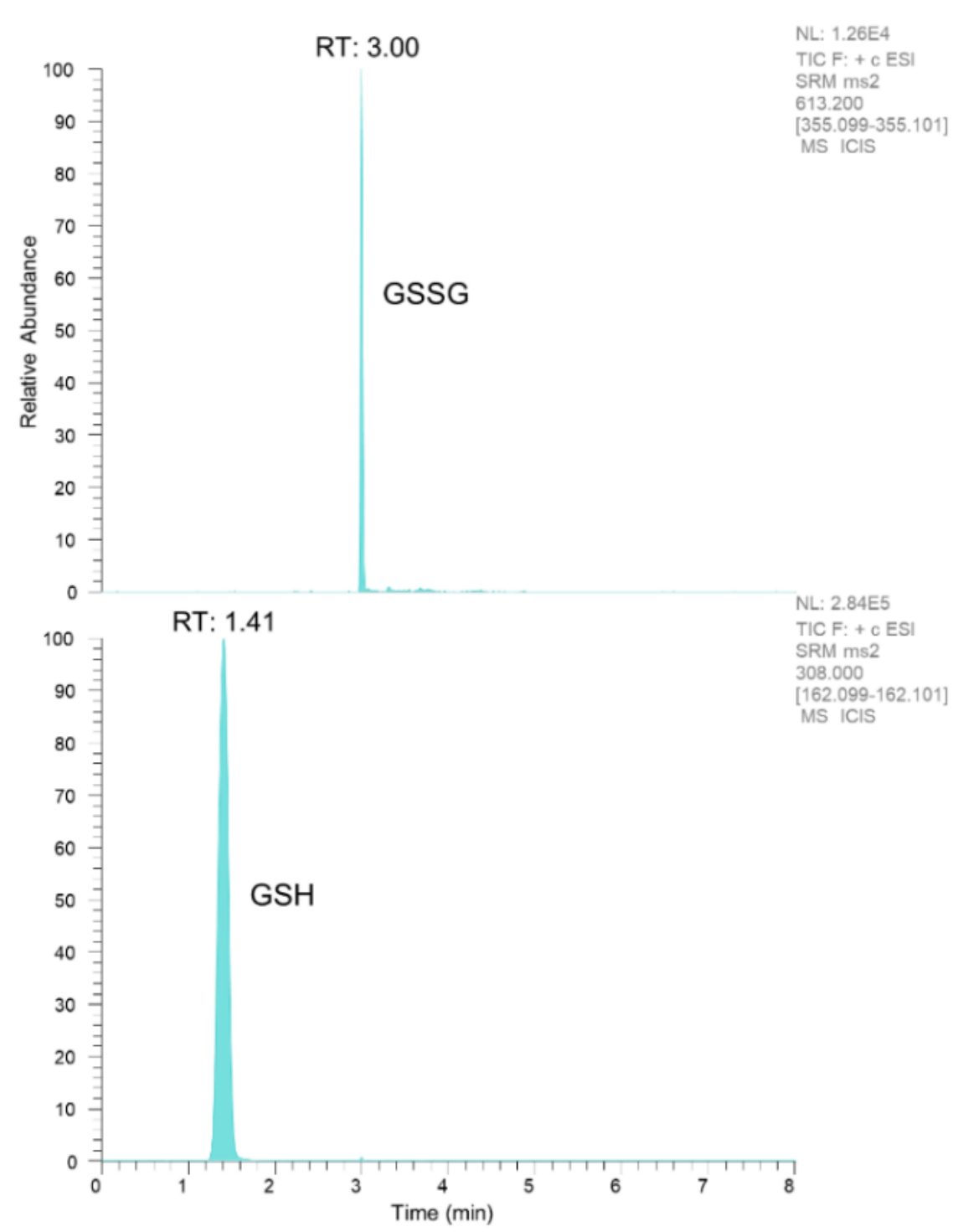			

LC-MS/MS method N° 4 – LC-MS/MS parameters, solutions and components for duroquinone and duroquinol analysis.

<table>
<tr><td rowspan="24">Duroquinone and duroquinol</td><td>Aqueous phase</td><td>H_2O milliQ 0.2% formic acid (v/v)</td><td colspan="3">2mL Formic acid
998mL H_2O milliQ</td></tr>
<tr><td>Organic phase</td><td>Acetonitrile</td><td colspan="3">100% ACN</td></tr>
<tr><td>Precipitation solution</td><td>Acetonitrile</td><td colspan="3">100% ACN</td></tr>
<tr><td>Column</td><td colspan="4">XSelect® HSS T3 3.5µm, 21 x75mm + AJ0-7600 SecurityGuard Cartridge, Polar C18, 4 x 2.0mm ID, 10pk</td></tr>
<tr><td>Column temperature</td><td colspan="4">40°C</td></tr>
<tr><td>Injection volume</td><td colspan="4">3µL</td></tr>
<tr><td rowspan="5">Mobile phase gradient</td><td>Time (min)</td><td>Flow (mL/min)</td><td>% Organic phase</td><td>Curve</td></tr>
<tr><td>0.0</td><td>0.500</td><td>15</td><td>5</td></tr>
<tr><td>3.5</td><td>0.500</td><td>95</td><td>5</td></tr>
<tr><td>3.6</td><td>0.500</td><td>15</td><td>5</td></tr>
<tr><td>5.0</td><td>0.500</td><td>15</td><td>5</td></tr>
<tr><td rowspan="3">SIM table (ESI-)</td><td>Compound</td><td colspan="3">Center mass (m/z)</td></tr>
<tr><td>Duroquinone</td><td colspan="3">164.0</td></tr>
<tr><td>Duroquinol</td><td colspan="3">164.0</td></tr>
<tr><td rowspan="6">Ion source properties</td><td>Neg. Ion spray voltage (V)</td><td colspan="3">2500</td></tr>
<tr><td>Sheath gas (Arb)</td><td colspan="3">50</td></tr>
<tr><td>Aux gas (Arb)</td><td colspan="3">5</td></tr>
<tr><td>Sweep gas (Arb)</td><td colspan="3">0</td></tr>
<tr><td>Ion transfer tube temp. (°C)</td><td colspan="3">350</td></tr>
<tr><td>Vaporizer temp. (°C)</td><td colspan="3">350</td></tr>
<tr><td>Chromatogram</td><td colspan="4">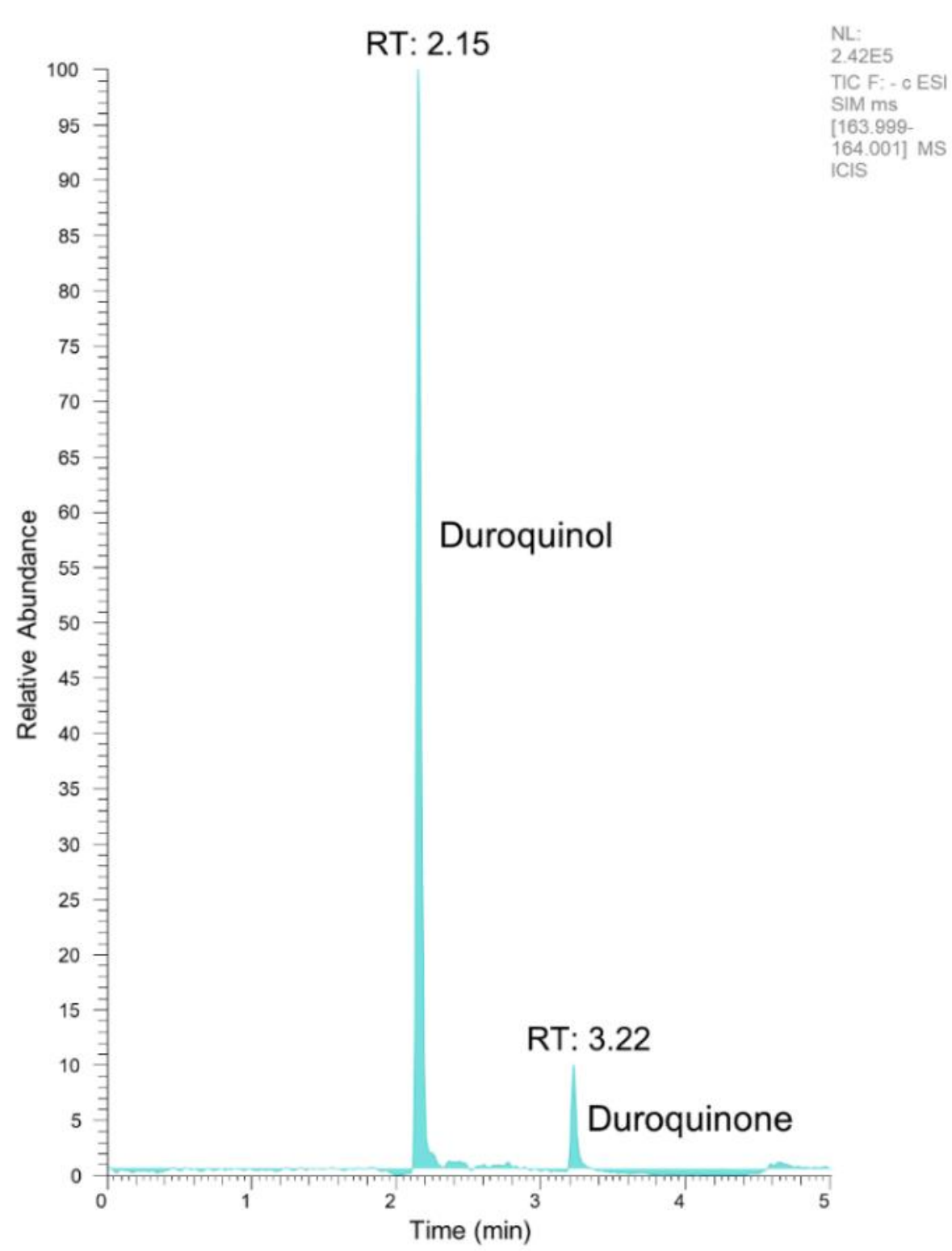</td></tr>
</table>

LC-MS/MS method N° 5 - LC-MS/MS parameters, solutions and components for GS-DNB analysis. Specification: due to the toxicity of the substrate 1-chloro-2,4-dinitrobenzene, analyses are performed using a LC-MS/MS instrument under a ventilated hood.

<table>
<tr><td rowspan="24">GS-DNB</td><td>Aqueous phase</td><td colspan="2">H_2O milliQ 0.1% formic acid (v/v)</td><td colspan="2">1mL Formic acid
999mL H_2O milliQ</td></tr>
<tr><td>Organic phase</td><td colspan="2">ACN 0.1% formic acid (v/v)</td><td colspan="2">1mL Formic acid
999mL ACN</td></tr>
<tr><td>Precipitation solution and internal standard</td><td colspan="2">Acetonitrile</td><td colspan="2">100% ACN</td></tr>
<tr><td>Column</td><td colspan="4">XSelect® HSS T3 3.5µm, 2.1x75mm</td></tr>
<tr><td>Column temperature</td><td colspan="4">25°C</td></tr>
<tr><td>Injection volume</td><td colspan="4">3µL</td></tr>
<tr><td rowspan="5">Mobile phase gradient</td><td>Time (min)</td><td>Flow (mL/min)</td><td>% Organic phase</td><td>Curve</td></tr>
<tr><td>0.0</td><td>0.400</td><td>5</td><td>5</td></tr>
<tr><td>2.0</td><td>0.400</td><td>95</td><td>5</td></tr>
<tr><td>2.1</td><td>0.400</td><td>5</td><td>5</td></tr>
<tr><td>4.0</td><td>0.400</td><td>5</td><td>5</td></tr>
<tr><td rowspan="4">SRM table (ESI+)</td><td>Compound</td><td>Precursor (m/z)</td><td>Product (m/z)</td><td>Collision energy (V)</td></tr>
<tr><td rowspan="3">GS-DNB</td><td rowspan="3">474.1</td><td>242.0</td><td>21</td></tr>
<tr><td>345.0</td><td>15</td></tr>
<tr><td>399.0</td><td>17</td></tr>
<tr><td rowspan="6">Ion source properties</td><td colspan="2">Pos. Ion spray voltage (V)</td><td colspan="2">2500</td></tr>
<tr><td colspan="2">Sheath gas (Arb)</td><td colspan="2">50</td></tr>
<tr><td colspan="2">Aux gas (Arb)</td><td colspan="2">5</td></tr>
<tr><td colspan="2">Sweep gas (Arb)</td><td colspan="2">0</td></tr>
<tr><td colspan="2">Ion transfer tube temp. (°C)</td><td colspan="2">350</td></tr>
<tr><td colspan="2">Vaporizer temp. (°C)</td><td colspan="2">350</td></tr>
<tr><td>Chromatograms</td><td colspan="4">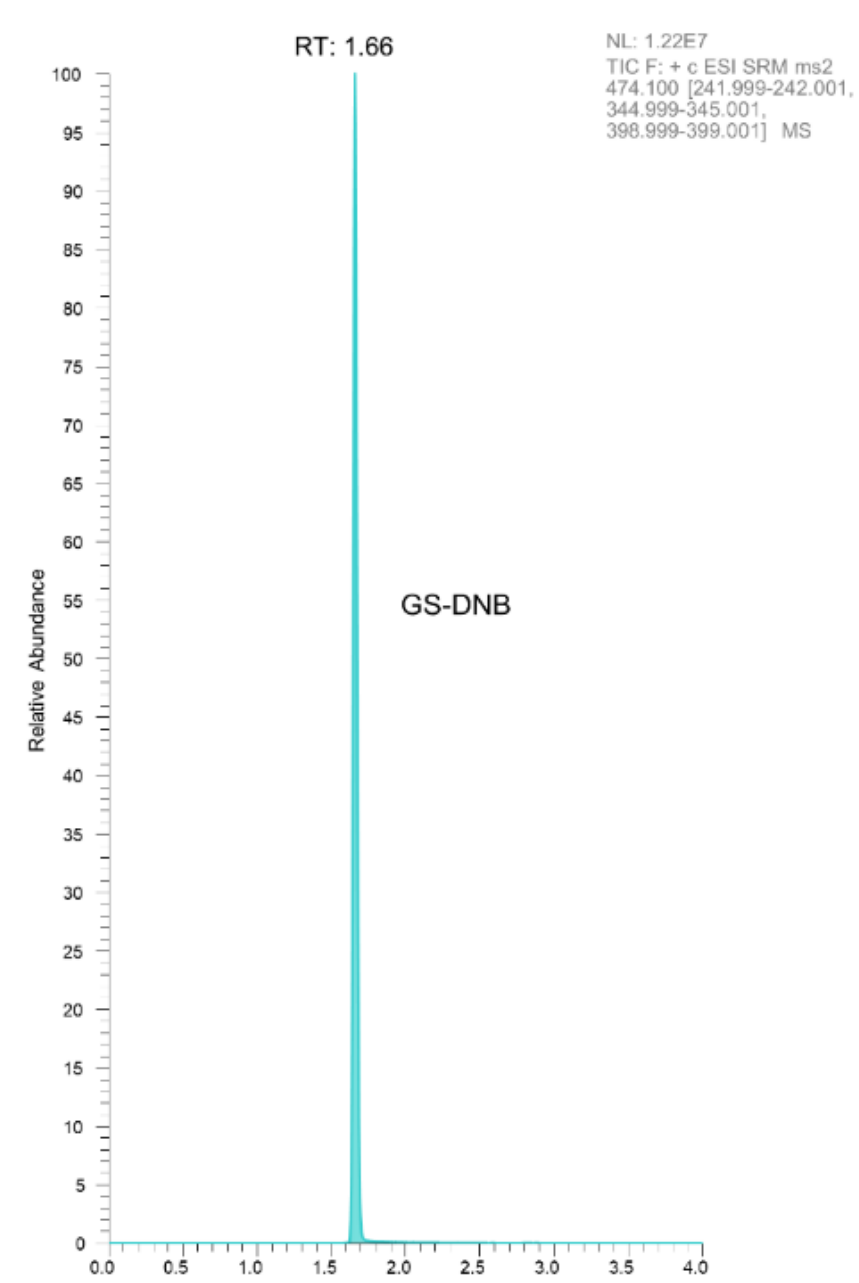</td></tr>
</table>

LC-MS/MS method N° 6 - LC-MS/MS method, solutions and component for ascorbic acid analysis.

<table>
<tr><td rowspan="21">Ascorbic acid</td><td>Aqueous phase</td><td>Ammonium acetate 2.5mM, pH 5.3</td><td colspan="2">143µL CH_3COOH
185µL NH_4OH
1L H_2O milliQ
Adjust pH to 5.3</td></tr>
<tr><td>Organic phase</td><td>Acetonitrile</td><td colspan="2">100% ACN</td></tr>
<tr><td>Precipitation solution</td><td>Acetonitrile</td><td colspan="2">100% ACN</td></tr>
<tr><td>Column</td><td colspan="4">XBridge® BEH Amide 2.5µM 2.1x50mm Column XP</td></tr>
<tr><td>Column temperature</td><td colspan="4">40°C</td></tr>
<tr><td>Injection volume</td><td colspan="4">3µL</td></tr>
<tr><td rowspan="8">Mobile phase gradient</td><td>Time (min)</td><td>Flow (mL/min)</td><td>% Organic phase</td><td>Curve</td></tr>
<tr><td>0.0</td><td>0.400</td><td>95</td><td>5</td></tr>
<tr><td>0.5</td><td>0.400</td><td>95</td><td>5</td></tr>
<tr><td>2.0</td><td>0.400</td><td>40</td><td>5</td></tr>
<tr><td>2.5</td><td>0.400</td><td>40</td><td>5</td></tr>
<tr><td>2.6</td><td>0.600</td><td>95</td><td>5</td></tr>
<tr><td>4.9</td><td>0.600</td><td>95</td><td>5</td></tr>
<tr><td>5.0</td><td>0.400</td><td>95</td><td>5</td></tr>
<tr><td rowspan="2">SRM table (ESI-)</td><td>Compound</td><td>Precursor (m/z)</td><td>Product (m/z)</td><td>Collision energy (V)</td></tr>
<tr><td>Ascorbic acid</td><td>174.8</td><td>86.9</td><td>18</td></tr>
<tr><td rowspan="6">Ion source properties</td><td colspan="2">Neg. Ion spray voltage (V)</td><td colspan="2">2500</td></tr>
<tr><td colspan="2">Sheath gas (Arb)</td><td colspan="2">55</td></tr>
<tr><td colspan="2">Aux gas (Arb)</td><td colspan="2">15</td></tr>
<tr><td colspan="2">Sweep gas (Arb)</td><td colspan="2">0</td></tr>
<tr><td colspan="2">Ion transfer tube temp. (°C)</td><td colspan="2">350</td></tr>
<tr><td></td><td>Vaporizer temp. (°C)</td><td colspan="2"></td><td colspan="2">250</td></tr>
<tr><td></td><td>Chromatograms</td><td colspan="4">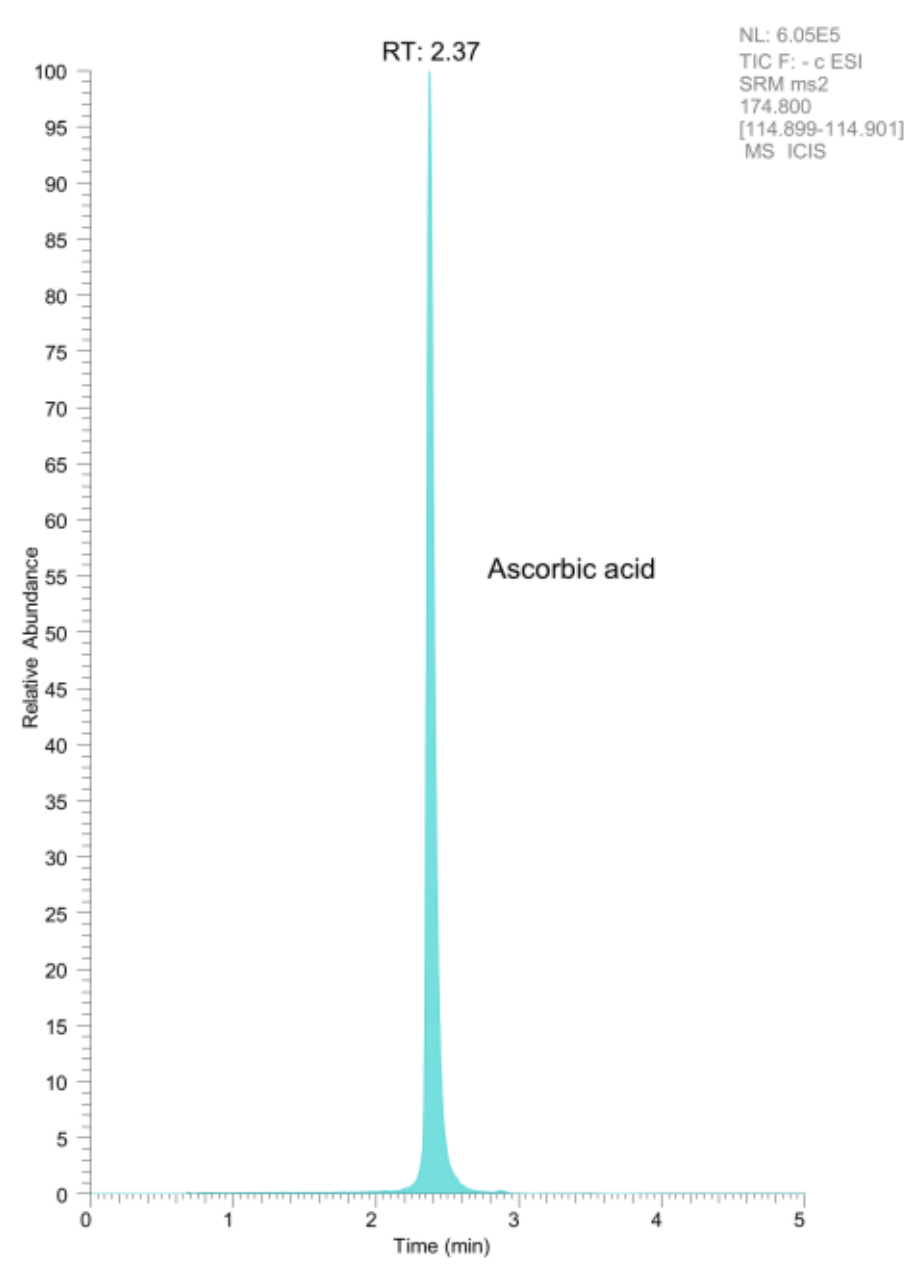</td></tr>
</table>

References (References cited in Appendix Table S1 that are not included in the main text)

Ahmad R, Srivastava AK (2008) Inhibition of glutathione-S-transferase from Plasmodium yoelii by protoporphyrin IX, cibacron blue and menadione: implications and therapeutic benefits. *Parasitol Res* 102: 805-807.

de Souza LF, Schmitz AE, da Silva LCS, de Oliveira KA, Nedel CB, Tasca CI, et al. (2017) Inhibition of reductase systems by 2-AAPA modulates peroxiredoxin oxidation and mitochondrial function in A172 glioblastoma cells. *Toxicol In Vitro* 42: 273-280.

Hayeshi R, Mutingwende I, Mavengere W, Masiyanise V and S. Mukanganyama S (2007) The inhibition of human glutathione S-transferases activity by plant polyphenolic compounds ellagic acid and curcumin. *Food Chem Toxicol* 45: 286-295.

Kitamura S, Katsura H, Tatsumi K (1996) Multiplicity of rat liver 15-ketoprostaglandin delta 13-reductases *Prostaglandins* 52: 35-49.

Sadhu SS, Callegari E, Zhao Y, Guan X, Seefeldt T (2013) Evaluation of a dithiocarbamate derivative as an inhibitor of human glutaredoxin-1. *J Enzyme Inhib Med Chem* 28: 456-462.

Zhang N, Sun S, Zhang Y, Yang Y, Zhang Y, Chen J, et al. (2020) Selenoprotein thioredoxin reductase mediated menadione reduction: catalytic properties & inhibition effects. *Sheng Wu Gong Cheng Xue Bao* 36 : 2139-2150.
