# Supplementary material for: Human prostaglandin reductases dearomatize and inactivate benzothiazinone antitubercular drugs
Source: EMBO Mol Med. 2026 Oct 7;18(11):4413–32. doi: 10.1038/s44321-026-00526-1 (PMC13748546; doi:10.1038/s44321-026-00526-1)
Supplement: Supplementary file 2 [file 44321_2026_526_MOESM2_ESM.pdf]

EMBO Molecular Medicine

Human prostaglandin reductases dearomatize and inactivate benzothiazinone antitubercular drugs

Myriam Briki, Caroline Wagner, Vincent Desfontaine, Anthony Vocat, Onya Opota, Sarah Abou-Zite, Océane Corpataux, Benedetta Cena, Sylvie Guinchard, François Versace, Amarande Murisier, Thomas Mercier, Emilyne Blattes, Carine Bardinet, Paul Thoueille, Vladimir Wsol, Haithem Chtioui, Laura Rothuizen, Aimable Nahimana, Axel Bellotti, Laurent Chiarelli, Dany Spaggiari, Neeraj Dhar, Richa Mishra, Raphael Sommer, Andréanne Lupien, Michel Duchosal, Benoît Guery, François Girardin, Giuseppina Milano, Vadim Makarov, Thierry Buclin, Stewart Cole, Eva Choong, Lastovickova Lenka, Olga Riabova, and Laurent Decosterd

Corresponding author: Laurent Decosterd (LaurentArthur.Decosterd@chuv.ch)

Review Timeline:

Submission Date:	16th Feb 26
Editorial Decision:	2nd Apr 26
Revision Received:	1st Jul 26
Editorial Decision:	22nd Jul 26
Revision Received:	28th Aug 26
Accepted:	8th Sep 26

Editor: Zeljko Durdevic

Transaction Report:

(Note: With the exception of the correction of typographical or spelling errors that could be a source of ambiguity, letters and reports are not edited. Depending on transfer agreements, referee reports obtained elsewhere may or may not be included in this compilation. Referee reports are anonymous unless the Referee chooses to sign their reports.)

2nd Apr 2026

Dear Prof. Decosterd,

Thank you for the submission of your manuscript to EMBO Molecular Medicine. We have now received feedback from the three reviewers who agreed to evaluate your manuscript. All three referees are overall supportive but also raise important concerns that should be addressed in a major revision. If you would like to discuss further the points raised by the referees, I am available to do so via email or video. Let me know if you are interested in this option.

Further consideration of a revision that addresses reviewers' concerns in full will entail a second round of review. EMBO Molecular Medicine encourages a single round of revision only and therefore, acceptance or rejection of the manuscript will depend on the completeness of your responses included in the next, final version of the manuscript. For this reason, and to save you from any frustrations in the end, I would strongly advise against returning an incomplete revision.

We would welcome the submission of a revised version within three months for further consideration. Please let us know if you require longer to complete the revision.

I look forward to receiving your revised manuscript.

Yours sincerely,

Zeljko Durdevic

Zeljko Durdevic
Senior Editor
EMBO Molecular Medicine

*****
When preparing your revised manuscript, please refer to our guidelines: https://link.springer.com/journal/44321/submission-guidelines#cms-Revised-submissions. We perform an initial quality control of all revised manuscripts before re-review; failure to include requested items will delay the evaluation of your revision.

We require:

1) A .docx formatted version of the manuscript text (including legends for main figures, EV figures and tables). Please make sure that the changes are highlighted to be clearly visible.

2) Individual production quality figure files as .eps, .tif, .jpg (one file per figure). For guidance, download the 'Figure Guide PDF': https://media.springernature.com/original/springer-cms/rest/v1/content/27825798/data/v1.

3) A .docx formatted letter INCLUDING the reviewers' reports and your detailed point-by-point responses to their comments. As part of the EMBO Press transparent editorial process, the point-by-point response is part of the Review Process File (RPF), which will be published alongside your paper.

4) A complete author checklist, which you can download from our author guidelines. Please insert information in the checklist that is also reflected in the manuscript. The completed author checklist will also be part of the RPF.

5) Please note that all corresponding authors are required to supply an ORCID ID for their name upon submission of a revised manuscript.

6) It is mandatory to include a 'Data Availability' section after the Materials and Methods. Before submitting your revision, primary datasets produced in this study need to be deposited in an appropriate public database, and the accession numbers and database listed under 'Data Availability'. Please remember to provide a reviewer password if the datasets are not yet public.

In case you have no data that requires deposition in a public database, please state so in this section. Note that the Data Availability Section is restricted to new primary data that are part of this study.

7) For data quantification: please specify the name of the statistical test used to generate error bars and P values, the number (n) of independent experiments (specify technical or biological replicates) underlying each data point and the test used to calculate p-values in each figure legend. The figure legends should contain a basic description of n, P and the test applied. Graphs must include a description of the bars and the error bars (s.d., s.e.m.).

8) At EMBO Press we ask authors to provide source data for the main manuscript figures. You will receive a separate email with instructions for providing source data with your revised manuscript, including how to upload and organize the files.

9) Our journal encourages inclusion of *data citations in the reference list* to directly cite datasets that were re-used and obtained from public databases. Data citations in the article text are distinct from normal bibliographical citations and should directly link to the database records from which the data can be accessed. In the main text, data citations are formatted as follows: "Data ref: Smith et al, 2001" or "Data ref: NCBI Sequence Read Archive PRJNA342805, 2017". In the Reference list, data citations must be labeled with "[DATASET]". A data reference must provide the database name, accession number/identifiers and a resolvable link to the landing page from which the data can be accessed at the end of the reference.

10) We replaced Supplementary Information with Expanded View (EV) Figures and Tables that are collapsible/expandable online. A maximum of 5 EV Figures can be typeset. EV Figures should be cited as 'Figure EV1, Figure EV2" etc... in the text and their respective legends should be included in the main text after the legends of regular figures.

- For the figures that you do NOT wish to display as Expanded View figures, they should be bundled together with their legends in a single PDF file called *Appendix*, which should start with a short Table of Content. Appendix figures should be referred to in the main text as: "Appendix Figure S1, Appendix Figure S2" etc.

- Additional Tables/Datasets should be labeled and referred to as Table EV1, Dataset EV1, etc. Legends have to be provided in a separate tab in case of .xls files. Alternatively, the legend can be supplied as a separate text file (README) and zipped together with the Table/Dataset file.

11) The paper explained: EMBO Molecular Medicine articles are accompanied by a summary of the articles to emphasize the major findings in the paper and their medical implications for the non-specialist reader. Please provide a draft summary of your article highlighting

- the medical issue you are addressing,

- the results obtained and

- their clinical impact.

This may be edited to ensure that readers understand the significance and context of the research. Please refer to any of our published articles for an example.

12) Author contributions: You will be asked to provide CRediT (Contributor Role Taxonomy) terms in the submission system. These replace a narrative author contribution section in the manuscript.

13) A Conflict of Interest statement should be provided in the main text.

14) Every published paper includes a 'Synopsis' to further enhance discoverability. Synopses are displayed on the journal webpage and are freely accessible to all readers. They include a short stand first (maximum of 300 characters, including space) as well as 2-5 one-sentences bullet points that summarizes the paper. Please write the bullet points to summarize the key NEW findings. They should be designed to be complementary to the abstract - i.e. not repeat the same text. We encourage inclusion of key acronyms and quantitative information (maximum of 30 words / bullet point). Please use the passive voice. Please attach these in a separate file or send them by email, we will incorporate them accordingly.

Please also suggest a striking image or visual abstract to illustrate your article as a PNG file 550 px wide x 300-600 px high.

15) Include a Reagents and Tools Table as part of the Methods section, which can be downloaded from our author guidelines.

EMBO Molecular Medicine has a "scooping protection" policy, whereby similar findings that are published by others during review or revision are not a criterion for rejection. Should you decide to submit a revised version, I do ask that you get in touch after three months if you have not completed it, to update us on the status.

***** Reviewer's comments *****

Referee #1 (Remarks for Author):

This manuscript by Briki et al. identifies human prostaglandin reductases PTGR1 and PTGR2 as the enzymes responsible for the NADPH-dependent dearomatization of the antitubercular drug macozinone (PBTZ169) into its Hydride Meisenheimer complex (H_2MCZ). The study further demonstrates that this metabolic conversion reduces antimycobacterial activity and can be modulated by known PTGR inhibitors. The work addresses a long-standing question in benzothiazinone pharmacology and reveals a previously unrecognized, noncanonical xenobiotic metabolic pathway in humans. The study is technically rigorous, comprehensive, and conceptually novel. Identification of PTGR1/2 as mediators of enzymatic dearomatization represents a significant advance with implications for anti-TB drug development and host-drug interactions. The conclusions are largely well supported by the data.

Major comments
1. PTGR1 vs PTGR2 contribution. The conclusion that PTGR2 is the principal in vivo enzyme is based mainly on recombinant enzyme activity. This should be stated more cautiously, with clearer discussion of tissue expression patterns and the limitations of extrapolating in vitro efficiency to in vivo dominance.
2. Use of mixed H_2MCZ isomers. Functional assays rely on synthetic o/p H_2MCZ mixtures containing residual MCZ. While acknowledged in the Discussion, this limitation should be more explicitly highlighted in the Results when interpreting MIC and DprE1 inhibition data.
3. H_2MCZ as a drug reservoir. The proposal that H_2MCZ acts as a pharmacokinetic reservoir is interesting but remains partly speculative given its instability. This point should be framed more cautiously.
4. Clinical implications of PTGR inhibition. The suggestion of pharmacologically inhibiting PTGRs to boost MCZ exposure should be clearly positioned as proof-of-concept only, given the known immunomodulatory roles of prostaglandin metabolism.
Minor comments
• Some sections of the enzyme screening Results could be shortened with greater reliance on Supplementary Tables.
• Figure legends (notably Figure 5) would benefit from a clearer indication of MCZ vs H_2MCZ concentrations.

The manuscript is strong and of high interest; addressing the points above will further strengthen clarity and interpretation without requiring additional major experiments.

Referee #2 (Remarks for Author):

Briki et al. report the answer to a question that has long been asked for antimycobacterial benzothiazinones, viz. how the surprise metabolites Hydride Meisenheimer Complexes, are being formed. The experimentally tested a number of human candidate enzymes, identifying prostaglandin reductases PTGR2 (and PTGR1) to be responsible. They also excluded gut bacteria as responsible. In another part of the study, they test the antimycobacterial potency of the metabolite against the parent BTZ, macozinone, in vitro and in vivo, convincingly proving that the BTZ is more potent, but the metabolite has some potential through oxidative reversal to the BTZ.
The data presentation lacks nothing, the discussion of the literature encompasses all relevant sources.

The paper definitely merits publication since two BTZs are in clinical studies, so this information should become public soon.

However, a discussion with experimental proof should be added on the odd finding that the metabolites are more potent than the BTZ in inhibiting the mycobacterial target enzyme, DprE1 (p. 16 l. 349 f). Is this connected to the redox status of the medium and particularly DprE1? Does DprE1 catalyse quick oxidation of H2MCZ to MCZ on site (in the enzyme pocket)? Answering this may guide future efforts to design a BTZ that is not so easily reduced metabolically.

Referee #3 (Comments on Novelty/Model System for Author):

In this manuscript, the authors identify human prostaglandin reductases PTGR1 and PTGR2 as the enzymes responsible for the in vivo dearomatization of the benzothiazinone antitubercular drug macozinone (PBTZ169) into a Hydride-Meisenheimer complex (H2MCZ). This work resolves a long standing mechanistic question in benzothiazinone pharmacology and reveals a previously unrecognized host mediated drug inactivation pathway involving enzymes classically associated with prostaglandin metabolism. By combining enzymology, inhibitor profiling, microbiological assays, and ex vivo analyses of clinical samples, the authors convincingly demonstrate that PTGR1/2 mediated dearomatization reduces antimycobacterial efficacy and that macozinone itself - rather than its reduced metabolite - is the dominant active species in humans. The study is conceptually novel, mechanistically rigorous, and highly relevant to tuberculosis drug development and host-drug interaction biology. It fits very well within the scope of EMBO Molecular Medicine.

Major Points
1. Relative in vivo contribution of PTGR1 versus PTGR2; while PTGR2 appears more efficient in vitro, the manuscript would benefit from a clearer discussion of the likely relative contribution of PTGR1 and PTGR2 in vivo, taking into account tissue distribution, expression levels, and potential interindividual variability.

2. Use of mixed H2MCZ isomers in functional assays; the limitation arising from the use of synthetic H2MCZ containing both ortho and para isomers is well acknowledged. Nevertheless, it would be helpful to more explicitly caution readers that functional conclusions derived from these assays may not fully reflect the behavior of the physiologically relevant para H2MCZ isomer.

3. Scope of generalization; a brief comment on whether PTGR mediated dearomatization might extend to other nitroaromatic or benzothiazinone derivatives would further strengthen the broader impact of the study.

Minor Points
4. Some figures are information dense (notably Figures 5 and 6); minor improvements in labeling or figure legends could enhance readability.

5. The term "virtual alpha, beta-unsaturated carbonyl" is chemically accurate but may benefit from a short explanatory clarification for a broader readership.

Referee #3 (Remarks for Author):

This is a strong and elegant study that provides a clear mechanistic advance with direct translational implications. The manuscript is well written, the conclusions are well supported by the data, and only minor clarifications are required before publication in EMBO Molecular Medicine.

For clarity, Editorial and Reviewers' comments are reported in **bold**, directly followed by author's answers.

Referee #1 (Remarks for Author):

This manuscript by Briki et al. identifies human prostaglandin reductases PTGR1 and PTGR2 as the enzymes responsible for the NADPH-dependent dearomatization of the antitubercular drug macozinone (PBTZ169) into its Hydride Meisenheimer complex (H_2MCZ). The study further demonstrates that this metabolic conversion reduces antimycobacterial activity and can be modulated by known PTGR inhibitors. The work addresses a long-standing question in benzothiazinone pharmacology and reveals a previously unrecognized, noncanonical xenobiotic metabolic pathway in humans. The study is technically rigorous, comprehensive, and conceptually novel. Identification of PTGR1/2 as mediators of enzymatic dearomatization represents a significant advance with implications for anti-TB drug development and host-drug interactions. The conclusions are largely well supported by the data.

Author: We are grateful to Reviewer #1 for this very positive feedback on our work.

Major comments

1. PTGR1 vs PTGR2 contribution. The conclusion that PTGR2 is the principal in vivo enzyme is based mainly on recombinant enzyme activity. This should be stated more cautiously, with clearer discussion of tissue expression patterns and the limitations of extrapolating in vitro efficiency to in vivo dominance.

Author: We thank Reviewer #1 for his thoughtful comments. We acknowledge that extrapolating the *in vitro* activity of PTGR1 and PTGR2 to *in vivo* conditions is somewhat of a shortcut, as it might conceivably depend on their respective tissue expression patterns, and on further confounding factors.

Indeed, according to The Human Protein Atlas, PTGR1 is expressed in the cytoplasm of most tissues and is highly abundant in the liver, kidney, and intestine. In contrast, PTGR2 exhibits both cytoplasmic and nuclear expression across most tissues, although it shows low tissue specificity at the RNA expression level.

We have revised our statement to incorporate the aspect of tissue expression as follows (line 426): "The slightly higher activity of PTGR2 than PTGR1 should be weighed against their differential tissue distribution and expression levels throughout the body (see e.g. The Human Protein Atlas (https://www.proteinatlas.org/))."

2. Use of mixed H_2MCZ isomers. Functional assays rely on synthetic o/p H_2MCZ mixtures containing residual MCZ. While acknowledged in the Discussion, this limitation should be more explicitly highlighted in the Results when interpreting MIC and DprE1 inhibition data.

Author: Thank you for this valuable comment. We have smoothed the wording of the text and the limitation explicitly recognized in the results section as follows (line 347): "Of note, MIC values for H_2MCZ may be altered in part by H_2MCZ spontaneously re-oxidizing into MCZ during the incubation process, in addition to the residual MCZ (evaluated at 7% by LC-MS/MS) present in the synthetic H_2MCZ."

For the DprE1 inhibition experiments with H_2MCZ, this is discussed in our response to Reviewer # 2 (see below).

3. H_2MCZ as a drug reservoir. The proposal that H_2MCZ acts as a pharmacokinetic reservoir is interesting but remains partly speculative given its instability. This point should be framed more cautiously.

Author: We acknowledge that at present this hypothesis remains conjectural. We have rephrased the sentence as follows (line 488): "This hypothesis remains unproven at this stage and requires testing by further drug tissue biodisposition studies."

4. Clinical implications of PTGR inhibition. The suggestion of pharmacologically inhibiting PTGRs to boost MCZ exposure should be clearly positioned as proof-of-concept only, given the known immunomodulatory roles of prostaglandin metabolism.

Author: Thank you for your valuable comment. We have completed our statement as follows (line 519): "A feasibility study for boosting MCZ and BTZ-043 exposure by PTGR blockade is certainly warranted as it may allow use of lower doses of the drugs but caution should be taken given the known immunomodulatory roles of prostaglandin metabolism."

Minor comments

• Some sections of the enzyme screening Results could be shortened with greater reliance on Supplementary Tables.

Author: This research effort has spanned approximately eight years, during which we investigated more than eight different metabolic pathways, summarized in Table S1, before ultimately identifying the correct PTGR1/2 pathway. Despite its critical importance, this screening procedure accounts for only one third (*ca* 4 pages) of the Results section. We aimed to demonstrate to readers that our stepwise approach for identifying the reductases was guided by a strong and coherent rationale rather than by a trial-and-error process. This implied occasionally providing a brief introductory explanation for each putative metabolic pathway. However, we have eliminated as many unnecessary details as possible throughout the Results section of the article.

• Figure legends (notably Figure 5) would benefit from a clearer indication of MCZ vs H_2MCZ concentrations.

Author: we agree that the MCZ and H_2MCZ concentration may not be easily seen in the 96 well pictures. We have therefore increased the font size of concentrations that are depicted on the respective wells.

Moreover, we have added in the caption (line 378): "Respective MCZ and H_2MCZ concentrations are reported on the corresponding wells."

The manuscript is strong and of high interest; addressing the points above will further strengthen clarity and interpretation without requiring additional major experiments.

Author: We are pleased that Reviewer 1 acknowledged our tenacious efforts devoted to uncovering this mysterious metabolic pathway.

Referee #2 (Remarks for Author):

Briki et al. report the answer to a question that has long been asked for antimycobacterial benzothiazinones, viz. how the surprise metabolites Hydride Meisenheimer Complexes, are being formed. The experimentally tested a number of human candidate enzymes, identifying prostaglandin reductases PTGR2 (and PTGR1) to be responsible. They also excluded gut bacteria as responsible. In another part of the study, they test the antimycobacterial potency of the metabolite against the parent BTZ, macozinone, in vitro and in vivo, convincingly proving that the BTZ is more potent, but the metabolite has some potential through oxidative reversal to the BTZ.

The data presentation lacks nothing; the discussion of the literature encompasses all relevant sources.

The paper definitely merits publication since two BTZs are in clinical studies, so this information should become public soon.

However, a discussion with experimental proof should be added on the odd finding that the metabolites are more potent than the BTZ in inhibiting the mycobacterial target enzyme, DprE1 (p. 16 l. 349 f). Is this connected to the redox status of the medium and particularly DprE1? Does DprE1 catalyse quick oxidation of H2MCZ to MCZ on site (in the enzyme pocket)? Answering this may guide future efforts to design a BTZ that is not so easily reduced metabolically.

Author: According to our results (line 459), "it is very unlikely that H_2MCZ is rapidly re-oxidised into MCZ by DprE1, as we would then expect to find a similar IC_{50} value for the two compounds. Moreover, DprE1 is known to have a slow kinetic rate, with a k_{cat} for the substrate ranging from 3 to 12 min^{-1} (depending on the electron acceptor). Also, the kinetics of BTZ activation is rather low, for example, the k_{inact} for BTZ043 is 0.6 min^{-1} (Sci Transl Med. 2012, 4(150):150ra121. doi:10.1126/scitranslmed.3004395). In this context, it is improbable that this enzyme can quickly oxidise H_2MCZ."

Furthermore, the slightly higher potency of H_2MCZ is not so surprising (line 453). Examining the crystal structure of the DprE1-MCZ complex (pdb 4NCR, EMBO Mol Med 2014, 6(3):372-83. doi:10.1002/emmm.201303575.) it is evident that the oxygen atom of the benzothiazinone core is positioned in front of Lys134, at a distance of approximately 3 Å. The presence of a negative charge on this oxygen atom in H_2MCZ (see Figure 1B of our article) could lead to an ionic interaction with the lysine amine group, which would stabilise the compound in the active site of DprE1 and consequently increase the enzyme's affinity for the inhibitor. This could clearly explain the lower IC_{50} displayed by H_2MCZ.

These explanations have been incorporated into the Discussion section (lines 456 sq.), accompanied by the 3D structure of the DprE1–MCZ complex that has been added as Figure S4 in the Supplementary Data.

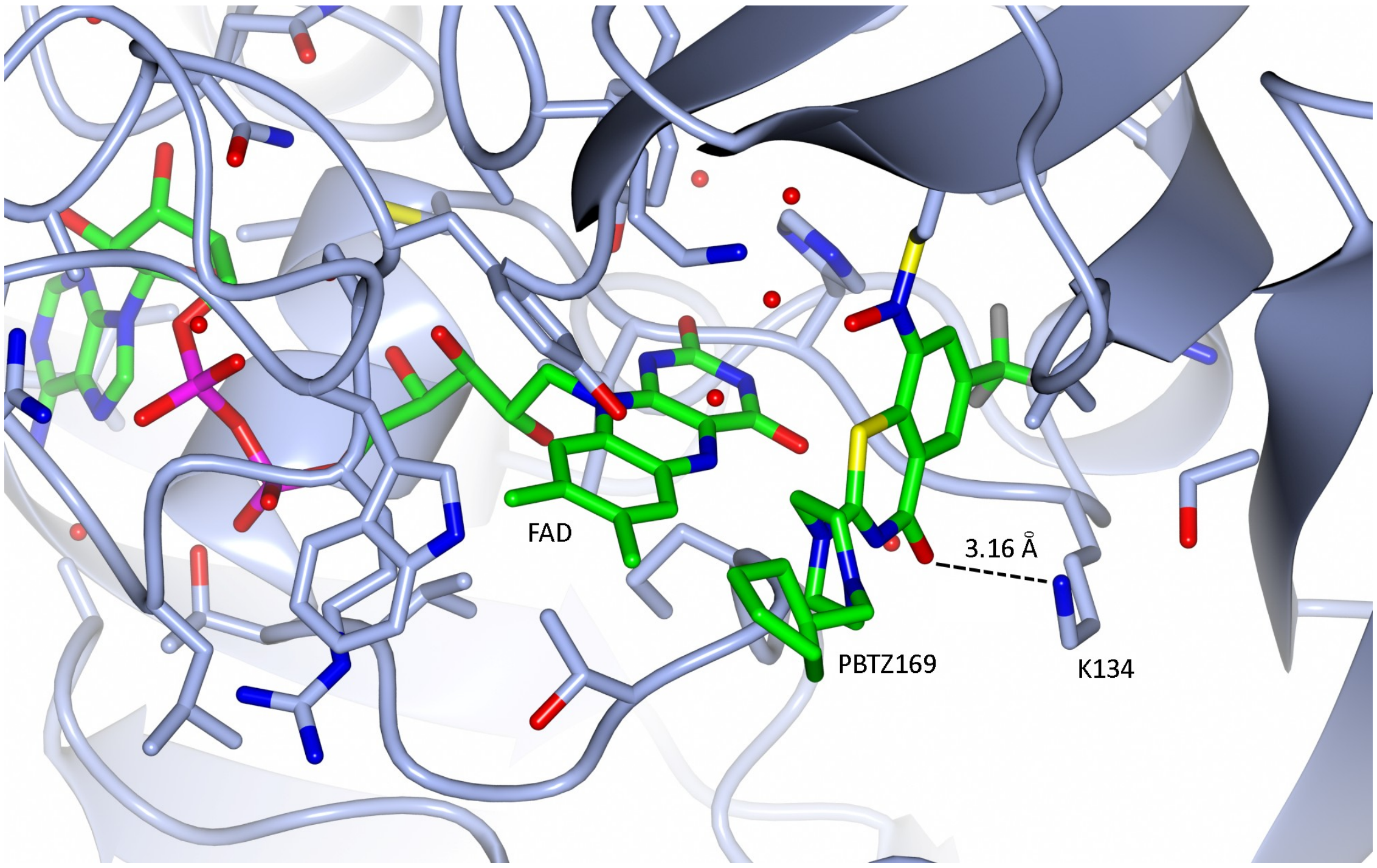

Supplementary Figure S4 | Binding site of PBTZ169 in the DprE1 crystal structure (PDB ID: 4NCR). The oxygen atom of the benzothiazinone core is positioned opposite Lys134 at a distance of 3.16 Å. The negative charge localized on this oxygen atom may facilitate an electrostatic interaction with the ε-amino group of Lys134, potentially stabilizing the inhibitor within the DprE1 active site and thereby enhancing the enzyme's affinity for PBTZ169. The image was generated using CCP4MG version 2.10.11.

Referee #3 (Comments on Novelty/Model System for Author):

In this manuscript, the authors identify human prostaglandin reductases PTGR1 and PTGR2 as the enzymes responsible for the in vivo dearomatization of the benzothiazinone antitubercular drug macozinone (PBTZ169) into a Hydride-Meisenheimer complex (H2MCZ). This work resolves a long standing mechanistic question in benzothiazinone pharmacology and reveals a previously unrecognized host mediated drug inactivation pathway involving enzymes classically associated with prostaglandin metabolism. By combining enzymology, inhibitor profiling, microbiological assays, and ex vivo analyses of clinical samples, the authors convincingly demonstrate that PTGR1/2 mediated dearomatization reduces antimycobacterial efficacy and that macozinone itself - rather than its reduced metabolite - is the dominant active species in humans. The study is conceptually novel, mechanistically rigorous, and highly relevant to tuberculosis drug development and host-drug interaction biology. It fits very well within the scope of EMBO Molecular Medicine.

Major Points

1. Relative in vivo contribution of PTGR1 versus PTGR2; while PTGR2 appears more efficient in vitro, the manuscript would benefit from a clearer discussion of the likely relative contribution of PTGR1 and PTGR2 in vivo, taking into account tissue distribution, expression levels, and potential interindividual variability.

Author: This thoughtful comment from Reviewer #3 echoes the remarks made by Reviewer #1. Again, we acknowledge that directly extrapolating the *in vitro* activities of PTGR1 and PTGR2 to *in vivo* conditions

overlooks important factors such as tissue distribution, expression levels, and potential interindividual variability. We have revised the text accordingly (see above, our previous answer to Reviewer #1).

2. Use of mixed H2MCZ isomers in functional assays; the limitation arising from the use of synthetic H2MCZ containing both ortho and para isomers is well acknowledged. Nevertheless, it would be helpful to more explicitly caution readers that functional conclusions derived from these assays may not fully reflect the behavior of the physiologically relevant para-H2MCZ isomer.

Author: We acknowledge this limitation in all assays performed using the synthetic mixture (see line 509). In fact, our initial aim was to evaluate the individual para-H_2MCZ isomer in the incubation assays. However, as stated in the manuscript, our attempts to isolate para-H_2MCZ from the synthetic mixture by semi-preparative HPLC were unsuccessful, as a substantial fraction of the isolated compound was found to readily reoxidize into MCZ.

Importantly, in Figure 5C–E (incubation of MCZ with the pure enzyme PTGR2) and Figure 6B–D (*ex vivo* incubation assays using samples from volunteers), only the naturally occurring para-H_2MCZ is present. This observation suggests that these biologically relevant conditions, wherein only para-H_2MCZ is formed, most likely reflect the *in vivo* situation.

These remarks have been incorporated into the Discussion section (line 505 sq.): “Functional conclusions derived from the *in vitro* assays may thus not fully reflect the behavior of the physiologically relevant para-H_2MCZ isomer. However, in Figure 5C–E (incubation of MCZ with the pure enzyme PTGR2) and Figure 6B–D (*ex vivo* incubation assays using samples from volunteers), only the naturally occurring p-H_2MCZ is present. This observation suggests that these biologically relevant conditions, wherein only p-H_2MCZ is formed, most likely reflect the *in vivo* situation.”

3. Scope of generalization; a brief comment on whether PTGR mediated dearomatization might extend to other nitroaromatic or benzothiazinone derivatives would further strengthen the broader impact of the study.

Author: we have added these important aspects at the end of the discussion (line 523): “Overall, the unprecedented PTGR-mediated dearomatization described for macozinone also extends to BTZ-043, suggesting that this biotransformation may be a general feature affecting the whole family of nitroaromatic benzothiazinones.”

Minor Points

4. Some figures are information dense (notably Figures 5 and 6); minor improvements in labeling or figure legends could enhance readability.

We have improved the labelling of the figures, notably by increasing the font size of concentrations of MCZ and H_2MCZ that are depicted on the respective wells. We believe that this helps for better readability.

5. The term "virtual alpha, beta-unsaturated carbonyl" is chemically accurate but may benefit from a short explanatory clarification for a broader readership.

Author: we have briefly explained the chemical structure as follows (line 264): “(i.e., a ketone containing a carbon–carbon double bond adjacent to the carbonyl group, where the carbon directly next to the carbonyl carbon is the α-carbon and the following one is the β-carbon).”

Referee #3 (Remarks for Author):

This is a strong and elegant study that provides a clear mechanistic advance with direct translational implications. The manuscript is well written, the conclusions are well supported by the data, and only minor clarifications are required before publication in EMBO Molecular Medicine.

Author: We appreciate this very supportive feed-back of Reviewer #3.

22nd Jul 2026

Dear Prof. Decosterd,

Thank you for submitting your revised manuscript to EMBO Molecular Medicine. Your manuscript will be ready for acceptance pending the following final amendments:

1) Authors: We note name discrepancy in the manuscript and in our submission system: Olga Ryabova vs. Olga Riabova. Please correct.
2) Title: Please consider revising the title to better highlight the main findings, e.g. "Prostaglandin reductases-mediated dearomatization inactivates the antitubercular drug macozinone".
3) Abstract: I have gone through your text and revised it (see below). Please review it for accuracy and amend as you see fit:

Macozinone (MCZ; PBTZ169) is a clinical-stage benzothiazinone antitubercular agent that covalently inhibits the essential mycobacterial flavoenzyme DprE1. Although MCZ undergoes reductive dearomatization to H2MCZ, a Hydride Meisenheimer Complex and the major circulating metabolite in humans, the enzymes responsible and the consequences for therapeutic efficacy have remained unknown. Here, we identify the NADPH-dependent human prostaglandin reductases PTGR1 and PTGR2 as the enzymes catalyzing this reaction. PTGR1/2-mediated dearomatization represents a previously unrecognized host-mediated drug metabolism pathway that results in pharmacological inactivation. Although H2MCZ may constitute a transient reactive intermediate, ex vivo and cellular studies demonstrate that it does not contribute to DprE1 inhibition, indicating that PTGR1/2-mediated metabolism abolishes antimycobacterial activity. Pharmacological inhibition of PTGR1 and PTGR2 with diclofenac, indomethacin, dicumarol, or the selective inhibitor PTGR2-IN-1 suppresses H2MCZ formation and partially restores MCZ activity in vitro. Together, these findings reveal a novel mechanism of human drug metabolism and identify prostaglandin reductases as potential therapeutic targets for enhancing benzothiazinone exposure and efficacy.

4) Figures: Please upload each figure as one high resolution file and one page per figure. Please check the figure formatting guidelines here: https://media.springernature.com/original/springer-cms/rest/v1/content/27825798/data/v1
5) Author checklist: Please submit a complete checklist. https://www.embopress.org/pb-assets/embo-site/EMBO%20Press%20Author%20Checklist-1642513524327.xlsx
6) In the main manuscript file, please do the following:
- Please address all comments suggested by our data editors listed below:
o Figure legends:
1. Please note that information related to n is missing in the legends of figures 4A,B; 5A,B,D,E; 6C; S1-A,B.
2. Please note that n=2 in figures 2B,C.
3. Please note that the error bars are not defined in the legends of figures 4A,B; 5A,B,D,E; 6C.
- In "Disclosure and competing interests statement" please state employments in biotech companies (affiliations a, e and f).
7) Funding: Please merge with the Acknowledgments and make sure that information about all sources of funding are complete in both our submission system and in the manuscript. The European Union's Horizon 2020 Research and Innovation Programme, EFPIA, Global Alliance for TB Drug Development Non-Profit Organisation, Bill & Melinda Gates Foundation, and University of Dundee are currently missing in our submission system.
8) Appendix: Please rename the file with the suppl. information to "Appendix" and upload it as a PDF. Correct the nomenclature of the figures to "Appendix Figure S1" etc. and of the table to "Appendix Table S1". Remove the reagents and tools table and upload it as a separate .doc file. The supplementary methods should be renamed "Appendix Supplementary Methods". Move the sections 2, 3 and 5 to the main Methods section in the manuscript file. Only the section 4 - MS/MS methods should stay in the appendix. Add a table of contents on the title page of the appendix, including page numbers. Please update the callouts in the main manuscript file.
9) Source data: Please remove the files for schematics, they are not needed.
10) The Paper Explained: Please provide "The Paper Explained" and add it to the main manuscript text. Please check any of recently published EMBO Mol Med research articles for reference.
11) Synopsis: Every published paper now includes a 'Synopsis' to further enhance discoverability. Synopses are displayed on the journal webpage and are freely accessible to all readers. They include separate synopsis image and synopsis text.
- Synopsis image: Please provide a visual abstract as a high-resolution jpeg file exactly 550 px-wide x 300 pixels high to illustrate your article.
- Synopsis text: Please provide a short standfirst (maximum of 300 characters, including space) as well as 2-5 one sentence bullet points that summarise the paper as a .doc file. Please write the bullet points to summarise the key NEW findings. They should be designed to be complementary to the abstract - i.e. not repeat the same text. We encourage inclusion of key acronyms and quantitative information (maximum of 30 words / bullet point). Please use the passive voice.
- Please check your synopsis text and image before submission with your revised manuscript. Please be aware that in the proof stage minor corrections only are allowed (e.g., typos).
12) As part of the EMBO Publications transparent editorial process (see our Editorial at http://embomolmed.embopress.org/content/2/9/329), EMBO Molecular Medicine will publish online a Review Process File (RPF) to accompany accepted manuscripts. This file will be published in conjunction with your paper and will include the anonymous

referee reports, your point-by-point response and all pertinent correspondence relating to the manuscript. Let us know if you want to remove or not any figures from it prior to publication. Please note that the Authors checklist will be published at the end of the RPF.
13) Please provide a point-by-point letter INCLUDING my comments as well as the reviewer's reports and your detailed responses (as Word file).

I look forward to reading a new revised version of your manuscript as soon as possible.

Yours sincerely,

Zeljko Durdevic

Zeljko Durdevic
Senior Editor
EMBO Molecular Medicine

*** Instructions to submit your revised manuscript ***

*** PLEASE NOTE *** As part of the EMBO Publications transparent editorial process initiative (see our Editorial at https://www.embopress.org/doi/pdf/10.1002/emmm.201000094), EMBO Molecular Medicine will publish online a Review Process File to accompany accepted manuscripts.

In the event of acceptance, this file will be published in conjunction with your paper and will include the anonymous referee reports, your point-by-point response and all pertinent correspondence relating to the manuscript. If you do NOT want this file to be published, please inform the editorial office at contact@embomolmed.org.

*****

When preparing your revised manuscript, please refer to our guidelines: https://link.springer.com/journal/44321/submission-guidelines#cms-Revised-submissions. We perform an initial quality control of all revised manuscripts before re-review; failure to include requested items will delay the evaluation of your revision.

We require:

1) A .docx formatted version of the manuscript text (including legends for main figures, EV figures and tables). Please make sure that the changes are highlighted to be clearly visible.

2) Individual production quality figure files as .eps, .tif, .jpg (one file per figure). For guidance, download the 'Figure Guide PDF': https://media.springernature.com/original/springer-cms/rest/v1/content/27825798/data/v1.

3) A .docx formatted letter INCLUDING the reviewers' reports and your detailed point-by-point responses to their comments. As part of the EMBO Press transparent editorial process, the point-by-point response is part of the Review Process File (RPF), which will be published alongside your paper.

4) A complete author checklist, which you can download from our author guidelines. Please insert information in the checklist that is also reflected in the manuscript. The completed author checklist will also be part of the RPF.

5) Please note that all corresponding authors are required to supply an ORCID ID for their name upon submission of a revised manuscript.

6) It is mandatory to include a 'Data Availability' section after the Materials and Methods. Before submitting your revision, primary datasets produced in this study need to be deposited in an appropriate public database, and the accession numbers and database listed under 'Data Availability'. Please remember to provide a reviewer password if the datasets are not yet public.

In case you have no data that requires deposition in a public database, please state so in this section. Note that the Data Availability Section is restricted to new primary data that are part of this study.

7) For data quantification: please specify the name of the statistical test used to generate error bars and P values, the number (n) of independent experiments (specify technical or biological replicates) underlying each data point and the test used to calculate p-values in each figure legend. The figure legends should contain a basic description of n, P and the test applied. Graphs must include a description of the bars and the error bars (s.d., s.e.m.).

8) At EMBO Press we ask authors to provide source data for the main manuscript figures. You will receive a separate email with instructions for providing source data with your revised manuscript, including how to upload and organize the files.

9) Our journal encourages inclusion of *data citations in the reference list* to directly cite datasets that were re-used and obtained from public databases. Data citations in the article text are distinct from normal bibliographical citations and should directly link to the database records from which the data can be accessed. In the main text, data citations are formatted as follows: "Data ref: Smith et al, 2001" or "Data ref: NCBI Sequence Read Archive PRJNA342805, 2017". In the Reference list, data citations must be labeled with "[DATASET]". A data reference must provide the database name, accession number/identifiers and a resolvable link to the landing page from which the data can be accessed at the end of the reference.

10) We replaced Supplementary Information with Expanded View (EV) Figures and Tables that are collapsible/expandable online. A maximum of 5 EV Figures can be typeset. EV Figures should be cited as 'Figure EV1, Figure EV2" etc... in the text and their respective legends should be included in the main text after the legends of regular figures.

- For the figures that you do NOT wish to display as Expanded View figures, they should be bundled together with their legends in a single PDF file called *Appendix*, which should start with a short Table of Content. Appendix figures should be referred to in the main text as: "Appendix Figure S1, Appendix Figure S2" etc.

- Additional Tables/Datasets should be labeled and referred to as Table EV1, Dataset EV1, etc. Legends have to be provided in a separate tab in case of .xls files. Alternatively, the legend can be supplied as a separate text file (README) and zipped together with the Table/Dataset file.

11) The paper explained: EMBO Molecular Medicine articles are accompanied by a summary of the articles to emphasize the major findings in the paper and their medical implications for the non-specialist reader. Please provide a draft summary of your article highlighting

- the medical issue you are addressing,

- the results obtained and

- their clinical impact.

This may be edited to ensure that readers understand the significance and context of the research. Please refer to any of our published articles for an example.

12) Author contributions: You will be asked to provide CRediT (Contributor Role Taxonomy) terms in the submission system. These replace a narrative author contribution section in the manuscript.

13) A Conflict of Interest statement should be provided in the main text.

14) Every published paper includes a 'Synopsis' to further enhance discoverability. Synopses are displayed on the journal webpage and are freely accessible to all readers. They include a short stand first (maximum of 300 characters, including space) as well as 2-5 one-sentences bullet points that summarizes the paper. Please write the bullet points to summarize the key NEW findings. They should be designed to be complementary to the abstract - i.e. not repeat the same text. We encourage inclusion of key acronyms and quantitative information (maximum of 30 words / bullet point). Please use the passive voice. Please attach these in a separate file or send them by email, we will incorporate them accordingly.

Please also suggest a striking image or visual abstract to illustrate your article as a PNG file 550 px wide x 300 px high.

15) Include a Reagents and Tools Table as part of the Methods section, which can be downloaded from our author guidelines.

EMBO Molecular Medicine has a "scooping protection" policy, whereby similar findings that are published by others during review or revision are not a criterion for rejection. Should you decide to submit a revised version, I do ask that you get in touch after three months if you have not completed it, to update us on the status.

*Additional important information regarding Figures

Each figure should be given in a separate file and should have the following resolution:
Graphs 800-1,200 DPI
Photos 400-800 DPI
Colour (only CMYK) 300-400 DPI"

Figures are not edited by the production team. All lettering should be the same size and style; figure panels should be indicated by capital letters (A, B, C etc). Gridlines are not allowed except for log plots. Figures should be numbered in the order of their appearance in the text with Arabic numerals. Each Figure must have a separate legend and a caption is needed for each panel.

*Additional important information regarding figures and illustrations can be found at
https://media.springernature.com/original/springer-cms/rest/v1/content/27825798/data/v1

***** Reviewer's comments *****

Referee #1 (Remarks for Author):

I thank the authors for their thorough and thoughtful responses to all comments raised during the review process. The revised manuscript has been substantially improved, and the authors have adequately addressed the concerns regarding the interpretation of PTGR1 versus PTGR2 contributions, the limitations associated with the use of mixed H_2MCZ isomers, the discussion of H_2MCZ as a potential drug reservoir, and the translational implications of PTGR inhibition. The revisions have strengthened the clarity and balance of the manuscript without requiring additional experimental work.

The authors have also incorporated several helpful clarifications throughout the Results and Discussion sections and improved the presentation of the figures, which further enhances the readability and interpretation of the study. The manuscript remains a technically rigorous, conceptually novel, and important contribution to the field of tuberculosis drug discovery and host-drug metabolism.

I am satisfied with the revisions and have no further comments.

Referee #3 (Comments on Novelty/Model System for Author):

Technical quality: The study is technically rigorous and employs complementary biochemical, microbiological, pharmacological, and ex vivo approaches. The conclusions are well supported by the presented data.

Novelty: The identification of PTGR1 and PTGR2 as the enzymes responsible for benzothiazinone dearomatization resolves a long-standing mechanistic question and reveals a previously unrecognized host-mediated drug inactivation pathway.

Medical impact: The findings are relevant for tuberculosis drug development and provide mechanistic insight into host-dependent drug metabolism. While the immediate translational impact is moderate, the work may inform future optimization of benzothiazinone-based therapies.

Adequacy of model system: The selected model systems, including recombinant enzymes, human tissues, and microbiological assays, are appropriate and sufficient to support the main conclusions.

Referee #3 (Remarks for Author):

The authors have addressed my previous comments satisfactorily. The revised manuscript now presents a more balanced interpretation regarding the relative contributions of PTGR1 and PTGR2, explicitly discusses the limitations associated with the H_2MCZ isomer mixture, and appropriately moderates speculative statements. I have no further concerns and support publication.

The authors addressed the remaining editorial issues.

8th Sep 2026

Dear Prof. Decosterd,

We are pleased to inform you that your manuscript is accepted for publication and is now being sent to our publisher to be included in the next available issue of EMBO Molecular Medicine.

Your manuscript will be processed for publication by EMBO Press. It will be copy edited and you will receive page proofs prior to publication. Please note that you will be contacted by Springer Nature Author Services to complete licensing and payment information.

You may qualify for financial assistance for your publication charges - either via a Springer Nature fully open access agreement or an EMBO initiative. Check your eligibility: https://link.springer.com/journal/44321/how-to-publish-with-us

Should you be planning a Press Release on your article, please notify the production team via this form in order to coordinate publication and release dates: https://forms.cloud.microsoft/e/eH4A8JVP6g

If you have any questions, please do not hesitate to contact the Editorial Office. Thank you for your contribution to EMBO Molecular Medicine.

Yours sincerely,
Zeljko Durdevic

Zeljko Durdevic
Senior Editor
EMBO Molecular Medicine

>>> Please note that it is EMBO Molecular Medicine policy for the transcript of the editorial process (containing referee reports and your response letter) to be published as an online supplement to each paper. If you do NOT want this, you will need to inform the Editorial Office via email immediately. More information is available here: https://link.springer.com/partners/embo-press/editorial-policies#Peer%20review

EMBO Press Author Checklist

Decosterd Laurent A
EMBO Molecular Medicine
Manuscript Number: EMM-2026-23488

USEFUL LINKS FOR COMPLETING THIS FORM
The EMBO Journal - Author Guidelines
EMBO Reports - Author Guidelines
Molecular Systems Biology - Author Guidelines
EMBO Molecular Medicine - Author Guidelines

Reporting Checklist for Life Science Articles (updated January
This checklist is adapted from Materials Design Analysis Reporting (MDAR) Checklist for Authors. MDAR establishes a minimum set of requirements in transparent reporting in the life sciences (see Statement of Task: 10.31222/osf.io/9sm4x). Please follow the journal's guidelines in preparing your
Please note that a copy of this checklist will be published alongside your article.

Abridged guidelines for figures
1. Data
The data shown in figures should satisfy the following conditions:
- the data were obtained and processed according to the field's best practice and are presented to reflect the results of the experiments in an accurate and unbiased manner.
- ideally, figure panels should include only measurements that are directly comparable to each other and obtained with the same assay.
- plots include clearly labeled error bars for independent experiments and sample sizes. Unless justified, error bars should not be shown for technical
- if n<5, the individual data points from each experiment should be plotted. Any statistical test employed should be justified.
- Source Data should be included to report the data underlying figures according to the guidelines set out in the authorship guidelines on Data

2. Captions
Each figure caption should contain the following information, for each panel where they are relevant:
- a specification of the experimental system investigated (eg cell line, species name).
- the assay(s) and method(s) used to carry out the reported observations and measurements.
- an explicit mention of the biological and chemical entity(ies) that are being measured.
- an explicit mention of the biological and chemical entity(ies) that are altered/varied/perturbed in a controlled manner.
- the exact sample size (n) for each experimental group/condition, given as a number, not a range;
- a description of the sample collection allowing the reader to understand whether the samples represent technical or biological replicates (including how many animals, litters, cultures, etc.).
- a statement of how many times the experiment shown was independently replicated in the laboratory.
- definitions of statistical methods and measures:
  - common tests, such as t-test (please specify whether paired vs. unpaired), simple χ2 tests, Wilcoxon and Mann-Whitney tests, can be unambiguously identified by name only, but more complex techniques should be described in the methods section;
  - are tests one-sided or two-sided?
  - are there adjustments for multiple comparisons?
  - exact statistical test results, e.g., P values = x but not P values < x;
  - definition of 'center values' as median or average;
  - definition of error bars as s.d. or s.e.m.

Please complete ALL of the questions below.
Select "Not Applicable" only when the requested information is not relevant for your study.

Materials

Newly Created Materials	Information included in the manuscript?	In which section is the information available? (Reagents and Tools Table, Materials and Methods, Figures, Data Availability Section)
New materials and reagents need to be available; do any restrictions apply?	Not Applicable	

Antibodies	Information included in the manuscript?	In which section is the information available? (Reagents and Tools Table, Materials and Methods, Figures, Data Availability Section)
For **antibodies** provide the following information:<br>- Commercial antibodies: RRID (if possible) or supplier name, catalogue number and or/clone number<br>- Non-commercial: RRID or citation	Not Applicable	

DNA and RNA sequences	Information included in the manuscript?	In which section is the information available? (Reagents and Tools Table, Materials and Methods, Figures, Data Availability Section)
**Short novel DNA or RNA including primers, probes**: provide the sequences.	Not Applicable	

Cell materials	Information included in the manuscript?	In which section is the information available? (Reagents and Tools Table, Materials and Methods, Figures, Data Availability Section)
**Cell lines:** Provide species information, strain. Provide accession number in repository **OR** supplier name, catalog number, clone number, and/**OR** RRID.	Yes	Material and Methods
**Primary cultures:** Provide species, strain, sex of origin, genetic modification status.	Not Applicable	
Report if the cell lines were recently **authenticated** (e.g., by STR profiling) and tested for mycoplasma contamination.	Not Applicable	

Experimental animals	Information included in the manuscript?	In which section is the information available? (Reagents and Tools Table, Materials and Methods, Figures, Data Availability Section)
**Laboratory animals or Model organisms:** Provide species, strain, sex, age, genetic modification status. Provide accession number in repository **OR** supplier name, catalog number, clone number, **OR** RRID.	Not Applicable	
**Animal observed in or captured from the field:** Provide species, sex, and age where possible.	Not Applicable	
Please detail **housing and husbandry conditions**.	Not Applicable	

Plants and microbes	Information included in the manuscript?	In which section is the information available? (Reagents and Tools Table, Materials and Methods, Figures, Data Availability Section)
**Plants:** provide species and strain, ecotype and cultivar where relevant, unique accession number if available, and source (including location for collected wild specimens).	Not Applicable	
**Microbes:** provide species and strain, unique accession number if available, and source.	Yes	Material and Methods

Human research participants	Information included in the manuscript?	In which section is the information available? (Reagents and Tools Table, Materials and Methods, Figures, Data Availability Section)
If collected and within the bounds of privacy constraints report on age, sex and gender or ethnicity for all study participants.	Yes	Material and Methods

Core facilities	Information included in the manuscript?	In which section is the information available? (Reagents and Tools Table, Materials and Methods, Figures, Data Availability Section)
If your work benefited from core facilities, was their service mentioned in the acknowledgments section?	Not Applicable	

Design

Study protocol	Information included in the manuscript?	In which section is the information available? (Reagents and Tools Table, Materials and Methods, Figures, Data Availability Section)
If study protocol has been **pre-registered, provide DOI in the manuscript**. For clinical trials, provide the trial registration number **OR** cite DOI.	Yes	Material and Methods
Report the **clinical trial registration number** (at ClinicalTrials.gov or equivalent), where applicable.	Yes	Material and Methods

Laboratory protocol	Information included in the manuscript?	In which section is the information available? (Reagents and Tools Table, Materials and Methods, Figures, Data Availability Section)
Provide DOI OR other citation details if **external detailed step-by-step protocols** are available.	Not Applicable	

Experimental study design and statistics	Information included in the manuscript?	In which section is the information available? (Reagents and Tools Table, Materials and Methods, Figures, Data Availability Section)
Include a statement about **sample size** estimate even if no statistical methods were used.	Not Applicable	
Were any steps taken to minimize the effects of subjective bias when allocating animals/samples to treatment (e.g. **randomization procedure**)? If yes, have they been described?	Not Applicable	
Include a statement about **blinding** even if no blinding was done.	Not Applicable	
Describe **inclusion/exclusion criteria** if samples or animals were excluded from the analysis. Were the criteria pre-established?<br><br>If sample or data points were omitted from analysis, report if this was due to attrition or intentional exclusion and provide justification.	Not Applicable	
For every figure, are **statistical tests** justified as appropriate? Do the data meet the assumptions of the tests (e.g., normal distribution)? Describe any methods used to assess it. Is there an estimate of variation within each group of data? Is the variance similar between the groups that are being statistically compared?	Not Applicable	

Sample definition and in-laboratory replication	Information included in the manuscript?	In which section is the information available? (Reagents and Tools Table, Materials and Methods, Figures, Data Availability Section)
In the figure legends: state number of times the experiment was **replicated** in laboratory.	Yes	Material and Methods, Figures
In the figure legends: define whether data describe **technical or biological replicates**.	Yes	Material and Methods, Figures

Ethics

Ethics	Information included in the manuscript?	In which section is the information available? (Reagents and Tools Table, Materials and Methods, Figures, Data Availability Section)
Studies involving **human participants**: State details of **authority granting ethics approval** (IRB or equivalent committee(s), provide reference number for approval.	Yes	Material and Methods, Figures
Studies involving **human participants**: Include a statement confirming that **informed consent** was obtained from all subjects and that the experiments conformed to the principles set out in the WMA Declaration of Helsinki and the Department of Health and Human Services Belmont Report.	Yes	Material and Methods,
Studies involving **human participants:** For publication of **patient photos**, include a statement confirming that consent to publish was obtained.	Not Applicable	
Studies involving experimental **animals**: State details of **authority granting ethics approval** (IRB or equivalent committee(s), provide reference number for approval. Include a statement of compliance with ethical regulations.	Not Applicable	
Studies involving **specimen and field samples:** State if relevant **permits** obtained, provide details of authority approving study; if none were required, explain why.	Yes	Material and Methods, Figures, Acknowledgements

Dual Use Research of Concern (DURC)	Information included in the manuscript?	In which section is the information available? (Reagents and Tools Table, Materials and Methods, Figures, Data Availability Section)
Could your study fall under dual use research restrictions? Please check biosecurity documents and list of **select agents and toxins** (CDC): https://www.selectagents.gov/sat/list.htm	Not Applicable	
If you used a select agent, is the security level of the lab appropriate and reported in the manuscript?	Not Applicable	
If a study is subject to dual use research of concern regulations, is the name of the **authority granting approval and reference number** for the regulatory approval provided in the manuscript?	Yes	Material and methods, Acknowledgements

Reporting

The MDAR framework recommends adoption of discipline-specific guidelines, established and endorsed through community initiatives. Journals have their own policy about requiring specific guidelines and recommendations to complement MDAR.

Adherence to community standards	Information included in the manuscript?	In which section is the information available? (Reagents and Tools Table, Materials and Methods, Figures, Data Availability Section)
State if relevant guidelines or checklists (e.g., **ICMJE, MIBBI, ARRIVE, PRISMA**) have been followed or provided.	Not Applicable	
For **tumor marker prognostic studies,** we recommend that you follow the **REMARK** reporting guidelines (see link list at top right). See author guidelines, under 'Reporting Guidelines'. Please confirm you have followed these guidelines.	Not Applicable	
For **phase II and III randomized controlled trials**, please refer to the **CONSORT** flow diagram (see link list at top right) and submit the CONSORT checklist (see link list at top right) with your submission. See author guidelines, under 'Reporting Guidelines'. Please confirm you have submitted this list.	Not Applicable	

Data Availability

Data availability	Information included in the manuscript?	In which section is the information available? (Reagents and Tools Table, Materials and Methods, Figures, Data Availability Section)
Have **primary datasets** been deposited according to the journal's guidelines (see 'Data Deposition' section) and the respective accession numbers provided in the Data Availability Section?	Not Applicable	
Were **human clinical and genomic datasets** deposited in a public access-controlled repository in accordance to ethical obligations to the patients and to the applicable consent agreement?	Not Applicable	
Are **computational models** that are central and integral to a study available without restrictions in a machine-readable form? Were the relevant accession numbers or links provided?	Not Applicable	
If publicly available data were reused, provide the respective **data citations in the reference list**.	Not Applicable	
